# Optimising informed consent for participants in a randomised controlled trial in rural Uganda: a comparative prospective cohort mixed-methods study

**DOI:** 10.1186/s13063-018-3030-8

**Published:** 2018-12-22

**Authors:** J. Ditai, J. Kanyago, M. R. Nambozo, N. M. Odeke, J. Abeso, J. Dusabe-Richards, P. Olupot-Olupot, E. D. Carrol, A. Medina-Lara, M. Gladstone, J. Storr, B. Faragher, A. D. Weeks

**Affiliations:** 1 Sanyu Africa Research Institute (SAfRI), Mbale Regional Referral Hospital, Pallisa-Kumi Road Junction, P.o Box 2190, Mbale, Uganda; 2 Sanyu Research Unit, Department of Women’s and Children’s Health, University of Liverpool, Liverpool Women’s’ Hospital, Crown Street, Liverpool, L8 7SS UK; 3 Department of Paediatrics, Mbale Regional Referral Hospital, Mbale, Uganda; 4 Tropical Clinical Trials Unit, Liverpool School of Tropical Medicine, Pembroke Place, Liverpool, L3 5QA UK; 5 Busitema University, Faculty of Health Sciences, PO Box 1460, Mbale, Uganda; 6 Department of Clinical Infection, Microbiology and Immunology, Institute of Infection and Global Health, University of Liverpool, 8 West Derby Street, Liverpool, L69 7BE UK; 7 International Community Paediatrics, Department of Women’s and Children’s Health, University of Liverpool, Liverpool Women’s’ Hospital, Crown Street, Liverpool, L8 7SS UK; 8 Health Economics Group, University of Exeter, Exeter, UK; 9 S3 Global, London, UK

**Keywords:** Consent, Participant information sheet, Slide show, Video message show, Standard

## Abstract

**Background:**

Poor participant understanding of research information can be a problem in community interventional studies with rural African women, whose levels of illiteracy are high. This study aimed to improve the informed consent process for women living in rural eastern Uganda. We assessed the impact of alternative consent models on participants’ understanding of clinical trial information and their contribution to the informed consent process in rural Uganda.

**Methods:**

The study applied a parallel mixed-methods design for a prospective comparative cohort, nested within a pilot study on the community distribution of an alcohol-based hand rub to prevent neonatal sepsis (BabyGel pilot trial). Women of at least 34 weeks’ pregnancy, suitable for inclusion in the BabyGel pilot trial, were recruited into this study from their homes in 13 villages in Mbale District. As part of the informed consent process, information about the trial was presented using one of three consent methods: standard researcher-read information, a slide show using illustrated text on a flip chart or a video showing the patient information being read as if by a newsreader in either English or the local language. In addition, all women received the patient information sheet in their preferred language. Each information-giving method was used in recruitment for 1 week. Two days after recruitment, women’s understanding of the clinical trial was evaluated using the modified Quality of Informed Consent (QuIC) tool. They were also shown the other two methods and their preference assessed using a 5-point Likert scale. Semi-structured interviews were administered to each participant. The interviews were audio-recorded, transcribed and translated verbatim, and thematically analysed.

**Results:**

A total of 30 pregnant women in their homes participated in this study. Their recall of the trial information within the planned 48 h was assessed for the majority (90%, 27/30). For all three consent models, women demonstrated a high understanding of the study. There was no statistically significant difference between the slide-show message (mean 4.7; standard deviation, SD 0.47; range 4–5), video message (mean 4.9; SD 0.33; range 4–5) and standard method (mean 4.5; SD 0.53; range 4–5; all one-way ANOVA, *p* = 0.190). The slide-show message resulted in the most objective understanding of question items with the highest average QuIC score of 100 points. For women who had been recruited using any of the three models, the slide show was the most popular method, with a mean score for all items of not less than 4.2 (mean 4.8; SD 0.6; range 4–5). Most women (63%, 19/30) preferred the slide-show message, compared with 17% (5/30) and 20% (6/30) for the standard and video messages, respectively. The reasons given included the benefits of having pictures to aid understanding and the logical progression of the information.

**Conclusion:**

Our results from this small study suggest that slide-show messages may be an effective and popular alternative way of presenting trial information to women in rural Uganda, many of whom have little or no literacy.

**Trial registration:**

ISRCTN, ISRCTN67852437. Registered on 18 March 2018.

**Electronic supplementary material:**

The online version of this article (10.1186/s13063-018-3030-8) contains supplementary material, which is available to authorized users.

## Background

Informed consent as an ethical requirement is emphasised in the conduct of research, both locally [1] and internationally [2]. Guidelines state that special attention should be given to the specific information needs of individual potential subjects as well as to the methods used to deliver the research information [2]. However, previous studies have demonstrated poor participant understanding of the research information [3–5]. In Africa, a review indicated that up to 79.9% of trial participants did not understand some key domains of the informed consent [6]. This is partly because of the increasingly lengthy and complex informed consent forms [7], which may make them difficult to comprehend, the process of disclosure of trial information to potential participants and the limited health literacy among the African population [8]. This is exacerbated by the known low levels of education and literacy, and that the information is not in the participant’s primary language, all of which are associated with poor comprehension of the informed consent process.

There have been calls for research into innovative ways to improve the informed consent process [4, 5, 9–11]. Improving participants’ understanding of research information is crucial and may be achieved by exploring new or familiar approaches that promote understanding and ultimately lead to informed consent, even in this vulnerable population. Alternatives recommended for improving the informed consent process include simplifying the written information, adding illustrations and altering the layout to highlight important points [12–14]. Other studies have used additional detailed oral or written information and computer-based enhancement of information provision, like audio-visual presentations [15].

Audio-visual presentations of informed consent appear to improve participant satisfaction with the consent information provided [16]. Although its value as a tool to enhance the informed consent process remains unclear [9], it has been found to improve immediate recall of informed consent information [17]. Nevertheless, all these studies were conducted in high-income countries. The effectiveness of audio-visual interventions adhering to Consolidated Standards of Reporting Trials (CONSORT), has not been conducted in underserved populations in low- and middle-income countries or with people with low literacy.

In this study, we developed audio-visual presentations of the participant information to improve the informed consent process for women of low or no literacy in a low-income setting in rural eastern Uganda. We evaluated whether a video or illustrative slide show improved the participants’ understanding of the study information compared to the standard researcher-read consent process.

## Methods

### Study design

The study applied a parallel mixed-methods design for a prospective comparative cohort, nested within the BabyGel pilot cluster randomised trial.

### Study setting

The study setting was that of the BabyGel pilot trial (reported elsewhere), i.e. homes of pregnant women from villages in Mbale District in rural eastern Uganda. The district has 912 villages, each village had approximately 130 households and about five people lived in each household, 56% of whom are women of reproductive age, as documented in the Mbale District health office records. In this study area, only 6% of women aged 15–49 years had completed education at secondary level or higher, compared to the national average of 33%. Literacy was less than 68% [18].

### Selection of villages

We considered all BabyGel pilot trial villages in which there was ongoing recruitment of pregnant women within the strict 8 weeks’ recruitment period of the BabyGel pilot trial. However, this particular study was introduced in the sixth week and we included only six BabyGel trial villages. We recruited participants before the end of the eighth week. To attain the required sample size for this study, we included seven more villages that were neighbouring the BabyGel pilot trial villages. These non-trial villages were purposively selected based on the availability of pregnant women meeting the original inclusion criteria for the BabyGel pilot trial.

### Study population

Participants included eligible pregnant women prior to recruitment into the BabyGel pilot cluster randomised trial. As in the pilot trial, the confirmed pregnant women had a gestation of 34 weeks or more and were able to speak or understand English or Lumasaba (the local language). We excluded women who had already been recruited into the pilot trial before this nested study.

### Recruitment of participants

**Fig. 1:**
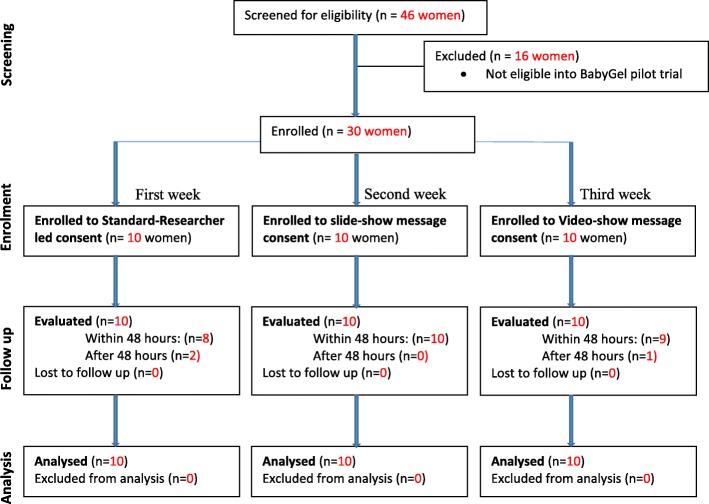
Trial poster showing informed consent information. **a** Presenting the informed consent information. **b** Researcher-read participant information sheet. **c** Slide-show message. **d** Screenshot of the video message

We recruited a total of 30 pregnant women in their homes from 13 villages in the last 3 weeks of BabyGel pilot trial recruitment (Fig. 1). Most of the women (70%, 21/30) were recruited from BabyGel pilot trial villages, comprising 20% (21/103) of the BabyGel recruits. The other nine women were recruited from non-trial villages.

The research midwife visited homes in each village with the aid of the village health team member/worker (VHW) and screened women for eligibility into the BabyGel pilot trial. The 30 recruited women gave initial signed informed consent (on a standard one-page information consent form) for participation both in the BabyGel pilot trial and to undergo a specific form of consent for the BabyGel pilot trial. These women were told during the informed consent process that they would be followed up 48 h (or 2 days) later to test their comprehension of the informed consent information and to identify their preference.

**Fig. 2:**
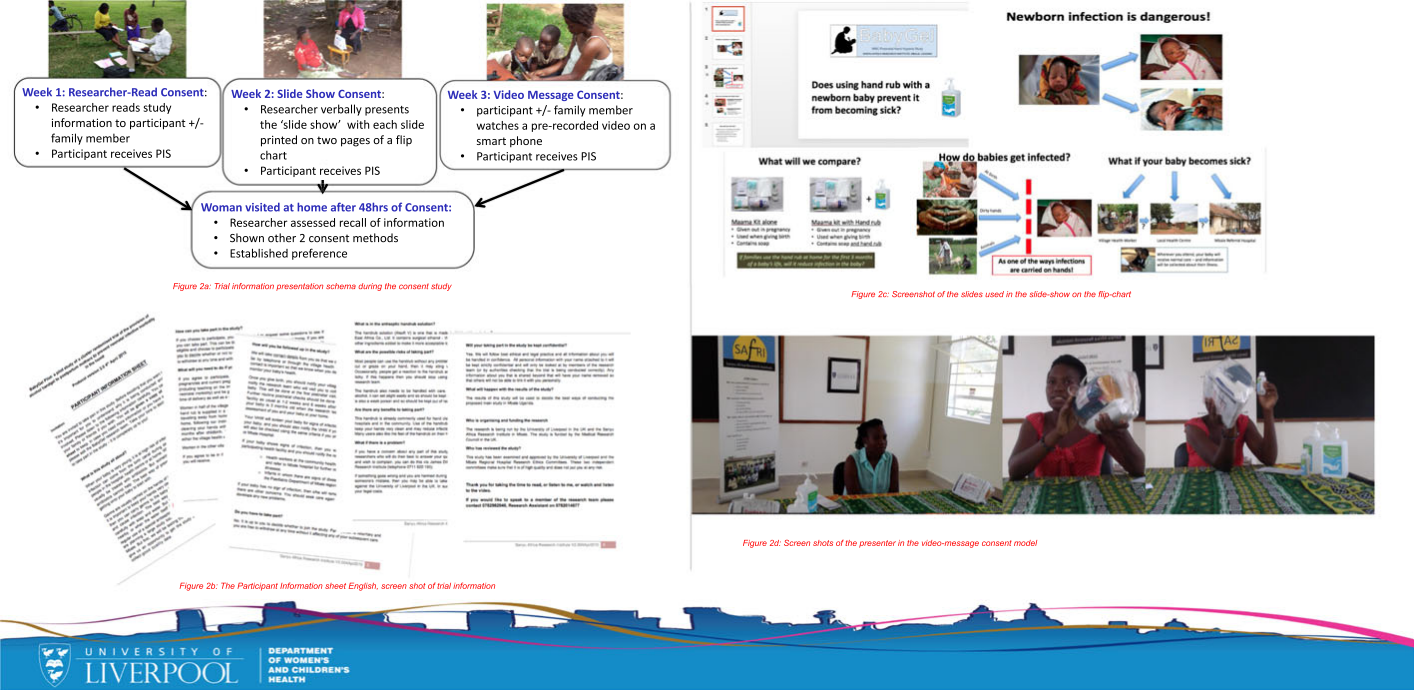
Participant flow in the consent study nested within the BabyGel pilot cluster randomised trial (across 3 weeks). This shows the number of women screened, those eligible and ineligible, the number of women followed up and the number analysed or excluded from the analysis

The three different consent models were administered to eligible pregnant women systematically over the last 3 weeks of recruitment into the BabyGel pilot cluster randomised trial. In each week, before receiving informed consent for entering the trial, study information was presented to each woman according to that week’s consent model. In the first week, the standard researcher-read participant information sheet was used, followed by a slide-show message in the second week and a video message in the last week. as illustrated in Fig. 2a. The standard researcher-read consent method was used to recruit the rest of the participants into the BabyGel pilot trial before the start of the consent nested study.

Each participant could ask questions or discuss issues at any point during the information presentation, which the research assistant responded to. At the end, a copy of the participant information sheet was given to each woman. Those women who agreed to participate in the trial completed a consent form with a signature or thumbprint and also signed by an impartial witness.

#### Researcher-read consent

This is the standard consent model commonly used to provide information to the BabyGel study participants. The research assistants read the study information from the ethically approved participant information sheet (Additional file 1) to each participant in either English or Lumasaba (Fig. 2b).

#### Slide-show message consent

In the second week, instead of research assistants reading out the participant information sheet as in week 1, they presented the study information to each participant orally using the approved 21 slides (Additional file 2) presented on a flip chart (in the style of an A4 ring-bound calendar) with text in bullet points and pictorial illustrations (Fig. 2c). The slide show was in either English or Lumasaba according to the participant’s preference. During the presentation, the participant concurrently read the pre-specified text and watched the pictorials on the reverse of the flip chart. After the presentation, the woman was allowed to see certain slides again if she wished.

#### Video-message consent

In the third week, the research assistant showed the approved study video (Additional file 3) to the eligible women with or without her family members present. The woman watched the video in either English or Lumasaba on a 5-in touchscreen Samsung S4 smartphone as presented by the researchers (Fig. 2d)*.* At the end of the video, the woman was allowed to watch it again if she wished.

### Information consent models

The participant information sheets, the slides in the flip charts and the videos were developed in a rigorous systematic stepwise process (Additional file 4). The lead investigator trained the research assistants on the presentation of the three consent methods using role playing, peer reviews and critiques before participants were enrolled using any of the methods.

### Data collection

Three research assistants (a research midwife and two qualitative researchers) visited each woman in her home 48 h after giving informed consent. Qualitative and quantitative were data collected using Open-Data Collection Kit (ODK) forms installed on Samsung smartphones. These were synced over a wireless internet connection to the online database on the University of Liverpool’s server once the research team returned each evening to the research office in the Sanyu Africa Research Institute.

#### Quantitative data collection

The primary outcome was understanding and recalling information about the BabyGel pilot trial 48 h after being recruited. Data were collected electronically into the modified version of the Quality of Informed Consent (QuIC) case report form on the ODK system operated smartphones (Additional file 5). The form or data tool was designed to measure objective and subjective comprehension of the study information [5]. The research midwife administered the questionnaire face to face. At the start of the evaluation, the woman was instructed to respond to each question or rank her understanding of something as best as she could without consulting the participant information sheet. Correct and incorrect answers were assigned a QuIC score of 100 points and 0 points, respectively [5], and the answers were entered into the ODK electronic data capture system on the smartphone.

After answering questions on comprehension, the woman was shown the other two consent models, which were not used at the time of her recruitment. It was explained to the participant what the two alternative consent models entailed and she was not necessarily taken through the informed consent process again. Each woman was asked to rank the consent models in terms of preference on a 5-point scale of 1 (“I don’t like it all”) to 5 (“I like this most”). Lastly, the final item asked the participant to state her preferred model, giving reasons for her choice. The quantitative data were entered directly into the ODK form on the smartphone.

#### Qualitative data collection

Through semi-structured interviews, two qualitative researchers (MRN and JK) explored aspects of the comprehension of the informed consent messages and the preferences relating to the informed consent process [19]. All responses were audio-recorded and the qualitative researchers entered notes directly into the ODK software on the smartphone.

### Data analysis

All analyses of quantitative variables were conducted using StatsDirect 3 to generate descriptive statistics. Statistical significance was set at the conventional 5% level.

Audio-recorded responses were transcribed and translated verbatim by the study’s main qualitative researcher (MRN). The transcripts were analysed using the inductive thematic approach to qualitative analysis, by describing and analysing patterns within the data [20]. The qualitative researchers (MRN and JK) familiarised themselves with the data by repeatedly reading the transcripts before coding them in detail to ensure all relevant codes were included in the initial pool of codes. The pool of codes was arranged into potential themes based on occurrence, significance and overlap. Together with the lead investigator (JD), the qualitative researchers (MRN and JK) reviewed the themes in relation to the generated codes and the entire dataset. We then agreed on the themes and defined a coding schedule, which was used to code all interviews again to ensure reliability and consistency of coding. QSR NVivo 10 (qualitative analysis software) was used to organise codes and themes.

## Results

### Socio-demographic characteristics

Table 1 shows that about a third (11/30) of the women did not know their exact dates of birth. Most of the women (77%, 23/30) were married while two-thirds were either unemployed or peasant farmers. Half of the women had no formal education or did not complete primary education, which could be an indicator of the level of literacy amongst this population. All the women lived with one or more family members. More than half (60%, 18/30) had family members present while the consent information was being read, presented or shown. The family members present were either children, a husband, a sister, a sister-in-law or a mother-in-law. The VHWs in each village provided research team or midwives with the directions to the homes or households of the potential pregnant women in the corresponding village. The VHW went with the research midwife up to the home of the potential pregnant woman.

**Table 1:** Participant characteristics at recruitment

Consent method	Standard method (*n* = 10)	Slide show (*n* = 11)	Video show (9)	Total
Sample size	10	11	9	30
Dates of recruitment	11–18 November 2016	18–24 November 2016	25 November to 1 December 2016	11 November to 1 December 2016
Dates of follow-up assessment	13–20 November 2016	20–26 November 2016	27 November to 3 December 2016	13 November to 3 December 2016
Age (years): mean (standard deviation) [range]	26 (5.5) [17–36]	24.5 (5.7) [17–33]	22.8 (6.1) [15–35]	24.5 (5.7) [15–36]
Known exact date of birth *n* (%)	3(30)	4(36.7)	4(44.4)	11(36.7)
Marital status:				
Single or widowed *n* (%)	3 (30)	2 (18.2)	2 (22.2)	7 (23.3)
Married *n* (%)	7 (70)	9 (81.8)	7 (77.8)	23 (77.7)
Highest level of education attained:				
No formal education *n* (%)	1 (10)	0	0	1 (3.3)
Did not complete primary education *n* (%)	2 (20)	7 (63.6)	5 (55.6)	14 (46.7)
Completed primary education (Primary Leaving Education (PLE) *n* (%)	2 (20)	1 (9.1)	3 (33.3)	6 (20)
Completed ordinary level education (Uganda Certificate of Education (UCE) *n* (%)	3 (30)	3 (27.3)	1 (11.1)	7 (23.3)
Completed advanced level education or above *n* (%)	2 (20)	0	0	2 (6.7)
Primary occupation				
Housewife *n* (%)	3 (30)	4 (36.4)	3 (33.3)	10 (33.3)
Student *n* (%)	3 (30)	1 (9.1)	1 (11.1)	5 (16.7)
Peasant farmer *n* (%)	3 (30)	5 (45.4)	5 (55.6)	13 (43.3)
Professional *n* (%)	1 (10)	1 (9.1)	0	2 (6.7)
Number of times information was presented:				
Once *n* (%)	9 (90)	10 (90.9)	9 (100)	28 (93.3)
Twice *n* (%)	1 (10)	0	0	1 (3.3)
Thrice *n* (%)	0	1 (9.1)	0	1 (3.3)
Persons present during information:				
Participant only *n* (%)	5 (50)	4 (36.4)	3 (33.3)	12 (40)
Family members* *n* (%)	5 (50)	7 (64.6)	6 (66.7)	18 (60)
Post-recruitment time:				
Within 2 days [48 h] *n* (%)	8 (80)	11 (100)	8 (88.9)	27 (90)
After 2 days [72 h] *n* (%)	2 (20)	0	1 (11.1)	3 (10)

*family members present included children, husband, sister, sister-in-law, Village health team worker (VHW)

### Evaluation of understanding or recall of specific trial information

Table 2 and Fig. 1 show that the majority of the women (90%, 27/30) were assessed for their recall of trial information at the planned 48 h after the original recruitment. The rest (two who received researcher-read consent information and one who received video consent information) were not at home but were assessed within 72 h after recruitment.

**Table 2:** Objective assessment of participants’ recall of trial information 48 h after recruitment

Characteristic	Model of information presentation *n* (%)			Percentage differences (95% CI)		
	Standard method (*n* = 10)	Slide show (*n* = 11)	Video show (*n* = 9)	Slide vs. stand	Video vs. stand	Slide vs. video
How long will you be followed up after giving birth as part of the study?						
3 months (correct option)	10 (100)	11 (100)	9 (100)	–	–	–
Other (incorrect option)	0 (0)	0 (0)	0 (0)	–	–	–
QuIC score	100	100	100	–	–	–
How many villages around Mbale are taking part in this study?						
10 villages (correct option)	10 (100)	11 (100)	9 (100)	–	–	–
Other (incorrect option)	0 (0)	0 (0)	0 (0)	–	–	–
QuIC score	100	100	100	–	–	–
If you have been given some hand gel and it runs out before the end of the study, what do you do?						
Get more supplies from Busiu Health Centre or VHW (correct option)	7 (70)	11 (100)	8 (88.9)	30 (2, 58)	18.9 (−16.1, 53.9)	11.1 (−9.4, 31.6)
Other (incorrect option)	3 (30)	0 (0)	1 (11.1)			
QuIC score	70	100	88.9	30 (2, 58)	18.9 (−16.0, 53.9)	11.1 (−9.4, 31.6)
What should you do once you have given birth?						
Notify village health worker (correct option)	6 (60)	6 (54.5)	8 (88.9)	−5.5 (−47.8, 36.8)	28.9 (−7.7, 65.5)	−34.4 (− 70.3, 1.5)
Other (incorrect option)	4 (40)	5 (45.5)	1 (11.1)			
QuIC score	60	54.5	88.9	−5.5 (−47.8, 36.8)	28.9 (−7.7, 65.5)	−34.4 (− 70.3, 1.5)
What does the hand gel contain?						
Surgical alcohol (correct option)	10 (100)	11 (100)	9 (100)	–	–	–
Other (incorrect option)	0 (0)	0 (0)	0 (0)	–	–	–
QuIC score	100	100	100	–	–	–
Average QuIC score	76.7 (22.5)	84.8 (17.4)	92.6 (14.7)	8.2 (−8.5, 24.8)	15.9 (−1.6, 33.4)	7.7 (−9.4, 24.9)

*CI* confidence interval, *QuIC* Quality of Informed Consent, *VHW* village health team member or worker

Irrespective of the model for presenting information, women demonstrated high accuracy in recalling trial information with an overall average QuIC score of 90.8 points. All participants knew how long they were to be followed up for after giving birth as part of the study, the number of participating villages and the chemical content of the hand gel, but there were some errors for the questions on getting more hand gel and who to contact after giving birth. There was evidence that slide-show consent produced a 30% higher correct recall of what to do when the hand gel runs out before the end of the study compared to the standard researcher-read method (95% confidence interval 2–58%).

Overall, the video message had the highest average QuIC score of 92.6 points, compared to 84.8 for the slide-show message and 76.7 for the standard method (Table 2), but none of these differences was statistically significant (one-way ANOVA, *p* = 0.194).

Table 3 shows the results for the subjective understanding. There was a high overall QuIC score across all three consent models (89 for the standard method, 97 for the slide show and 99 for the video show). The scores for the slide show and the video were similar but both were significantly greater than the score for the standard method (one-way ANOVA, *p* = 0.002). The video consent model had perfect scores for research aims, study duration, alternatives to trial participation and voluntary participation. The slide-show consent model had perfect scores for study duration and voluntary participation, and the standard consent model had a perfect score for study duration only.

**Table 3:** Likert-scale score for the recall of information 48 h or more after recruitment and preferences for consent model

Characteristic	Model of information presentation: mean (SD) [range]			Differences (95% CI)		
	Standard method (*n* = 10)	Slide show (*n* = 11)	Video show (*n* = 9)	Slide show vs. standard	Video show vs. standard	Slide show vs. video show
Understanding of aspects of the cluster randomised trial:						
Research aim (1 = not at all, 5 = very well)	4.3 (0.9) [2, 5]	4.7 (0.47) [4, 5]	5.0 (0.0) [5, 5]	0.4 (−0.2, 1.0)	0.7 (0.1, 1.3)	−0.3 (−0.3, 0.0)
Study duration (1 = not at all, 5 = very well)	5.0 (0.0) [5, 5]	5.0 (0.0) [5, 5]	5.0 (0.0) [5, 5]	–	–	–
Treatments and procedure (1 = not at all, 5 = very well)	3.8 (1.0) [2, 5]	4.7 (0.47) [4, 5]	4.9 (0.33) [4, 5]	0.9 (0.2, 1.6)	0.9 (0.2, 1.6)	−0.2 (−0.4, 0.0)
Alternatives to trial participation (1 = not at all, 5 = very well)	4.1 (0.86) [2, 5]	4.8 (0.40) [4, 5]	5.0 (0.0) [5, 5]	0.7 (0.1, 1.3)	0.9 (0.4, 1.4)	−0.2 (−0.4, 0.0)
Point of contact (1 = not at all, 5 = very well)	4.7 (0.48) [4, 5]	4.9 (0.30) [4, 5]	4.9 (0.33) [4, 5]	0.2 (−0.1, 0.5)	0.2 (−0.2, 0.6)	0.0 (−0.3, 0.3)
Voluntary participation (1 = not at all, 5 = very well)	4.8 (0.42) [4, 5]	5.0 (0.0) [5, 5]	5.0 (0.0) [5, 5]	0.2 (−0.1, 0.5)	0.2 (−0.1, 0.6)	–
Overall study understanding (1 = not at all, 5 = very well)	4.5 (0.53) [4, 5]	4.7 (0.47) [4, 5]	4.9 (0.33) [4, 5]	0.2 (−0.2, 0.6)	0.4 (−0.1, 0.9)	− 0.2 (−0.6, 0.2)
Overall QuIC score	89 (9) [69, 97]	97 (3) [91, 100]	99 (2) [94, 100]	8 (3, 13)	10 (5, 15)	−2 (−7, 3)
Consent model preferred:						
Standard/researcher-read (1 = not at all, 5 = most)	4.0 (0.47) [3, 5]	3.9 (0.94) [3, 5]	3.0 (0.0) [3, 3]	−0.1 (−0.7, 0.5)	−1.0 (−0.7, −1.3)	0.9 (0.3, 1.5)
Slide-show consent (1 = not at all, 5 = most)	4.7 (0.48) [4, 5]	4.2 (0.60) [3, 5]	4.8 (0.44) [4, 5]	−0.5 (−1.0, 0.0)	0.1 (−0.3, 0.5)	−0.6 (−1.1, −0.1)
Video-message consent (1 = not at all, 5 = most)	3.7 (0.82) [2, 5]	4.3 (0.65) [3, 5]	4.2 (0.44) [4, 5]	0.5 (−0.1, 1.1)	0.5 (−0.1, 1.1)	0.1 (−0.4, 0.6)
Which consent model do you prefer most, *n* (%)	5 (16.7)	19 (63.3)	6 (20)	46.6 (27.8, 65.4)	3.3 (−31.5, 38.1)	43.3 (24.1, 62.5)

*CI* confidence interval, *QuIC* Quality of Informed Consent, *SD* standard deviation

### Evaluation of preference of consent model

Table 3 shows that, of the three methods of information giving, women preferred the slide show, which had mean scores of not less than 4.2 (highest mean 4.8, highest standard deviation 0.6, widest range 4–5). The researcher-read method was the least favourite with the lowest mean score (lowest mean 3.0, lowest standard deviation 0.0, narrowest range 3–3). Overall, almost two-thirds of the women (19/30, 63.3%) preferred the slide-show consent message.

### Qualitative results

An analysis of the semi-structured interviews revealed three themes: pictorial illustrations aid understanding, logical progression of information, and ease of understanding for women who are illiterate. In the quotes from the interviews, numbers 3–11 indicate the BabyGel trial villages and letters A–F are the unique codes for each participant within a village.

#### Pictorial illustrations aid understanding

Women liked having pictures in the slides that aided their understanding. The slide-show message had pictures that were explanatory to the trial information, meaning that the participants were able to visualise the process:

You understand it fast because you see pictures. It has pictures that you view and these help you to learn. It is most preferred because it has words and pictures, that you can read and view so it helps you to learn how things are done. (3-A)

With slide show, I see pictures as they are illustrated. … I can easily understand as you read. (10-C)

They have drawn pictures that you see as it is being read to you and so it is easy for you to understand. … It has been read to me and I understood it with the help of pictures. (11-B)

Women also noted that the slide show indicates what is happening in the study. Most women found that the image of a sick child to illustrate sepsis was compelling:

The slide has pictures that show how babies look when they fall sick and I have learnt that it’s good to keep hands clean while looking after the baby.

You see how children fall sick and why it’s important to keep the hands clean. (9-B)

It has shown me how serious it is when the baby falls sick as compared to the researcher-read and video-show message. … I have seen what is there and helps me to understand better because of the pictures. (5-A)

#### The logical progression of information

Many of the participants appreciated the logical flow of information in the slide-show message:

In the slides, you are shown every step. Because the midwife is reading the slides, [and I] am seeing the pictures and their illustrations … it is easy for me to follow. (5-A)

As you were reading it, I was seeing what was taking place. I prefer [the] slide show because it has pictures from beginning up to the end. (8-A)

[It] helps me to relate the picture to the message and know what is going on exactly. (4-E)

#### Making it easy for women who are illiterate to understand

The women preferred simplicity, especially those who had never received any formal education. For example:

Those illustrations are easy to understand. … For me who has not gone to school, [I am] able to understand better with the help of the pictures which are on the slides. (11-F)

In these rural settings, most women are not educated so they can see the pictures and understand them. (3-C)

#### Direct comparisons of the models

Most participants preferred the slide-show message consent compared to the other two consent methods:

It is easier to understand (information presentation with pictures) compared to the researcher-read and the video-show message. What is being read on the slides has been illustrated by pictures compared to the video which was read with not enough illustrations. (3-A)

It has pictures that show you how things are done and so it’s easier to understand it compared to the researcher-read and video-message models. You can ask questions at any point if you have not understood while in the video you cannot ask until the end of the video. It’s because it has pictures that illustrate what is being talked about while for the video it is just hearing the reading and no chance of even asking questions in between.

(9-A)

Those who preferred the video message believed that explanations were being shown in real time and therefore, they stated that it was much easier to understand. They also believed that the language was simple and easy to understand.

It is clearer for me to understand and get what is being said clearly. (11-B)

It’s live and clear … as compared to researcher-read and slide show. (3-E)

These who preferred the researcher-read message mentioned being able to read it by themselves until they understood it:

I can read it by myself and understand it in my own way. (4-B)

## Discussion

To our knowledge, this is the first study to compare video and illustrated participant trial information with the long-established researcher-read method in a rural African setting. Overall, the comprehension of the trial information was high across all three consent models. Our study attempts to address the need to improve informed consent found in other studies assessing QuIC [10–12, 21]. A related study found a high rate of understanding and awareness of study participation, even among the less well educated. It applied a multisource informed consent information system with an enhanced informed consent form, brochure and poster. The information was presented in stages [22]. Furthermore, our study is the first to find a closely related degree of objective understanding with subjective understanding, regardless of the literacy level. Other studies have found that the degree of subjective understanding to be higher than the degree of objective understanding [12]. Our study showed that women are concerned about the condition of their children and pay attention to information relating to the condition, which might have influenced the high rates of understanding of trial and trial information in this population. However, lessons from two HIV clinical trials in Uganda indicate that study participants pay limited attention to study design issues during the consent process except for invasive procedures like blood tests. This study gives another possible explanation for the high rates of understanding in this population. Further, we used only a few questions to assess understanding and the participants may have found them simple. Our finding does not concur with an African study amongst parents who had a significant but varied comprehension of the informed consent process in research activities in northern Ghana [23].

The video message and slide-show message participants ranked their understanding above that of those who received the standard researcher-read model. Previous studies have shown interactivity and multimedia to be effective in promoting individuals’ understanding of and confidence in consent [9, 10, 16, 24], while longer consent forms for clinical trials compromise patient understanding [25]. Though the video show had overall QuIC scores for self-scored understanding just above that of the slide show, most participants preferred the slide-show message, including those who had been recruited using the video show. From the participants’ perspective, women may not have valued the video message as it was simply a recording of the researcher reading out the study information. Also, the benefits of using pictures to aid understanding and the logical progression of the information in the slide show might have made it preferable compared to the other formats. Though this is the first study to report on the use of a slide show and to look at the understanding and preferences of pregnant women in these communities regarding the presentation of trial information, the results concur with previous recommendations [10, 26]. The results for the slide show agree with the lessons from two HIV trials in Uganda that appropriate interactions and communications between participants and researchers are vital for improving participants’ understanding of the informed consent process [27]. Further, the participants were articulate about the slide-show message presentation, suggesting that even women who are illiterate can easily understand the trial information with the aid of the pictures. This demonstrates the power of pictorial communication in information recall.

In this study, women’s ability to engage actively in the informed consent process demonstrates the importance of involving participants in the design of trial information. This is supported by studies on involving patients and the public in constructing trial information together with the research team [28].

It may be challenging for a research team to find equivalent pictures for some of the text due to the technical jargon used for informed consent. We think that some large trials, especially those involving children and new vaccines, may struggle to find appropriate pictures for the slide show of participant information. However, ethics committees would still require all relevant trial information to be incorporated in the slide show.

### Limitations

The small sample size in this study may have increased the risk of the research team being misled by chance differences in the three consent methods. Larger studies would provide more statistical power to explore the factors that improve understanding. Also, participants were not allocated at random to the three methods. Hence, we did not ensure that the participants in the three groups were as similar as possible to each other before the interventions were started. Also, participants’ recall of the information was measured only within 48 h and their recall and understanding after a long time, especially in the long-term follow-up during a trial, may be different.

## Conclusions

Our results suggest that the use of illustrations and diagrams to complement patient information sheets are both effective and acceptable in a rural African setting, but these need to be carefully designed. This small study suggests that study information presented in this way is of comparable effectiveness to other methods, and this method is preferred by participants. We recommend that this format is further explored in larger studies. Ethics committees should ensure that trial information is provided in an acceptable and memorable way, especially in settings with low levels of literacy.

## Additional files

Additional file 1: The approved participant information sheet (zipped PIS english and Lumasaba). (ZIP 48 kb)

Additional file 2: The approved slides (English version and Lumasaba version). (ZIP 12399 kb)

Additional file 3: The approved videos (English version and Lumasaba version). (ZIP 77985 kb)

Additional file 4: The stepwise development process of informed consent models. (DOCX 19 kb)

Additional file 5: Data collection tool: Optimal Informed Consent in the BabyGel pilot Cluster Randomised Trial. (DOCX 123 kb)

## Abbreviations

CI: Confidence interval
CONSORT: Consolidated Standards of Reporting Trials
ODK: Open-Data Collection Kit
QuIC: Quality of Informed Consent
SD: Standard deviation
