# Supplementary material for: Optimising informed consent for participants in a randomised controlled trial in rural Uganda: a comparative prospective cohort mixed-methods study
Source: Trials. 2018 Dec 22;19:699. doi: 10.1186/s13063-018-3030-8 (PMC6304001; doi:10.1186/s13063-018-3030-8)
Supplement: Supplementary file 1 — The approved participant information sheet (zipped PIS english and Lumasaba). (ZIP 48 kb) [file 13063_2018_3030_MOESM1_ESM.zip › PIS Forward Lumasaba.docx]

**Baby Gel:** **Kumusomo khunyowa mukhugesesa kwe khuwa bamayi basalire kamasuso khalimo indali khuhendesa bulwale mubana bafiti mungo**

Protocol version 2.0 4^th^ April 2015

**LUPAPULA LWE KHAMAKHUWA KO MWITUBASI**

**Khukhulanga**

Ulangibwa khukhutubasa mumusomo khuno. Nga ushili khu khalawo ukwitubasa ta, shili shilayi khumanya lwashina kumusomo khuno khutsa khubawo na shina shitsa khubamu. khukhulomba usome khamakhuwa khano ni bubunende. Unyala washikhanikhakho ni be mungo mwowo, namwe basaale nga wakanile. Khane khukhuwe lupapula lwe kamakhuwa khano ubikhe. Nga ukana kamakhuwa kafurakho namwe uli ni birebo bibiindi, khusaba urebe khakhobo khe khureberesa kumusomo khwa BabyGel. Yilla imbuka khukhalawo nga unitubasa mumusomo kuno**-** shili isi uli.

**Kumusomo kuli khusiina?**

Nga umwana wowo ashili umujekhe naabi, ali ni tsikhabi tsingali khufuna buwukha bulela bulwale. Buwukha bubulela bulwale bunyala bwafunikha muntsila isi umwana asalikhilamo nga asalikha, namwe wafuna khubandu babandi mwikangilo namwe mungo. Nga bulwale bwamanyikhile bunyala khukangikha ni kamasunso. Ne imbuka ikindi buuba bubi naabi nga kamasunso shikanyala khuyeta ta. Intsila infura khushingirisa bulwale ili khukhwakamisa buwukha khukhwola khumwana.

Buwukha bwitsila khumakhono khe babandu balinda umwana. Iyo ilomo lwashiina shili shilaya naabi khulinda kamakhono nga kamiliyile nga ulinda umwana. Nga wakhushikhola ta, unyala walelela umwana buwukha khukhwama isi babandu babandi, mubuchafu, namwe mubisolo ate bulela bulwale. Ntsila ifura khulinda kamakhono nga kamamiliu ili khusinga kamakhono khowo ni sabuni ni kametsi. Ne shiino she shili shishyangu ta isi kametsi kakhali shiimi ta, namwe kametsi kamasinye. Ino niye ilomo lwashiina khuli khukhaka khurambisa bushelle kamasunso kejawulo khuakha mungono nga kanyala khushingirisa bulwale mubaana bamakhusalikha. Khuli khurenjarenjakha kumusomo kumubofu kutsa khulolela nga shiino shinyalikha mubyallo biibibodokhelele imbale. Ne khunyoa, khunaba khuli khu gezesa ingeri isi khunakhola khumumusomo kwesi khulowoza mumaso . Shino shina khuwa kumukisa khufuna injeli infura mu khukhola khumusomo ni khu kakasa khuri khu nyala khu busa kha makhuwa kamalai.

**Nitubasa ndyena mumusomo khuno?**

Nga wakhalilewo khukhutubasa, unanyowa wilemu birebo fitibiri khubona nga unyala wetubasa mumusomo khuno. Shino shinyala shakholebwa nu musalisi mungo mwowo. Nga wabiriremo ate wafukirisile khuba mumsomo khuno, unakhaniwa khu ra ishangumu namwe khuwandikha khu lupapula lwe khufukirisa. Shili isi uli khukalawo khutubasa namwe ta. Nga wakhalilewo khukhutubasa, ushili nilukhosi khururamu usukhanile nga mbawo khumulukhunya ta.

**Nakhila khukola shina nga nitubasile ?**

Nga wafufirisile ukhutubasa, unarebewa birebo biwambakhana khu tsiinda tsindaai ni lwesi ulininalwo sayi, awo nuwo bakhuwe khusomesewa khubiwambakhana ni tsinda (ni khu musomo kwe bumiliyu bwe khamakhono mu khusala) uwebwe I mamakit. Ino ilimu bibindu bye khurambisa ngo sala ni kha sabuni kha fiti.

Shisinza she bakhasi mushyalo banafuna khamasuso khe mukhono nga shitwela mubili mu kit. Kamasuso ke mungono ka kabibwa mu litre ndwela nikhachupa khandi kha 100ML bista khurambisiwa ngo rura mungo. Ibo banafuna kamasuso khano banakharambisa buli bushele mutsingo tsawe. Nga walondele khamagambila khali khu njeli na lina lwesi ulini khurambisa kamasuso khano. Shinamanyisa shili unpangusa kamakhono kowo ni kamasuso chimilundu nga likhumi buli bushele paka lufanyuma lwe kimyesi kitaru nga wamalile khu saala. Ukhwisawo khwe kamasuso khawelemu khunaba mwikangilo lwe Busui HC IV namwe basawo be mubyalo.

Bakhasi be mubyalo babandi banafuna mama kit nenga nga mbamu kamasuso ta.

Nga wafukirisile ukhutubasa mu musomo,khakhobo khe bareberesi khana khubolela khaboye kha mama kit khesi unafuna.

**Nalondelelwa ndyena mu musomo khuno?**

Khunakhurusa kho khamakhuwa kha khakhuwambakho nuyo khunyalise khu khakananatsaka ni nawe. Shino shina kholekhewa nga khubirira khu simu namwe basawo be mwikangilo mubwalo bwenywe .Khukanikha nayo shili she kamakhulu lwekhuba shikhuyeta khumanya esi usalele niwo khunyale khukhu nyola awo khunyalise khulolelela bulamu bwo mwana wowo.

Nga wamalile khusala, uli ni khubolela kho umusawo wowo uwe mushalo. Anamanyisa khakhobo khe bareberesi banesta khukhurusa kho khamakhuwa khawambakhana ni khutsala khwo mwana wowo. Shino shina kholikha khumulundu khunyowa ngo bona umusawo nga

wama lile khusala, shitela khubawo mu sawa abili munya tsinyowa. Kimilundu ichilakho che khubona umusawu nga wamalile khu sala chili ni khuba mwikangilo lwe akhwimbi nga bulijo mu saabiti ndwala paka tsibili ni sabiti mukaga nga wamalile khutsala. Kumusomo khuna kama nga umwana wowo olisile kimyesi kitaru. Khakhobo khe bareberesi khana, kakamise kumusomo mubutongole nga bakwishenga iwe nu mwana wowo mungo mwo.

Umusawo we mushalo anishenga umwana wowo khuwentselesa ishokesanisakho bulwale buli isi mukhananila nashindi uli ni khu bolela umusawo buli isi umwana wowo alwalila. Umwana wowo anishengebwa mungeli yene ndwela buli isi umuyilia mwikangilo namwe imbale mwikangilo likhulu.

Nga umwana wowo okesele kho bulwale bwosi, una bolewa khutsa mwikangilo ilitubasa mu musomo lilishimbi ate uli ni umanyisa bareberesi be kumusomo kuno isawa yeneyo.

- Basawu khwikhangilo mushalo bani shenga umwana wowo nuwo bakwise mwikangilo likhulu imbale ukhwiyongela nu khwimenyelesa khufurakho nga bulwale bwekamani.
- Babana babokesanisa bari balimu bulwale bana janjabiwa khubusa mu ward ye babana mwikangilo likhulu imbale.

Nga umwana wowo akhwolesanisa kho bulwale bwosi,anarama mungo ninawe akhali nga aliwo ukhimulugunya khwositsana. Langa buyeti nga imbela ye bulamu bwo mwana wowo yonikhile namwe nga etsilekho buwangafu bwotsina.

**Nakhila khukhuambilamo?**

Tawe. Shiili isi uli khukhalawo utubasa mumusomo. Khukhutubasa khwekwikanisa busa ate uli uwelukosi khukururamo mumbuka isi ukanisila ate shi shonegisa bukangi bowo bubulakho ta.

**Shiina shiili mu masuso khe mungono khalimu indali?**

Kamasunso khe mungono khalimu indali (Alsoft V) kakholikhila ikakira Uganda ni Saraya East Africa Co.Ltd. ilimo kamasunso khekhukhwara. Kano kera buwukha bufura. Kalimo bilungo biibindi isi bagatakho nuwo bukiriwa bwafukirisiwa khurambisiwa.

**Bunendeshiina bubuli khukhuambilomo?**

Babandu bafurisa bukhali banyala khurambisa kamasunso gekhaukha mungono nga mbawo

Buwangafu bwostisana ta. Ne nga wabele ni likonzo khumukhono khanyala khulumakirisa nga warambisire imbuka ichindi kanyala kalela shikhoba khubesema namwe shakala. Nga shiino shya kholikhile yakamisilawo, ubolele bareberesi.

Kamasunso khalimu indali kakaniwa ni khuwambikha ni bubunende. Lwekhuba mulimo shikha she indali inyala yamba wangu kumulilo nalundi ili ni shikha she butwa kale wakha wabikha isi babana namwe bisolo bikhola ta.

**Iliwo bilayi niambilomo?**

Kamasunso khalimu indali kano karambisikha ni bakangi mumakingilo khale khumilisa kamakhono ni mubyalo. Kamasunso khalimu indali mumusomo kuno gali khulinda kamakhono gowo kamamiliu ni umwana wowo nuwo gakhendese bulwale. Barambise be kamasunso khalimu indali kano bakana naabi khurambisa khumakhono khawe.

**Nenga shyabellewo shiangafu?**

Nga wabelekho ni shirebo shiambakhana ni kumusomo khuno, nyoa ukhanikhe isi bareberesi bagane bakhakekho khukhulomo birebo bwoyo. Nga wakhusenyukha ni isambo isi bililemo birebo nashirio ugana khukhumulungunya. unyala wareba James Ditai Nashitsolongo uwa Sanyu Africa Research Institute. Khu simu 0711620193.

Nga shindu shyakholikhile shikhali shilayi ta ate walumisiwile mumbuka iye kumusomo kuno ate shiino shyakholikhile lwekhuba umundu shiwenanilikhilekho ta. Awo unyala wa habila Univasiti iye Liver pool kumusango mumakayilo khe mu UK. Mubela nga nzo wamwene wusesula biibikaniwa bwositsana.

**Khukhutubasa khose mumusomo kuno gwe shishyama?**

Eee. khane khulondelele isambo itayi ni kamagambila nuwo kamakhuwa kakhuwambokho kabe mushyama. Kamakhuwa kakhuwamkho ni lisiina khane bilindikhiwile ilala mushyama ate khane bifugirisikhe khubonekhikho ni bareberesi namwe (bebunyala khubona bari kumusomo kuli khutsa bulayi) kamakhuwa khauambokho nga kakabanasiwele khufurawo nga lisiina lyowo Lilikho ganikhurusekho ate mbawo unakhumenya ta.

**Shina shinakholekha khu binama mu musomo khuno?**

Binama mumusomo kuno binarambisiwa khukhalawo tsingeri tsifurisa khutimisa khumusomo khumuhulu kutsa khubawo imbale mu Uganda.

**Nanu ulihurengeharengeha ni huramu liwumule mu musomo khuno?**

Kumusomo kuli khutimisiwa ni Univasiti iye Liverpool mu UK ni SAFRI Imbale. Medical Research Council iye mu UK niye ili khuramu kamawumule.

**Naanu uwemenyelesa kumusomo kuno?**

Kumusomo khunemwenyelesiwa ate khufugirisiwe ni Univasiti iye Liverpool ni khakhobo khakhakhulila bye tsisambo mu bye khusoma mwikangilo likhulu Imbale. Ne bukhobo bubuwule bubiili buno bubona buri kyimitendela kyili kyimilayi nalundi shikyila bulamu bwenywe khubunende byosi ta.

**Wanyala khuyila imbuka khulolela ni khuwulilitsa kamakhuwa kano.**

**Nga abe ukhana khukanikha isi umundu uli khukhakhobo khe bareberesi unyala wabona 0782982040, umuyeti wo mureberesi 0782614677**

**Baby Gel:** **Kumusomo khunyowa mukhugesesa kwe khuwa bamayi basalire kamasuso khalimo indali khuhendesa bulwale mubana bafiti mungo**

**LUPAPULA LWE KHUFUKIRISANA**

*Nga wasenyukhile ni biromo biino bibirokho kabana mu bu box umale urekho ishangumu ni tsinakhu, asi welupapula*

Nasomile, (basomile) nafunile kamakhuwa gali mu lupapula lye kamakhuwa kebalwale

(version 2.0, 04^th^ April, 2015) ate narebere nibirebo byosi byesi nganile.

Namanyile indi khuli khukhwikanisa khwose busa khukhwiambilamo namwe inyala nalula

Mumusomo nga na khuwayo ilomo yosi ta.

Nfukilisa bareberesi khufuna khamakhuwa kha mambakho ukhwama mubitabu bwe likangilo namwemu mu bebulebe ni basale nga bakhunyala ukhu nyola semwine khumala imbukha iye tsi sabiti tsibili ukhwama khutsinakhu tsesi banoma khukobola.

Namanyile ini kamakhuwa khesi bandusirekho mumusomo kuno kanyala kabonekha ni

babandu khu khwama mu bitongole bilolele kumusomo kuno nuwo babone bari

Kali khubusiwa bulayi.

Nga kusomo gwa wele, kamakhuwa gase, ganyala kabikhiwa, khubwimi bya intaneti bareberesi babandi

barambise. Khe mutambemu kamasiina khase ni kamakhuwa ganyala kagila bantegela.

Indi umusanyufu lwe kamakhuwa khase khurambisiwa musambo iyi.

Ifugirisa khu khutubasa musomo kuno

__________________________ ___________________ __________________

Lisiina lyo umwitubasi Lunakhu Shagumu

__________________________ ___________________ __________________

Lisiina lyo mundu ubelewo (nga shisobokha) Lunakhu Shagumu

__________________________ ___________________ __________________

Lisiina lyo mureberesi Lunakhu Shagumu
