# Supplementary material for: Optimising informed consent for participants in a randomised controlled trial in rural Uganda: a comparative prospective cohort mixed-methods study
Source: Trials. 2018 Dec 22;19:699. doi: 10.1186/s13063-018-3030-8 (PMC6304001; doi:10.1186/s13063-018-3030-8)
Supplement: Supplementary file 1 — The approved participant information sheet (zipped PIS english and Lumasaba). (ZIP 48 kb) [file 13063_2018_3030_MOESM1_ESM.zip › PIS_English.docx]

**BabyGel Pilot: a pilot study of a cluster randomised trial of the provision of alcohol handgel to postpartum mothers to prevent neonatal infective morbidity in the home**

**Protocol version 2.0 4^th^ April 2015**

**PARTICIPANT INFORMATION SHEET**

**Invitation**

You are invited to take part in this study. Before deciding that you want to participate, it’s important for you to understand why it is being conducted and what will be involved. Please listen to the following information carefully. You can discuss it with your family or friends if you wish. You will be **given a copy of this information sheet** to keep. In case you need more information or you have any more questions, please ask the BabyGel research team. Take your time to decide whether you want to take part in the study – it is completely up to you!

**What is this study all about?**

When your baby is very young, it is at high risk of infection. The germs that cause the infection can come from the birth canal during delivery, or picked up from other people in the hospital, health centre or at home. When an infection is detected, it can usually be treated with antibiotics. But sometimes infections are so severe that antibiotics cannot help. The best way of preventing infection is to stop the germs getting onto your baby to start with.

Germs are usually carried on the hands of those who look after the baby. That is why it is important to keep your hands very clean when looking after a baby. If you do not, then you can carry germs to the baby from other people, from dirt or from animals and cause infection. The best way to keep your hands clean is to wash them carefully with soap and water. But this is not so easy when there is no water tap nearby, or when the water itself is dirty. This is why we are testing whether the regular use of a special antiseptic hand rub can prevent infection in the newborn. We are planning a large study looking at how effective this is in the villages around Mbale. But first, we will be testing the design of the proposed large study. This will give us an opportunity to get the study design right and to prove that we can collect good quality data.

**How can you take part in the study?**

If you choose to participate, you will first need to answer some questions to see if you can take part. This can be done by a research midwife at your home. If you are eligible and choose to participate, you will be asked to sign a consent form. It is up to you to decide whether or not to take part. If you decide to take part you are still free to withdraw at any time and without giving a reason.

**What will you need to do if you take part?**

If you agree to participate, you will be asked questions about your previous pregnancies and current pregnancy. Then you will receive usual antenatal education (including teaching on the importance of hand hygiene for preventing maternal and neonatal morbidity) and be given a MamaKit. This contains equipment for use at the time of delivery as well as a small bar of soap.

Women in half of the villages will receive alcohol-based hand rub as part this kit. The hand rub is supplied in a 1-litre bottle with an extra 100ml bottle to be used when travelling away from home. Those who receive the handrub will use it regularly at home, following our instructions on how and when to use it. Usually it will mean cleaning your hands with the solution at least 10 times each day until three (03) months after childbirth. Replacement supplies of handrubs will be available from either the village health workers or the pharmacy at the Busiu HCIV.

Women in the other villages will still receive the MamaKit but without the hand rub.

If you agree to be in the study, the research team will then inform you which pack you will receive.

**How will you be followed up in the study?**

We will take contact details from you so that we can keep in touch with you. This may be by telephone or through the village health worker in your village. Keeping in contact is important so that we know when you deliver and can find you afterwards to monitor your baby’s health.

Once you give birth, you should notify your village health worker. He / she will then notify the research team who will visit you to collect data about the birth and your baby. This will be done at the first postnatal visit, ideally within the first 24 hours. Further routine postnatal checks should be done in the nearest community health facility as usual at 1-2 weeks and 6 weeks after birth. The study will end when your baby is 3 months old when the research team will do a formal end-of-study assessment of you and your baby at your home.

Your VHW will screen your baby for signs of infection at every contact with you and your baby, and you should also notify the VHW if your baby becomes ill. Your baby will also be checked using the same criteria if you present at the local health centre or Mbale Hospital.

If your baby shows signs of infection, then you will be referred to the nearest participating health facility and you should notify the research team immediately.

- - Health workers at the community health centre will assess the baby and refer to Mbale hospital for further management if it has serious illnesses.
  - Infants in whom there are signs of disease will receive free care in the Paediatric Department of Mbale regional referral hospital.

If your baby has no sign of infection, then s/he will remain with you at home unless there are other concerns. You should seek care again if your baby gets worse or develops any new problems.

**Do you have to take part?**

No. It is up to you to decide whether to join the study. Participation is voluntary and you are free to withdraw at any time without it affecting any of your subsequent care.

**What is in the antiseptic handrub solution?**

The handrub solution (Alsoft V) is one that is made in Kakira, Uganda by Saraya East Africa Co., Ltd. It contains surgical ethanol - this kills most germs. It also has other ingredients added to make it more acceptable to use.

**What are the possible risks of taking part?**

Most people can use the handrub without any problems at all. However if you have a cut or graze on your hand, then it may sting when you use the hand rub. Occasionally, people get a reaction to the handrub and their skin can become red or itchy. If this happens then you should stop using it immediately and notify the research team.

The handrub also needs to be handled with care. Because it contains a form of alcohol, it can set alight easily and so should be kept away from flames and sparks. It is also a weak poison and so should be kept out of reach of children or animals.

**Are there any benefits to taking part?**

This handrub is already commonly used for hand cleaning by doctors and nurses in hospitals and in the community. Use of the handrub for this study should therefore keep your hands very clean and may reduce infections in both you and your baby. Many users also like the feel of the handrub on their hands.

**What if there is a problem?**

If you have a concern about any part of this study you should speak first to the researchers who will do their best to answer your questions. If you are still unhappy and wish to complain, you can do this via James Ditai, Director of the Sanyu Africa Research Institute (telephone 0711 620 193).

If something goes wrong and you are harmed during the research, and this is due to someone’s mistake, then you may be able to take legal action for compensation against the University of Liverpool in the UK. In such cases you may have to pay your legal costs.

**Will your taking part in the study be kept confidential?**

Yes. We will follow best ethical and legal practice and all information about you will be handled in confidence. All personal information with your name attached to it will be kept strictly confidential and will only be looked at by members of the research team (or by authorities checking that the trial is being conducted correctly). Any information about you that is shared beyond that will have your name removed so that others will not be able to link it with you personally.

**What will happen with the results of the study?**

The results of this study will be used to decide the best ways of conducting the proposed main study in Mbale Uganda.

**Who is organising and funding the research**

The research is being run by the University of Liverpool in the UK and the Sanyu Africa Research Institute in Mbale. The study is funded by the Medical Research Council in the UK.

**Who has reviewed the study?**

This study has been examined and approved by the University of Liverpool and the Mbale Regional Hospital Research Ethics Committees. These two independent committees make sure that it is of high quality and does not put you at any risk.

**Thank you for taking the time to read, or listen to me, or watch and listen to the video.**

**If you would like to speak to a member of the research team please contact 0782982040, Research Assistant on 0782614677**
