# Supplementary material for: Optimising informed consent for participants in a randomised controlled trial in rural Uganda: a comparative prospective cohort mixed-methods study
Source: Trials. 2018 Dec 22;19:699. doi: 10.1186/s13063-018-3030-8 (PMC6304001; doi:10.1186/s13063-018-3030-8)
Supplement: Supplementary file 2 — The approved slides (English version and Lumasaba version). (ZIP 12399 kb) [file 13063_2018_3030_MOESM2_ESM.zip › BabyGel English PIS Slide v8R2.pptx]

## Slide 1
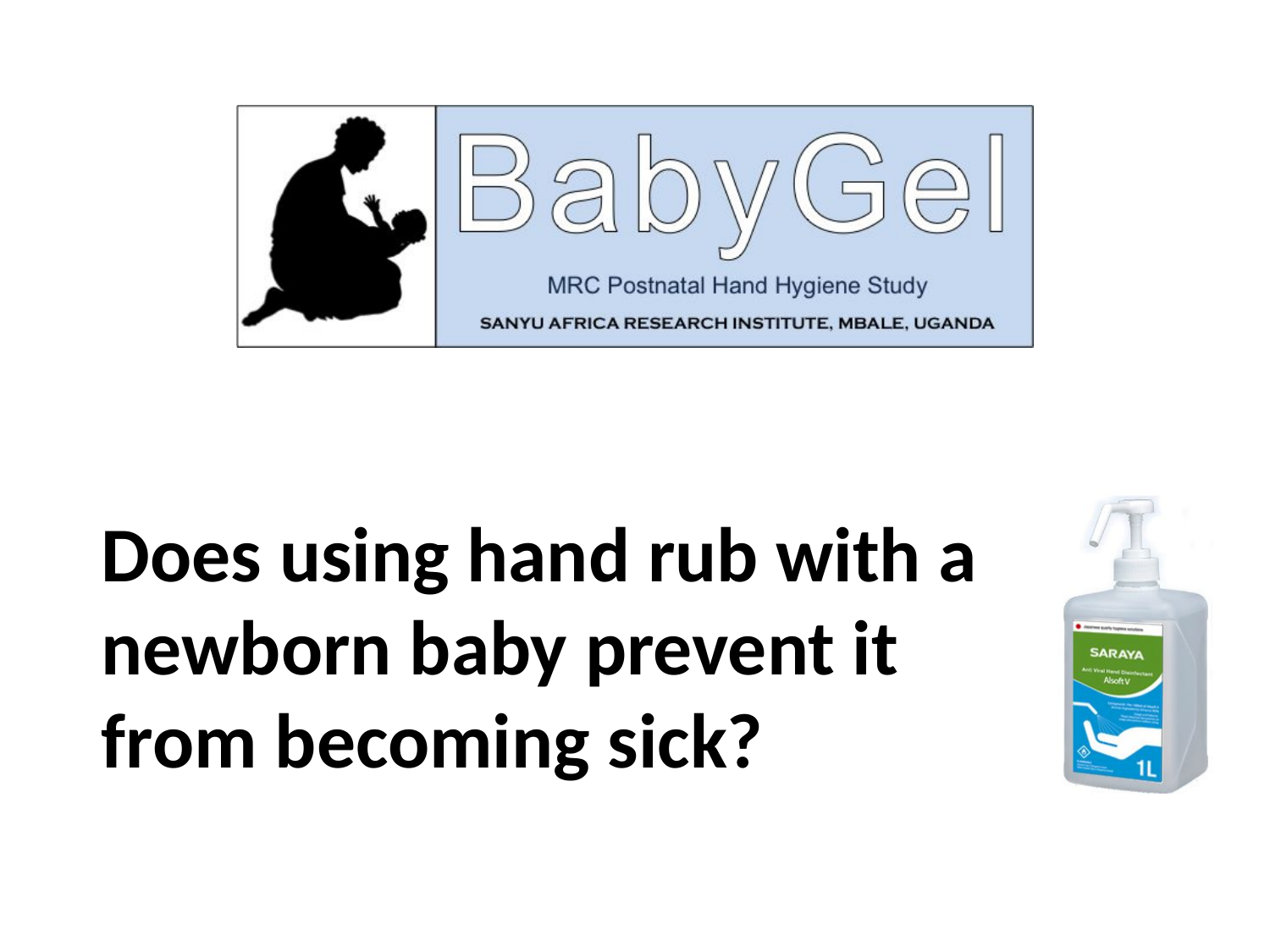

# Does using hand rub with a newborn baby prevent it from becoming sick?

## Slide 2
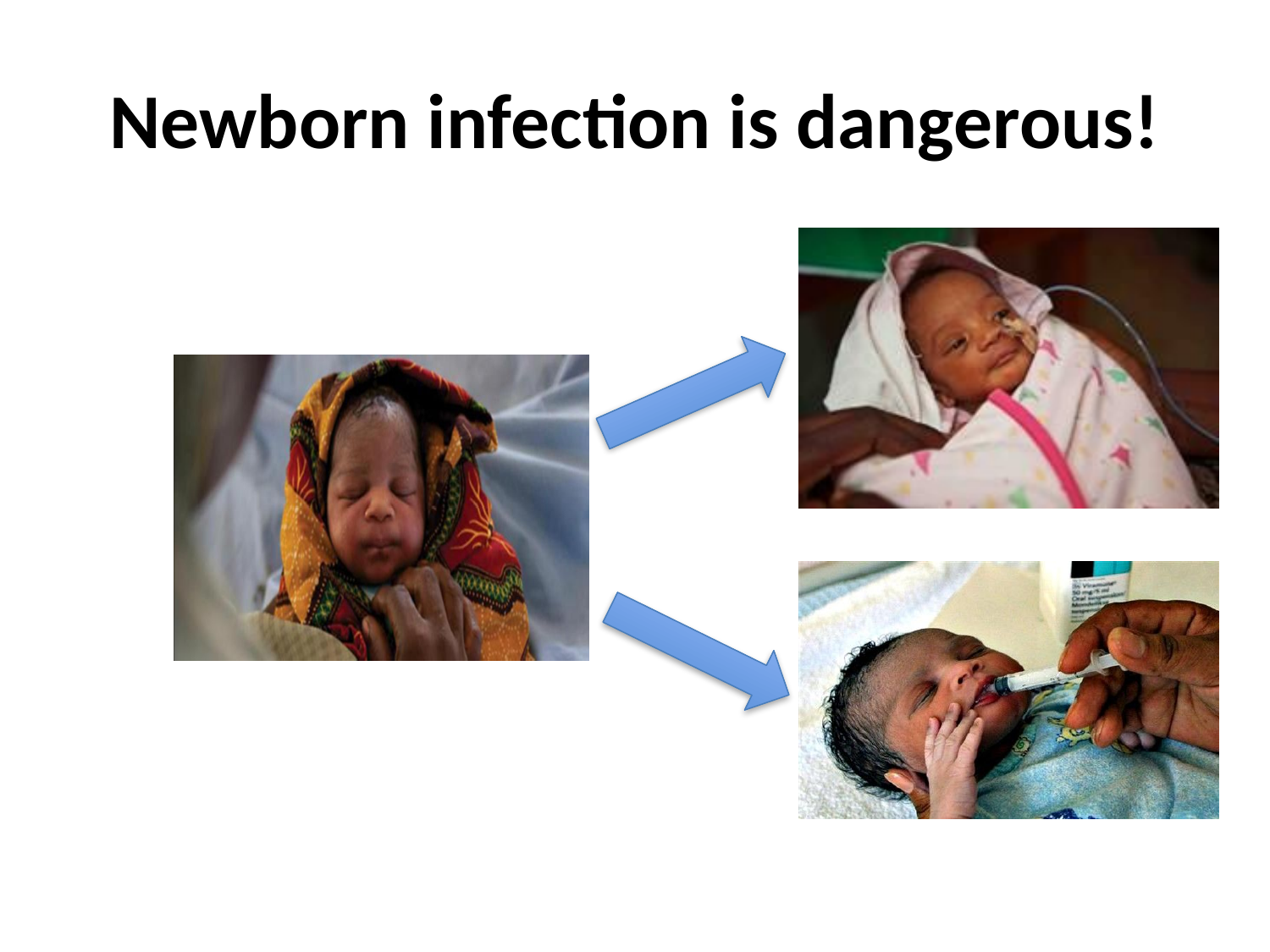

# Newborn infection is dangerous!

## Slide 3
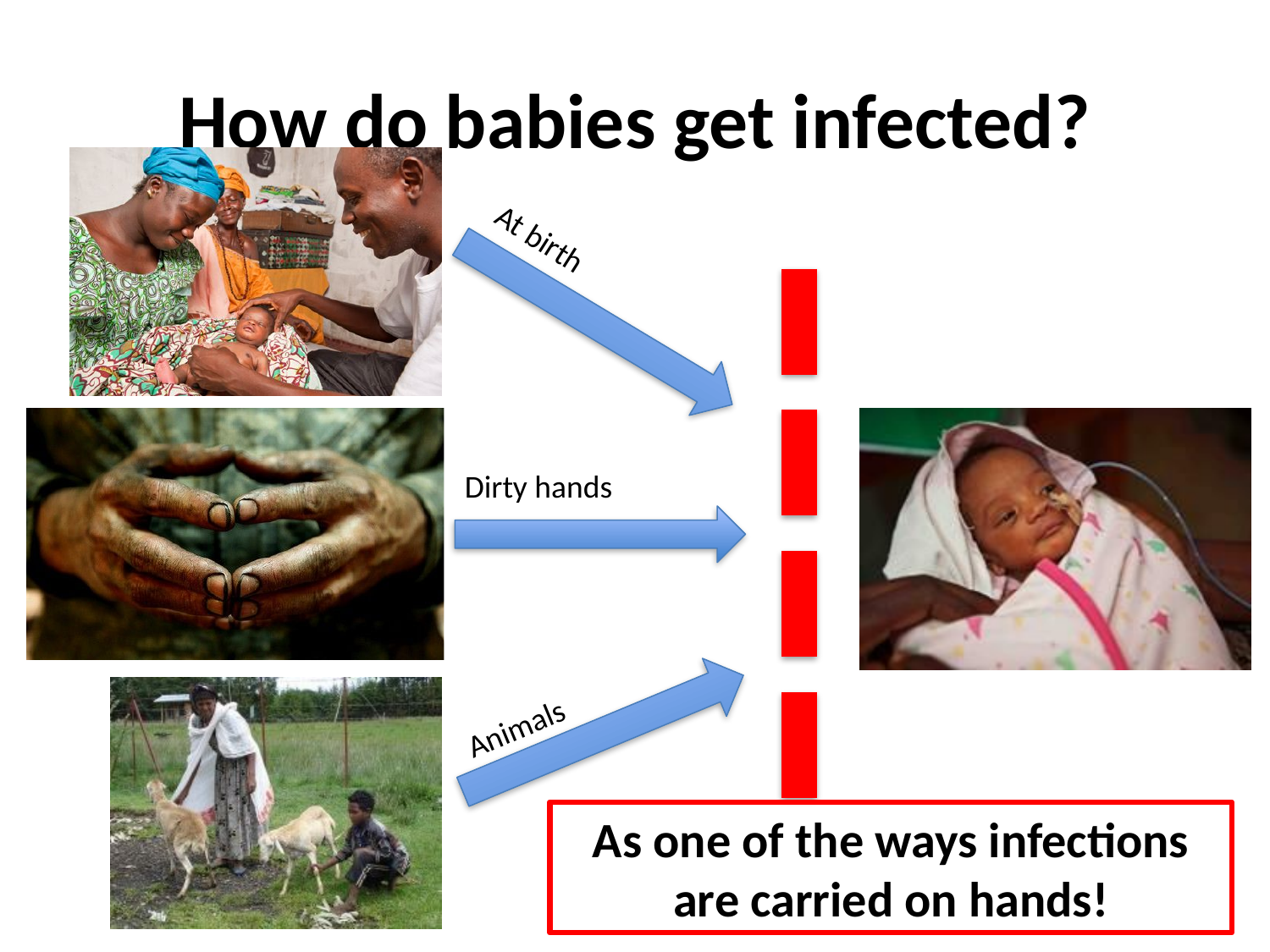

# How do babies get infected?
At birth
As one of the ways infections are carried on hands!
Dirty hands
Animals

## Slide 4
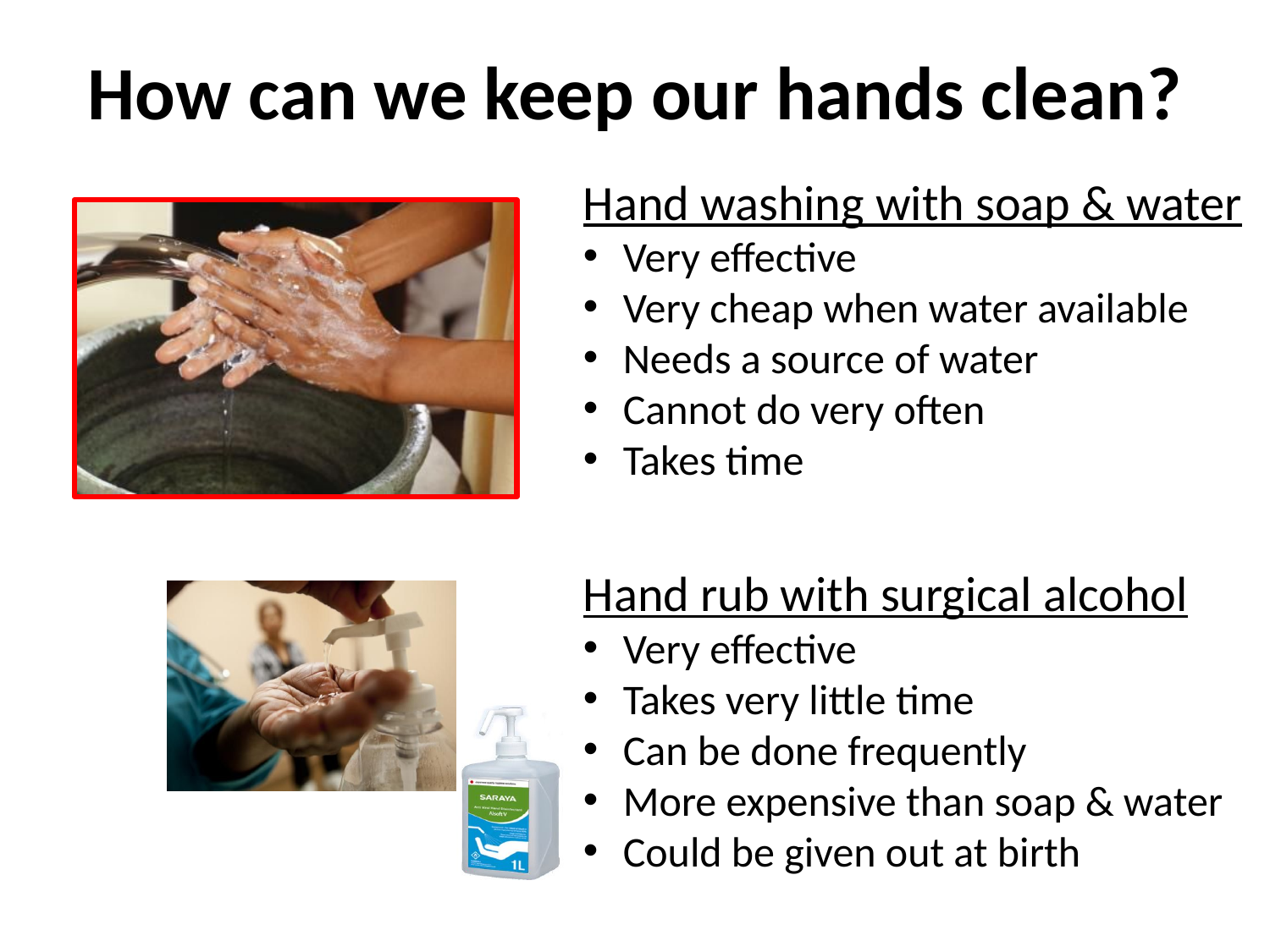

How can we keep our hands clean?
Hand washing with soap & water
Very effective
Very cheap when water available
Needs a source of water
Cannot do very often
Takes time
Hand rub with surgical alcohol
Very effective
Takes very little time
Can be done frequently
More expensive than soap & water
Could be given out at birth

## Slide 5
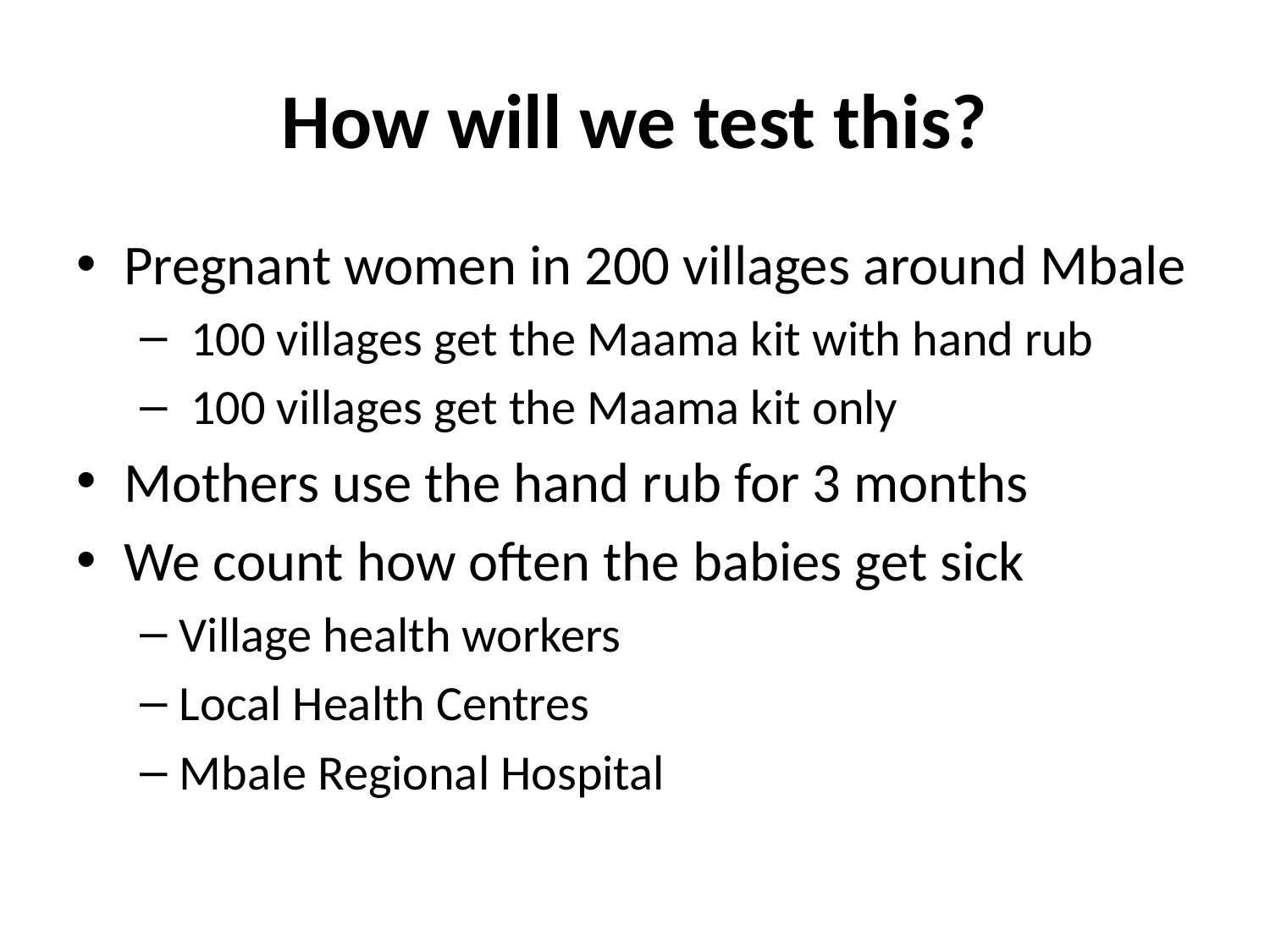

# How will we test this?
Pregnant women in 200 villages around Mbale
 100 villages get the Maama kit with hand rub
 100 villages get the Maama kit only
Mothers use the hand rub for 3 months
We count how often the babies get sick
Village health workers
Local Health Centres
Mbale Regional Hospital

## Slide 6
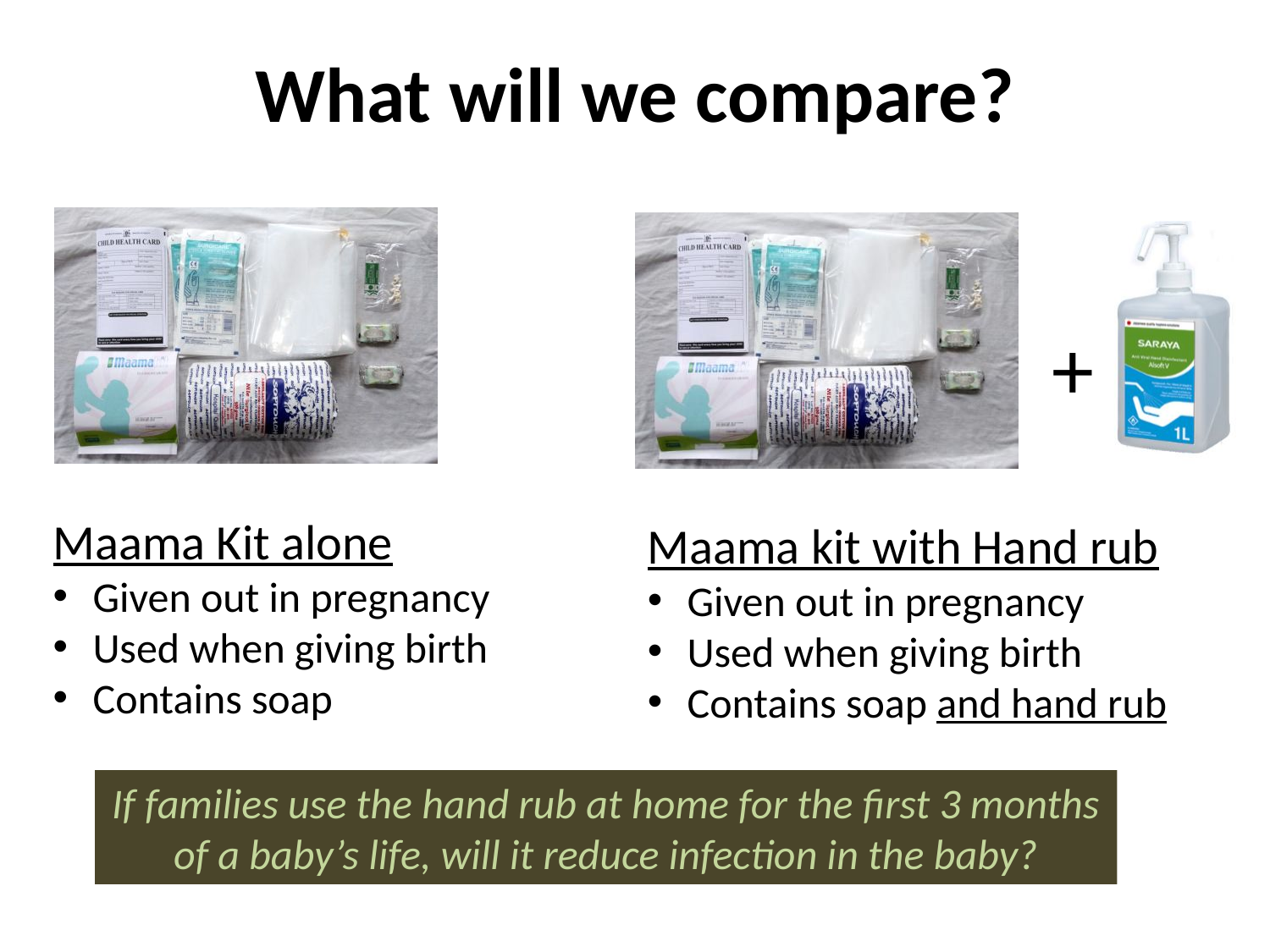

What will we compare?
+
Maama Kit alone
Given out in pregnancy
Used when giving birth
Contains soap
Maama kit with Hand rub
Given out in pregnancy
Used when giving birth
Contains soap and hand rub
If families use the hand rub at home for the first 3 months of a baby’s life, will it reduce infection in the baby?

## Slide 7
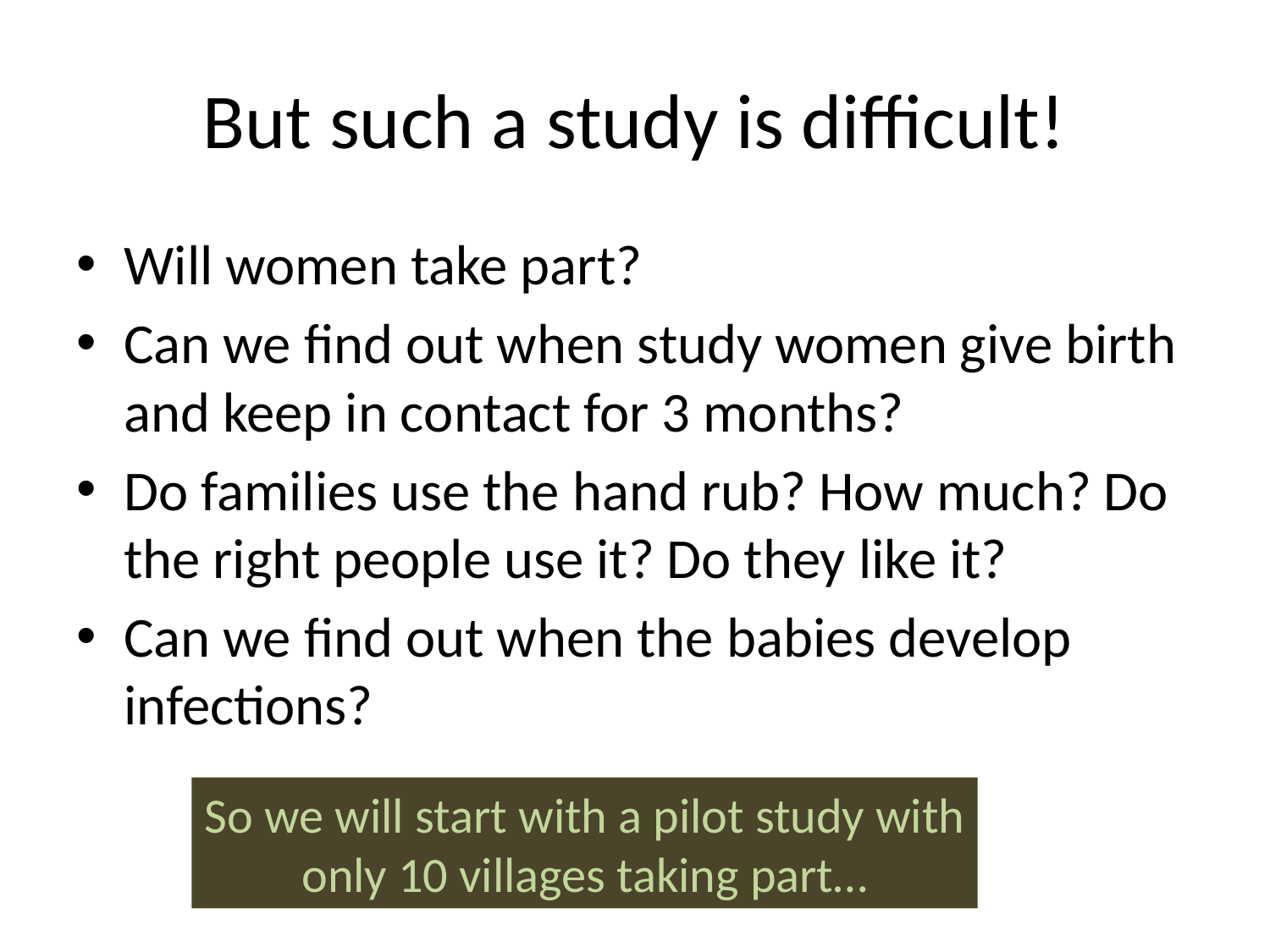

# But such a study is difficult!
Will women take part?
Can we find out when study women give birth and keep in contact for 3 months?
Do families use the hand rub? How much? Do the right people use it? Do they like it?
Can we find out when the babies develop infections?
So we will start with a pilot study with only 10 villages taking part…

## Slide 8
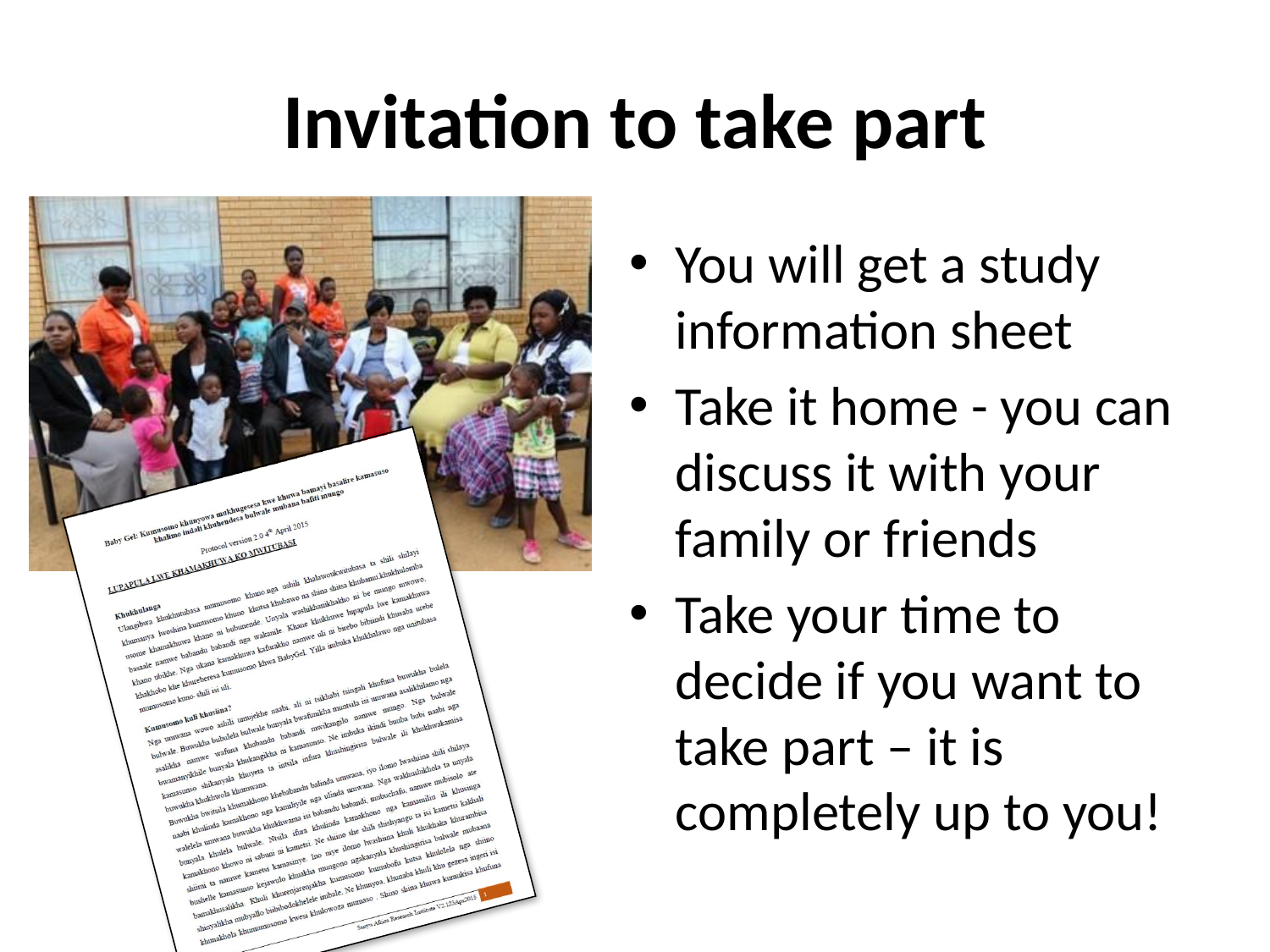

# Invitation to take part
You will get a study information sheet
Take it home - you can discuss it with your family or friends
Take your time to decide if you want to take part – it is completely up to you!

## Slide 9
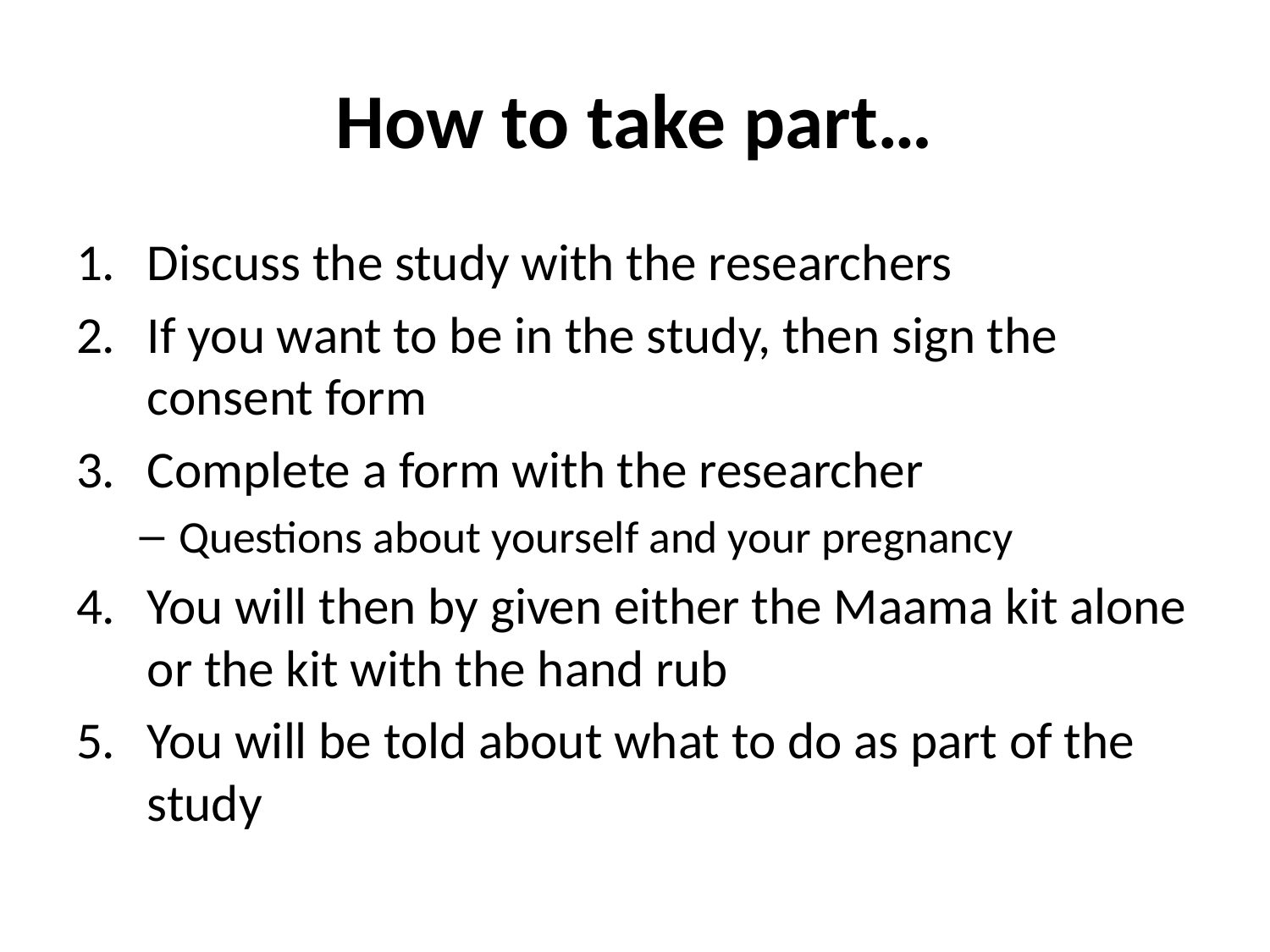

# How to take part…
Discuss the study with the researchers
If you want to be in the study, then sign the consent form
Complete a form with the researcher
Questions about yourself and your pregnancy
You will then by given either the Maama kit alone or the kit with the hand rub
You will be told about what to do as part of the study

## Slide 10
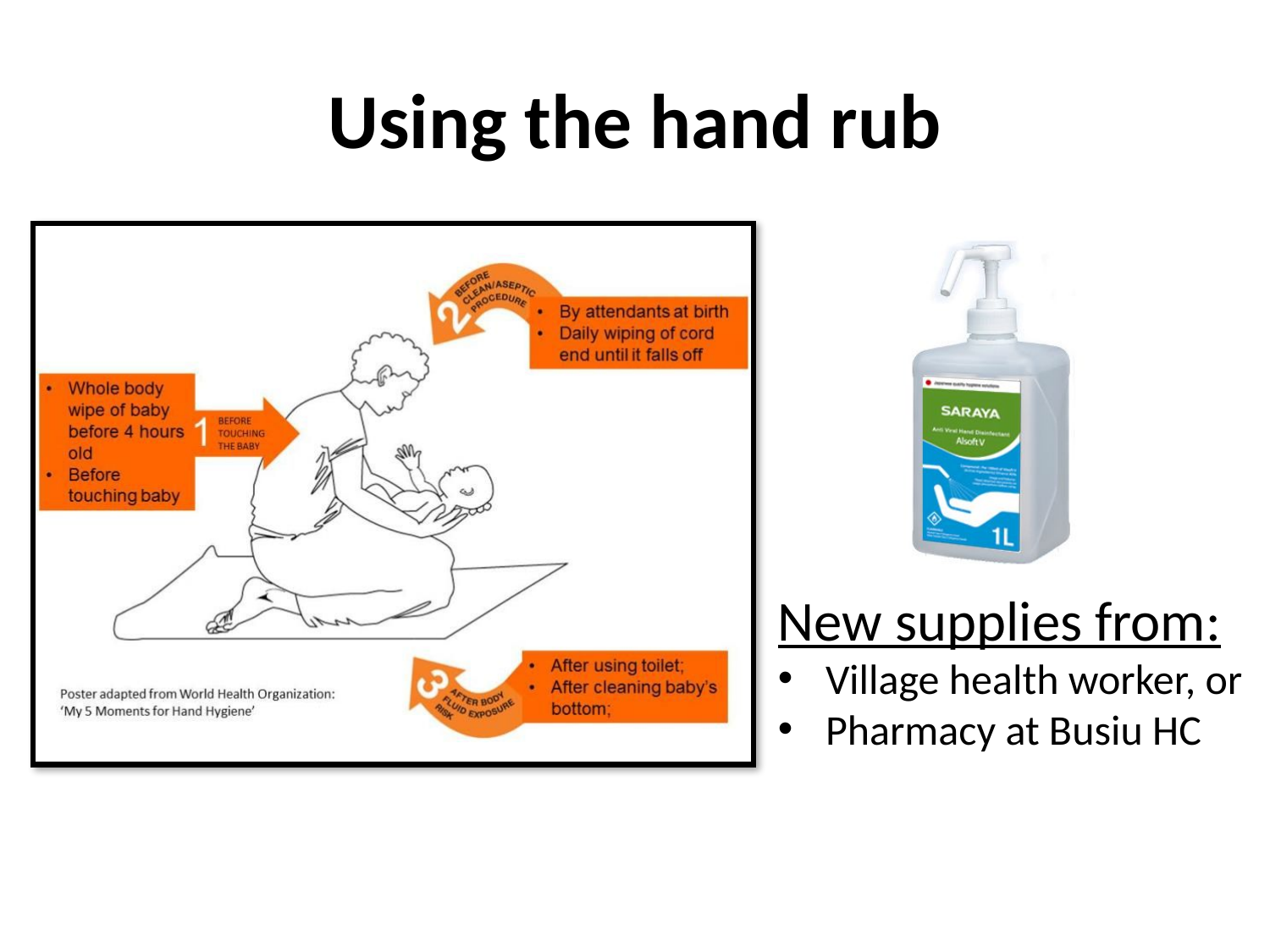

# Using the hand rub
New supplies from:
Village health worker, or
Pharmacy at Busiu HC

## Slide 11
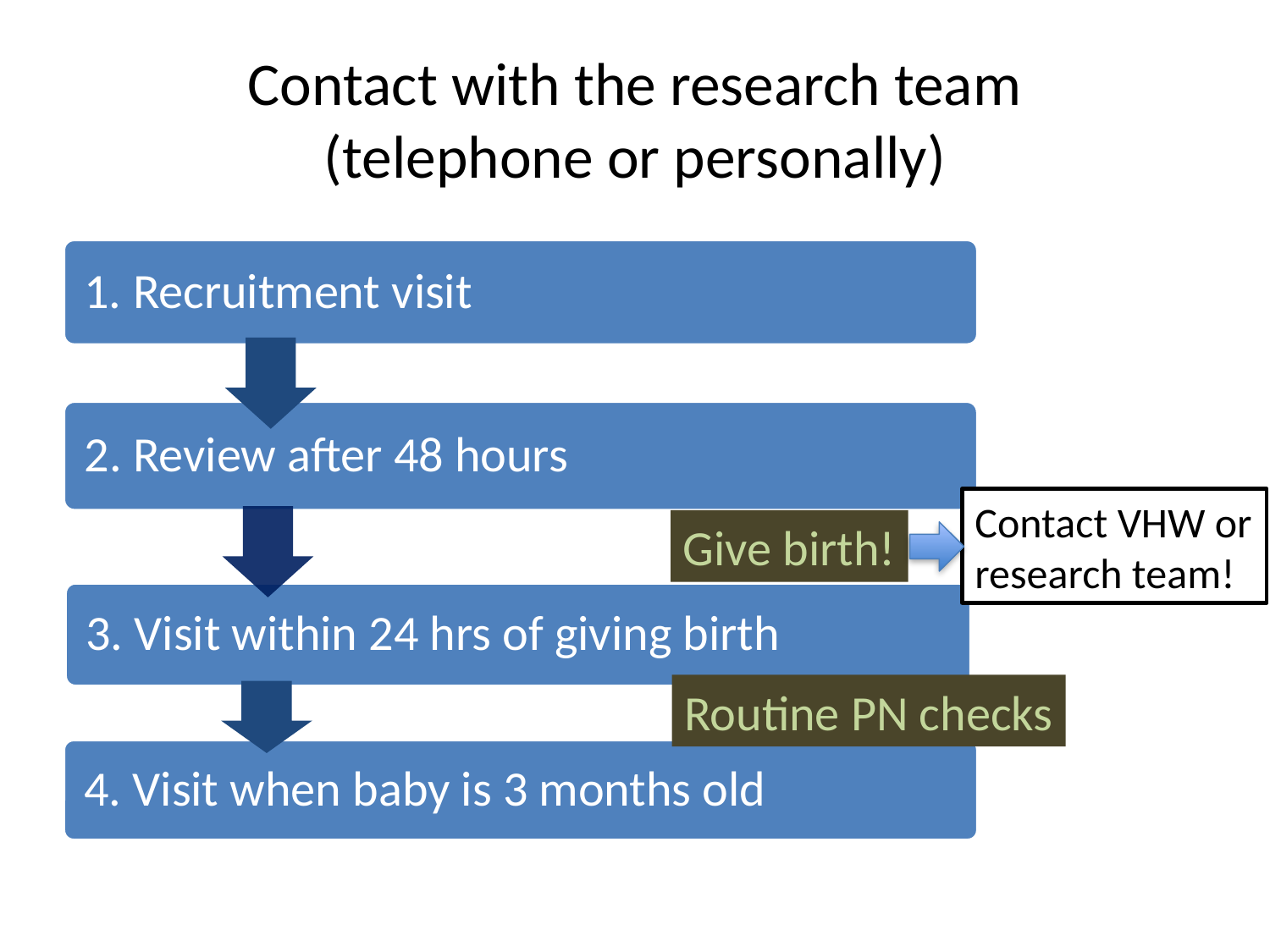

# Contact with the research team (telephone or personally)
Contact VHW or research team!
Give birth!
Routine PN checks

## Slide 12
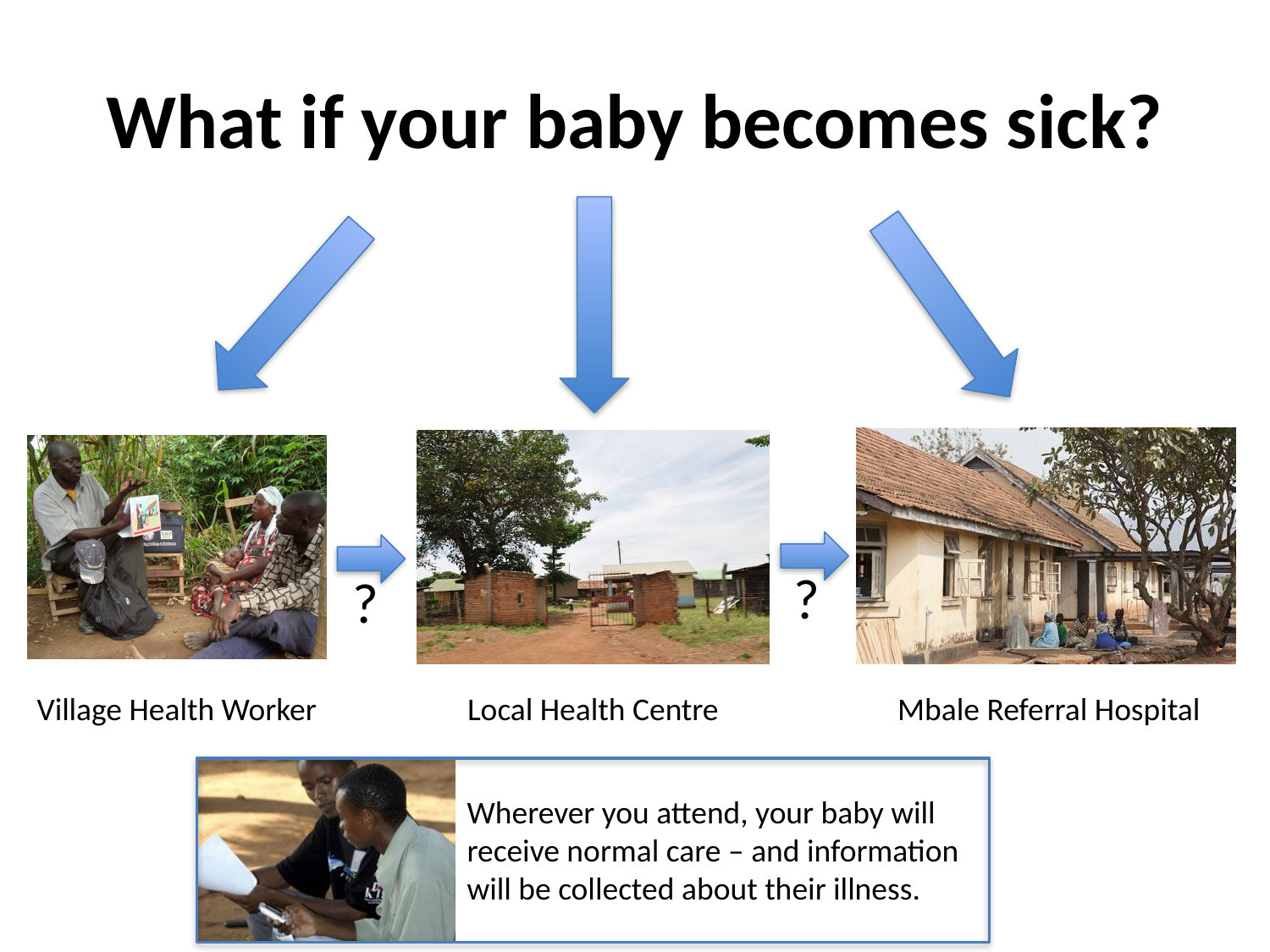

# What if your baby becomes sick?
Local Health Centre
Mbale Referral Hospital
Village Health Worker
?
?
Wherever you attend, your baby will receive normal care – and information will be collected about their illness.

## Slide 13
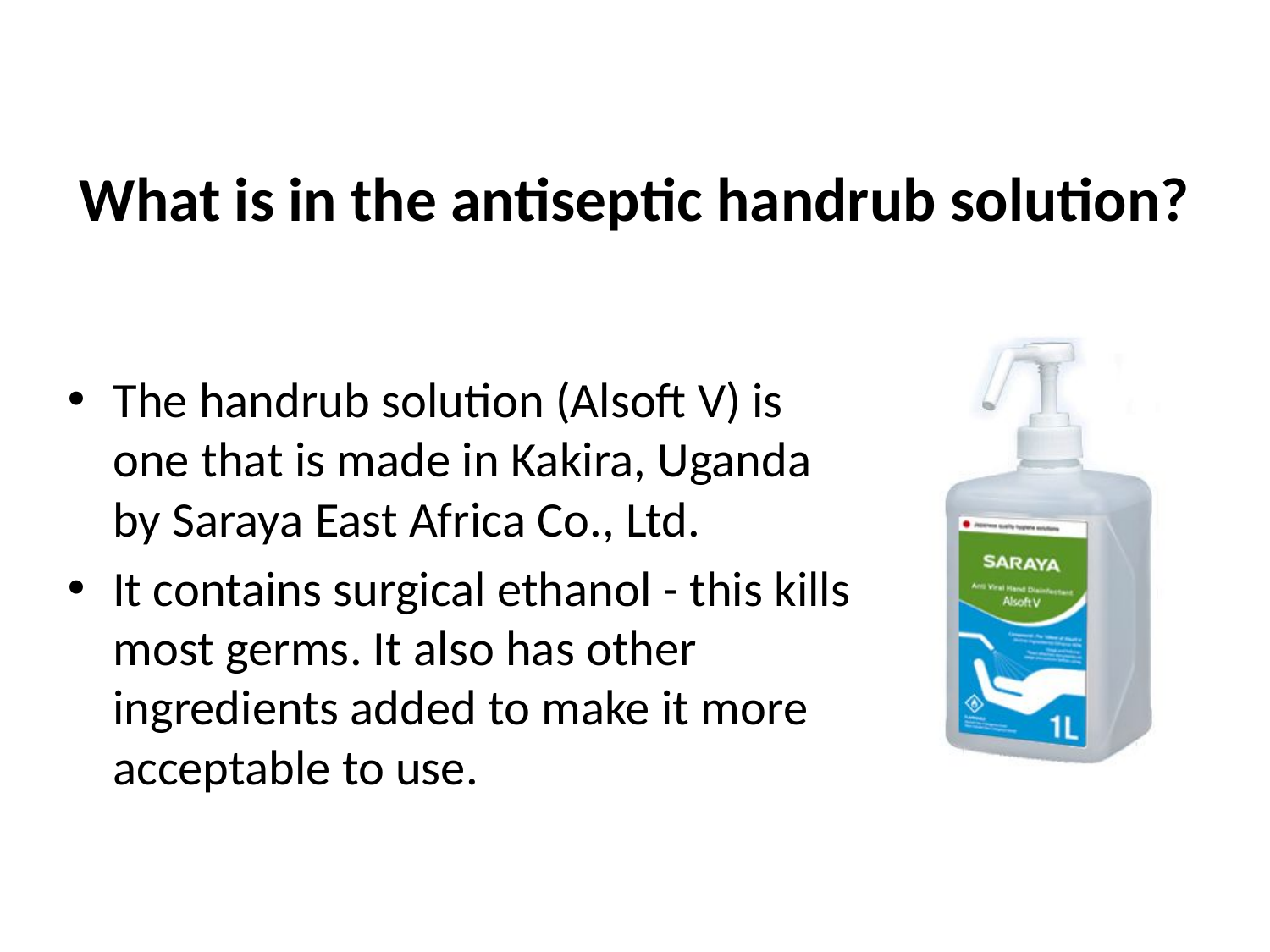

# What is in the antiseptic handrub solution?
The handrub solution (Alsoft V) is one that is made in Kakira, Uganda by Saraya East Africa Co., Ltd.
It contains surgical ethanol - this kills most germs. It also has other ingredients added to make it more acceptable to use.

## Slide 14
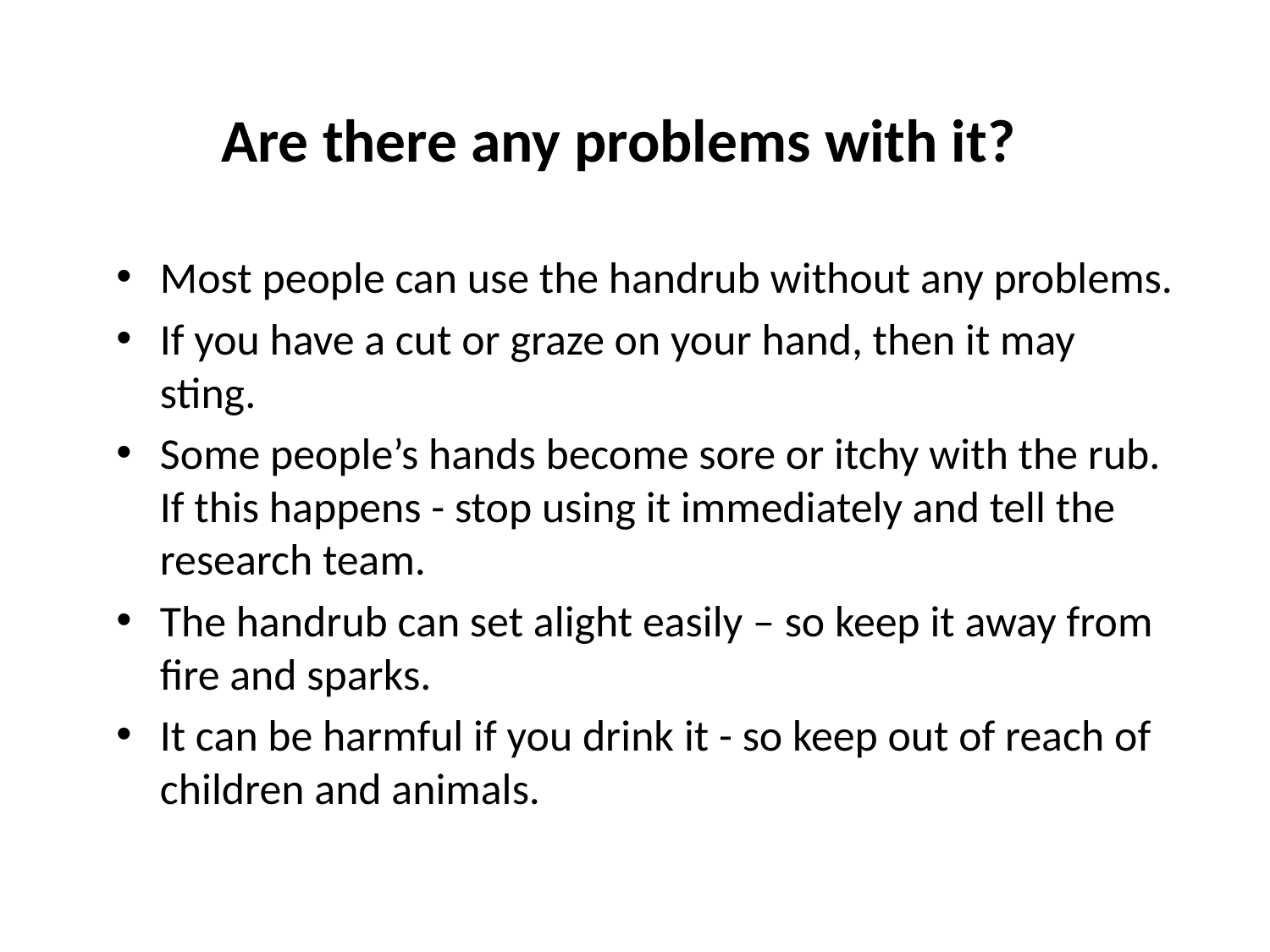

# Are there any problems with it?
Most people can use the handrub without any problems.
If you have a cut or graze on your hand, then it may sting.
Some people’s hands become sore or itchy with the rub. If this happens - stop using it immediately and tell the research team.
The handrub can set alight easily – so keep it away from fire and sparks.
It can be harmful if you drink it - so keep out of reach of children and animals.

## Slide 15
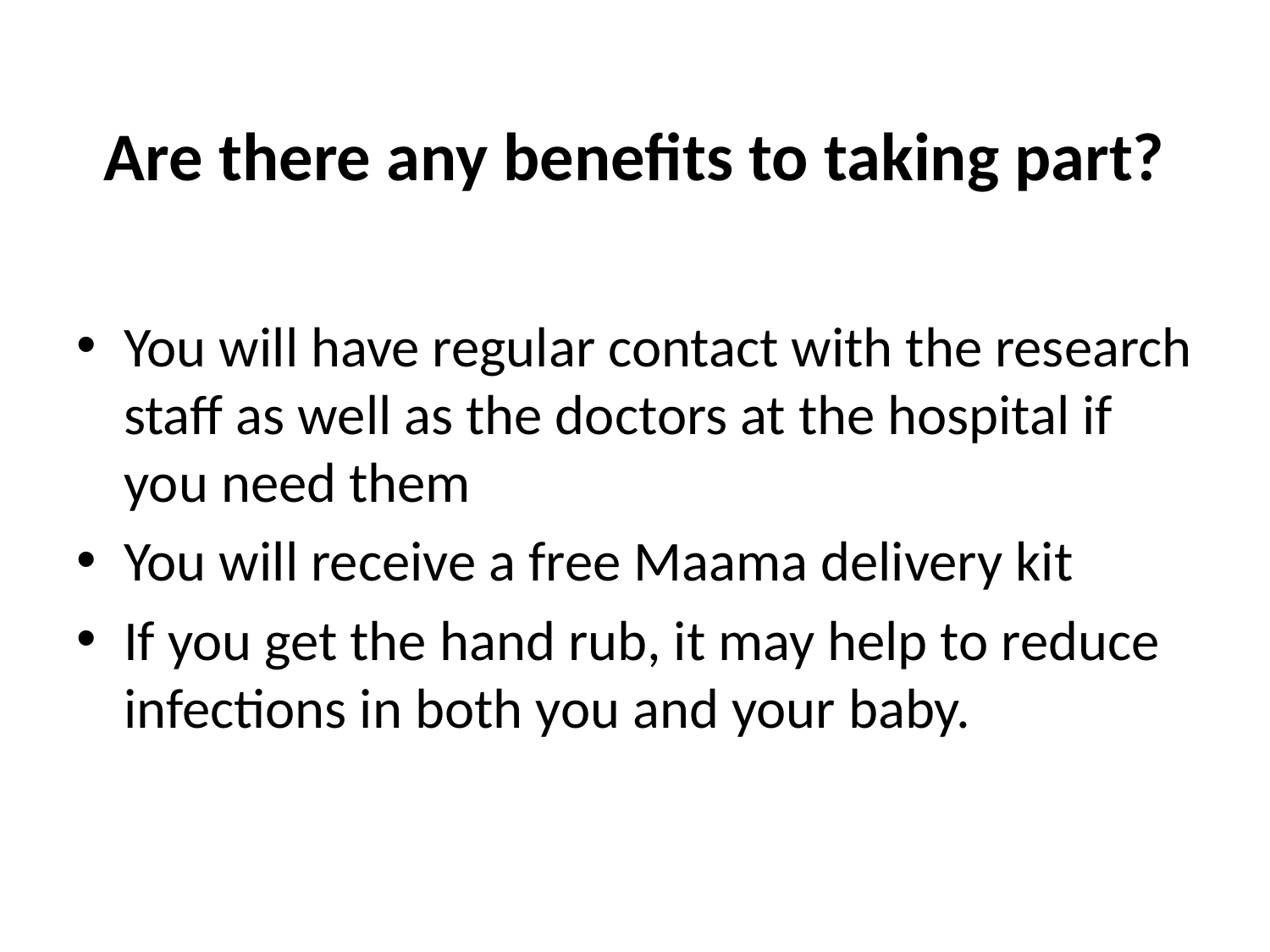

# Are there any benefits to taking part?
You will have regular contact with the research staff as well as the doctors at the hospital if you need them
You will receive a free Maama delivery kit
If you get the hand rub, it may help to reduce infections in both you and your baby.

## Slide 16
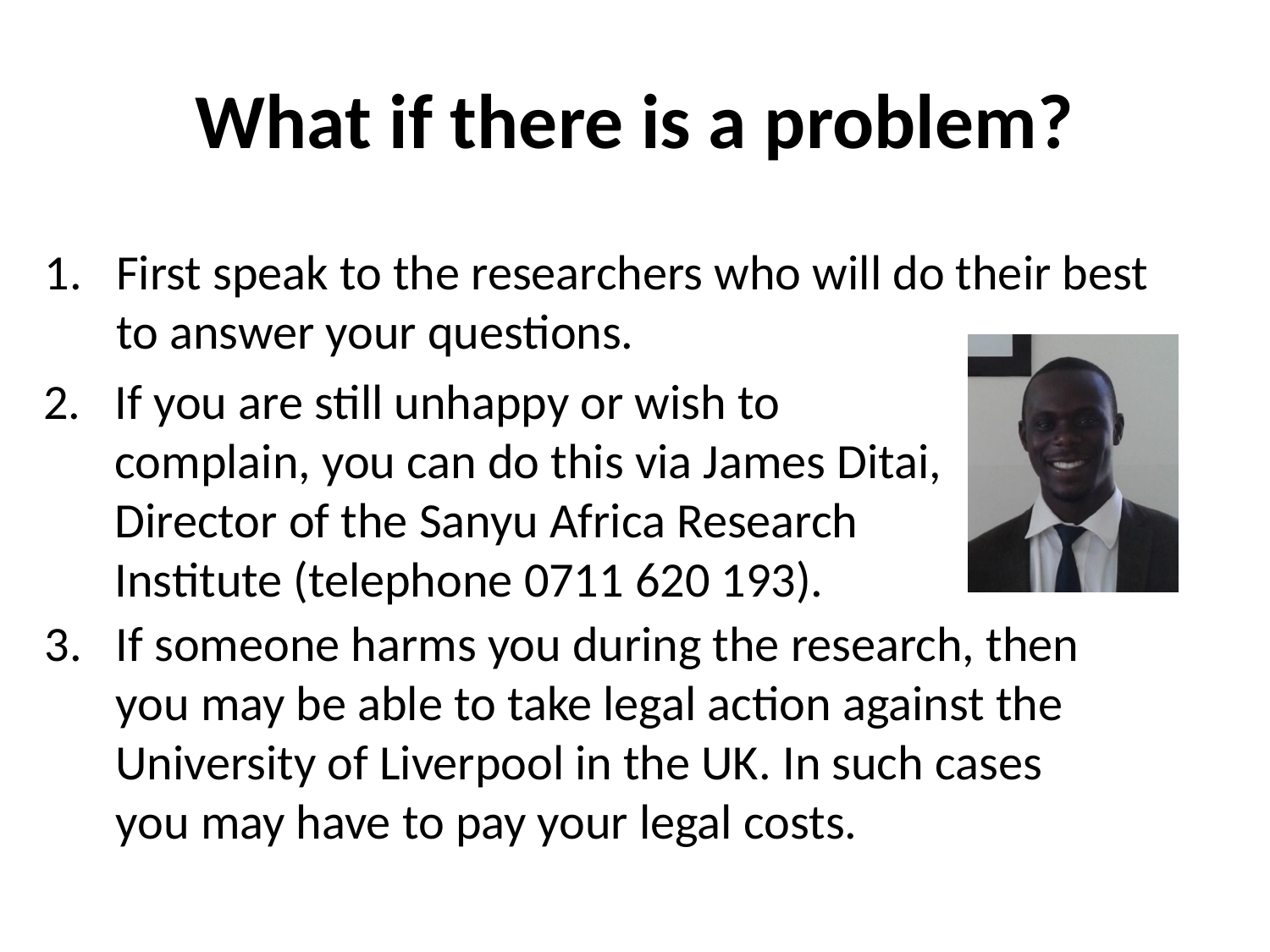

# What if there is a problem?
First speak to the researchers who will do their best to answer your questions.
If you are still unhappy or wish to complain, you can do this via James Ditai, Director of the Sanyu Africa Research Institute (telephone 0711 620 193).
If someone harms you during the research, then you may be able to take legal action against the University of Liverpool in the UK. In such cases you may have to pay your legal costs.

## Slide 17
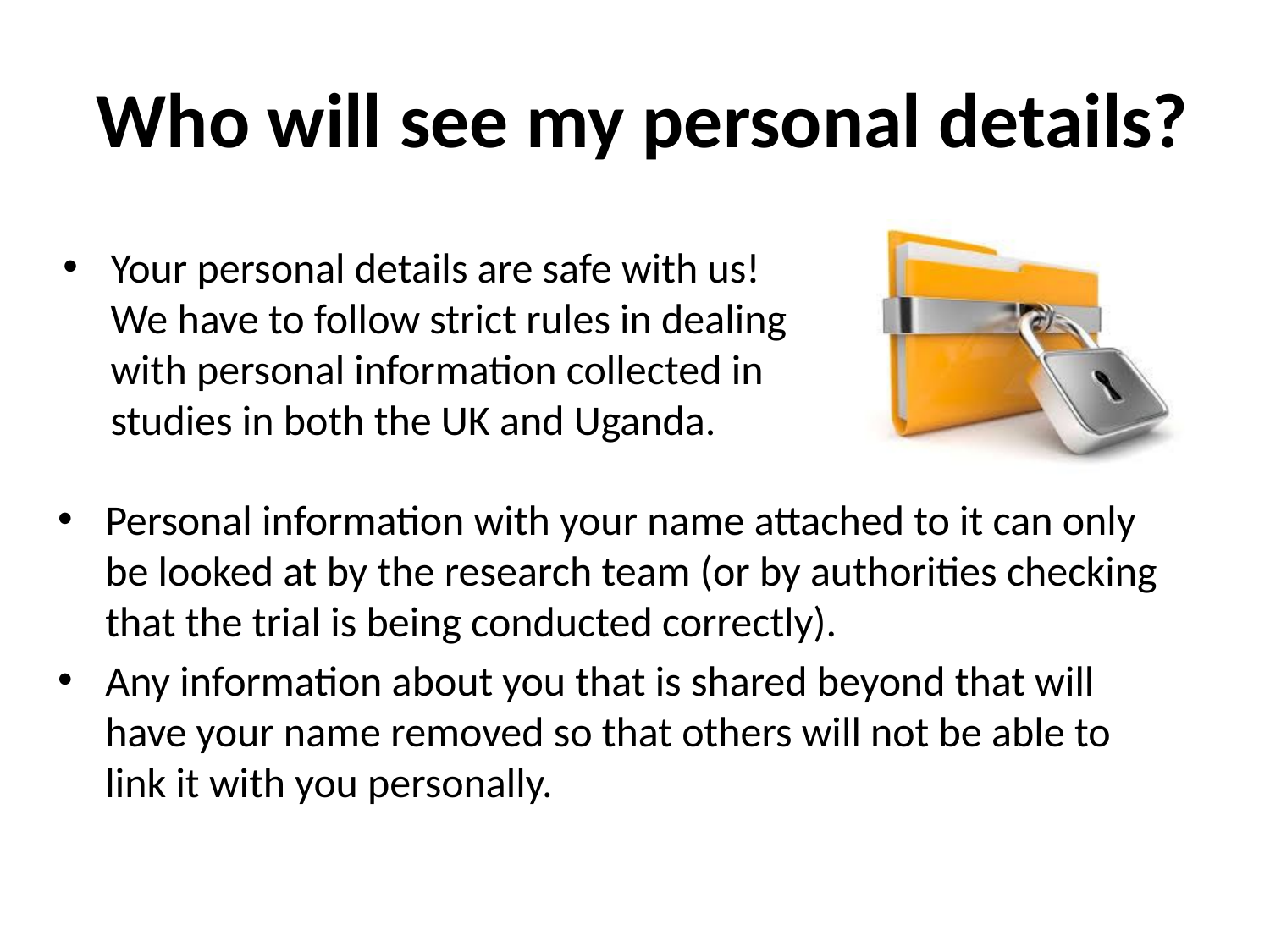

# Who will see my personal details?
Your personal details are safe with us! We have to follow strict rules in dealing with personal information collected in studies in both the UK and Uganda.
Personal information with your name attached to it can only be looked at by the research team (or by authorities checking that the trial is being conducted correctly).
Any information about you that is shared beyond that will have your name removed so that others will not be able to link it with you personally.

## Slide 18
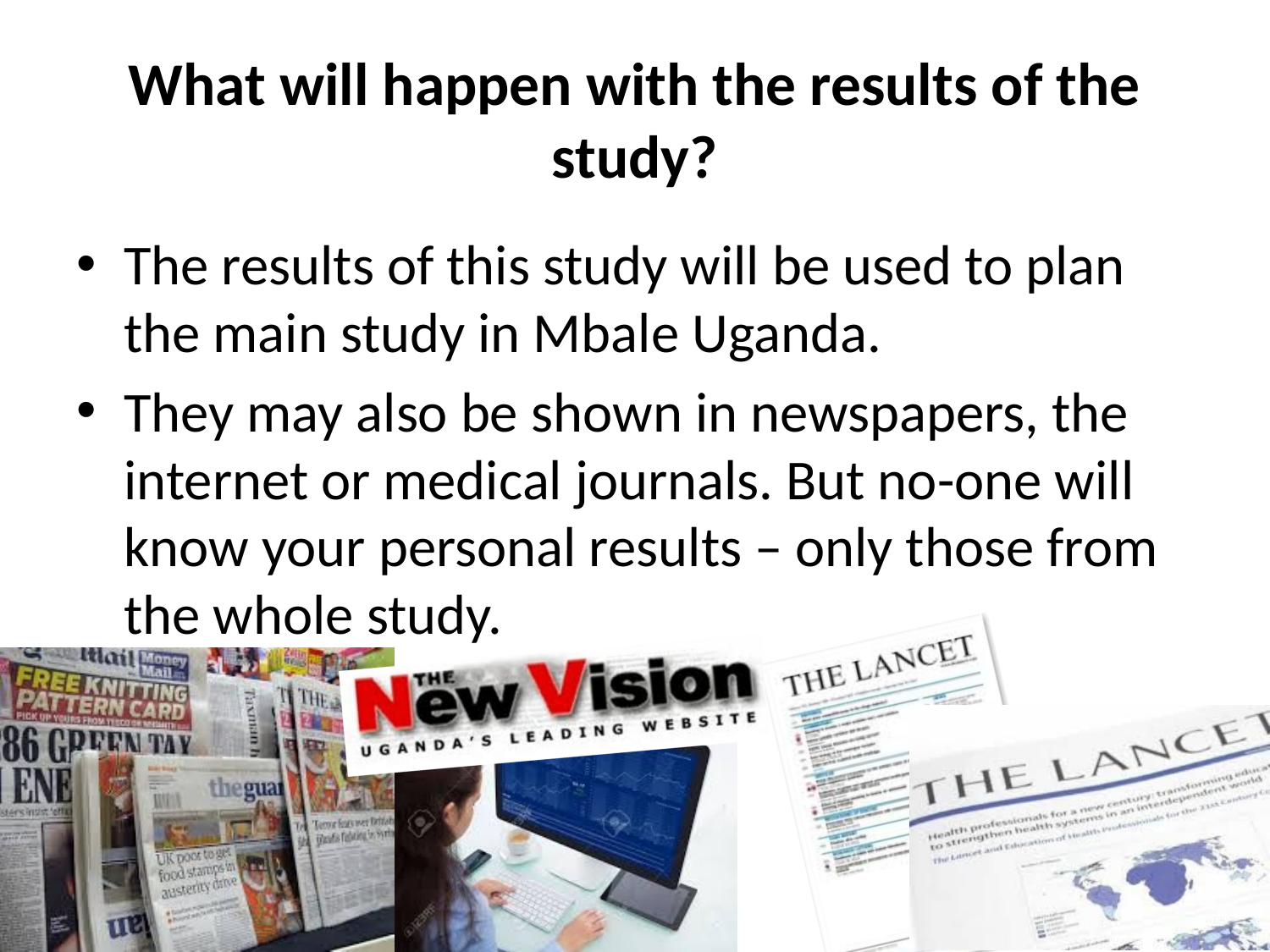

# What will happen with the results of the study?
The results of this study will be used to plan the main study in Mbale Uganda.
They may also be shown in newspapers, the internet or medical journals. But no-one will know your personal results – only those from the whole study.

## Slide 19
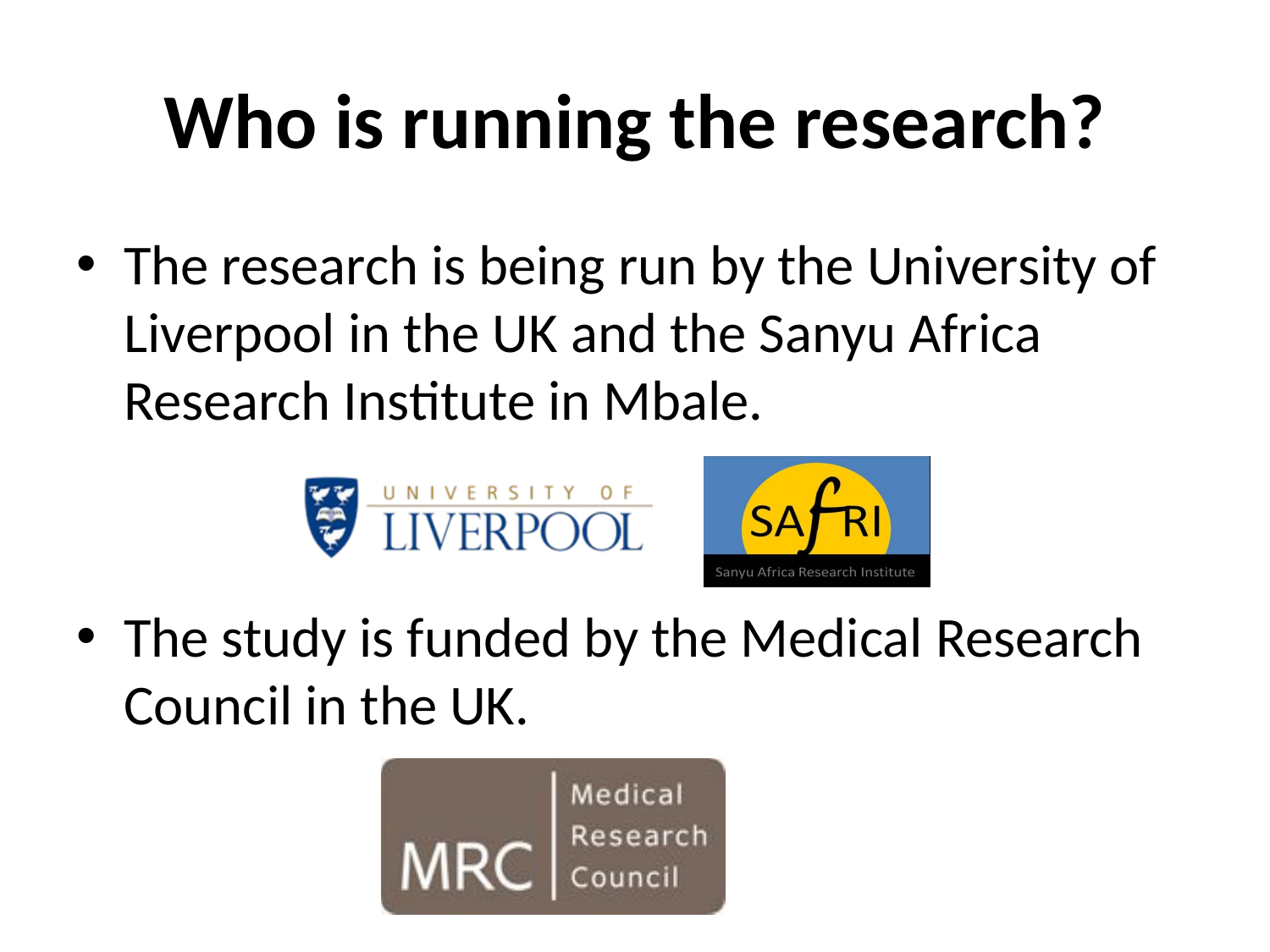

# Who is running the research?
The research is being run by the University of Liverpool in the UK and the Sanyu Africa Research Institute in Mbale.
The study is funded by the Medical Research Council in the UK.

## Slide 20
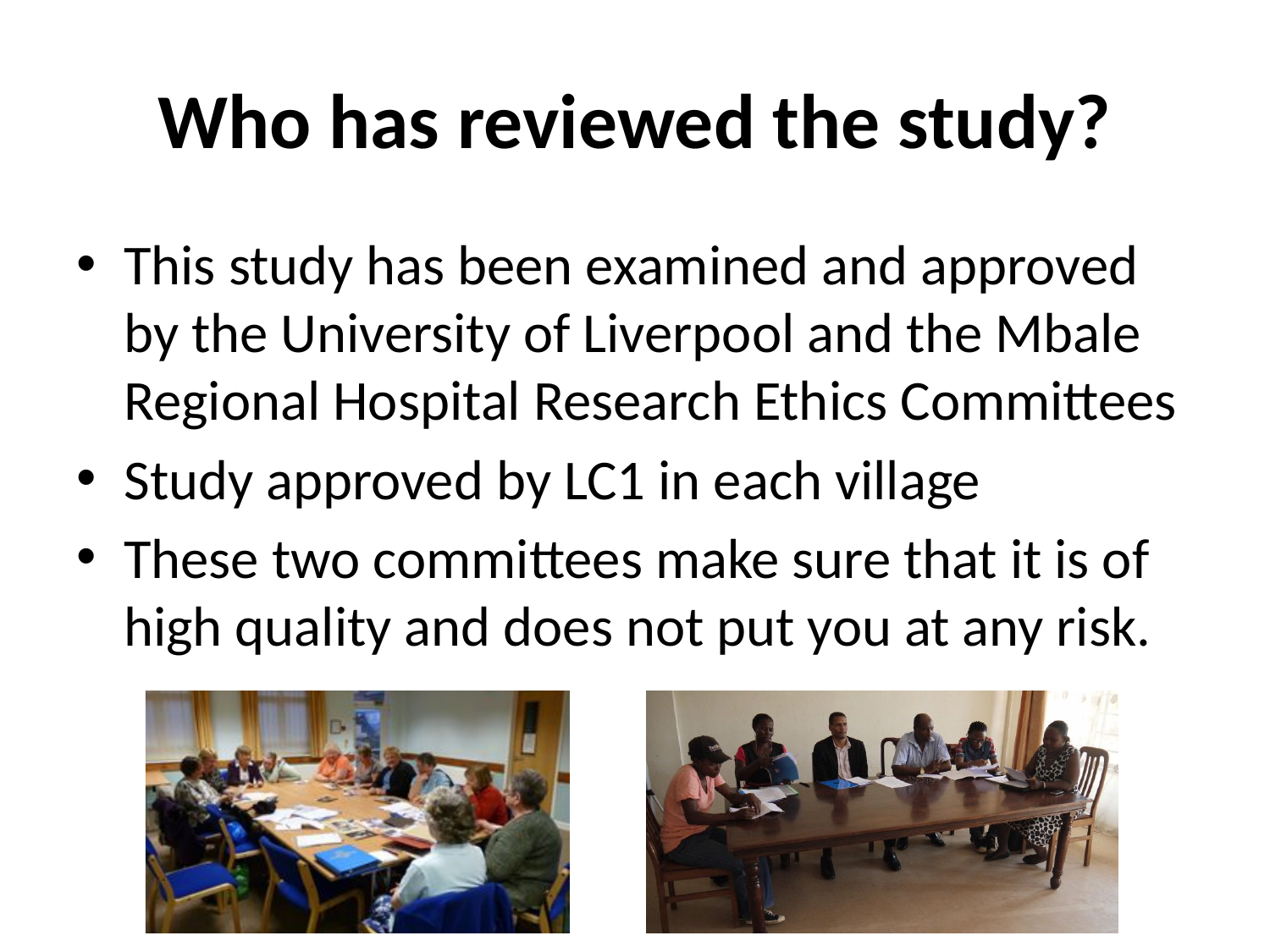

# Who has reviewed the study?
This study has been examined and approved by the University of Liverpool and the Mbale Regional Hospital Research Ethics Committees
Study approved by LC1 in each village
These two committees make sure that it is of high quality and does not put you at any risk.

## Slide 21
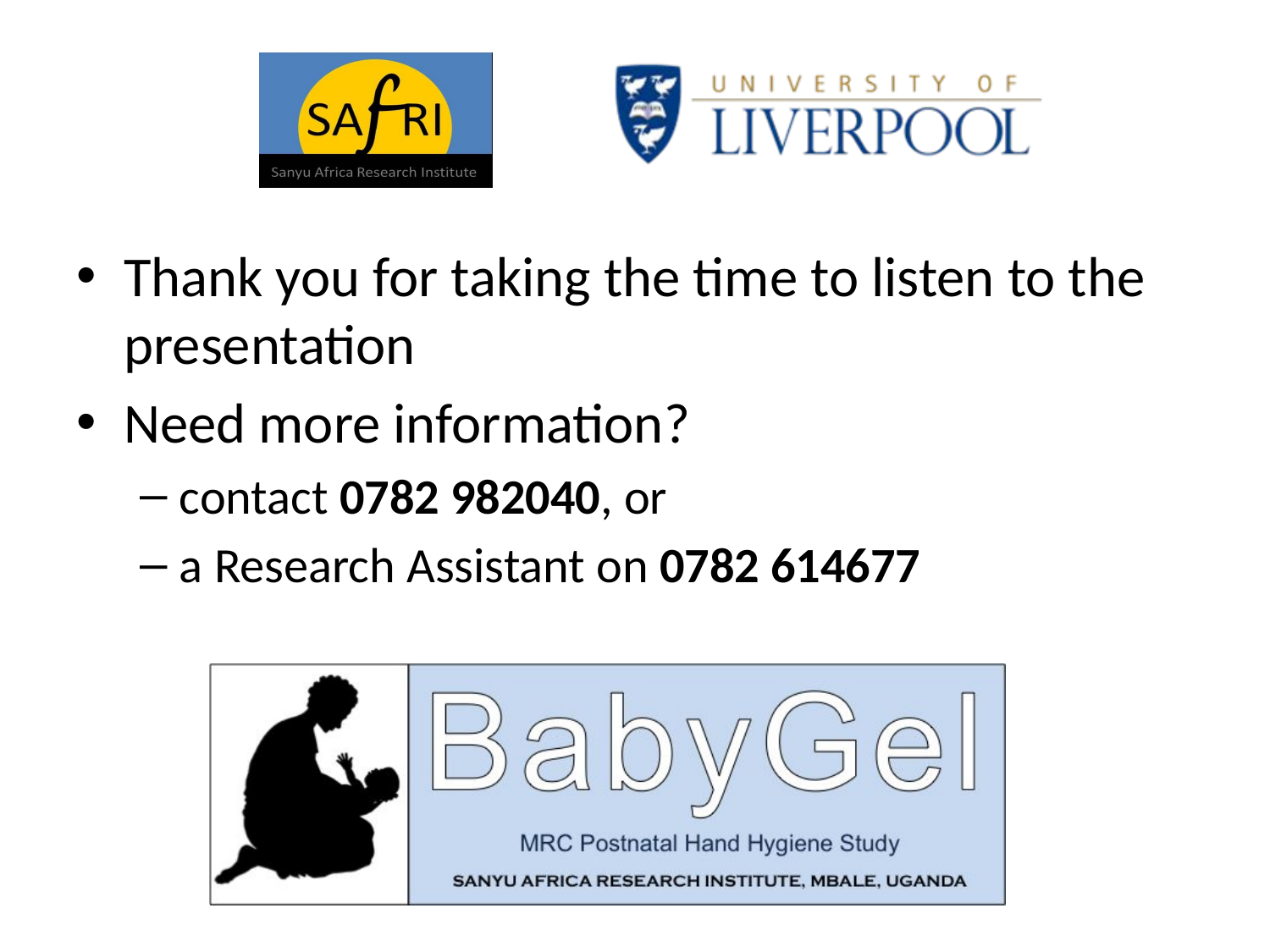

Thank you for taking the time to listen to the presentation
Need more information?
contact 0782 982040, or
a Research Assistant on 0782 614677
