# Supplementary material for: Optimising informed consent for participants in a randomised controlled trial in rural Uganda: a comparative prospective cohort mixed-methods study
Source: Trials. 2018 Dec 22;19:699. doi: 10.1186/s13063-018-3030-8 (PMC6304001; doi:10.1186/s13063-018-3030-8)
Supplement: Supplementary file 2 — The approved slides (English version and Lumasaba version). (ZIP 12399 kb) [file 13063_2018_3030_MOESM2_ESM.zip › BabyGel Lumasaba PIS Slide v8R2.pptx]

## Slide 1
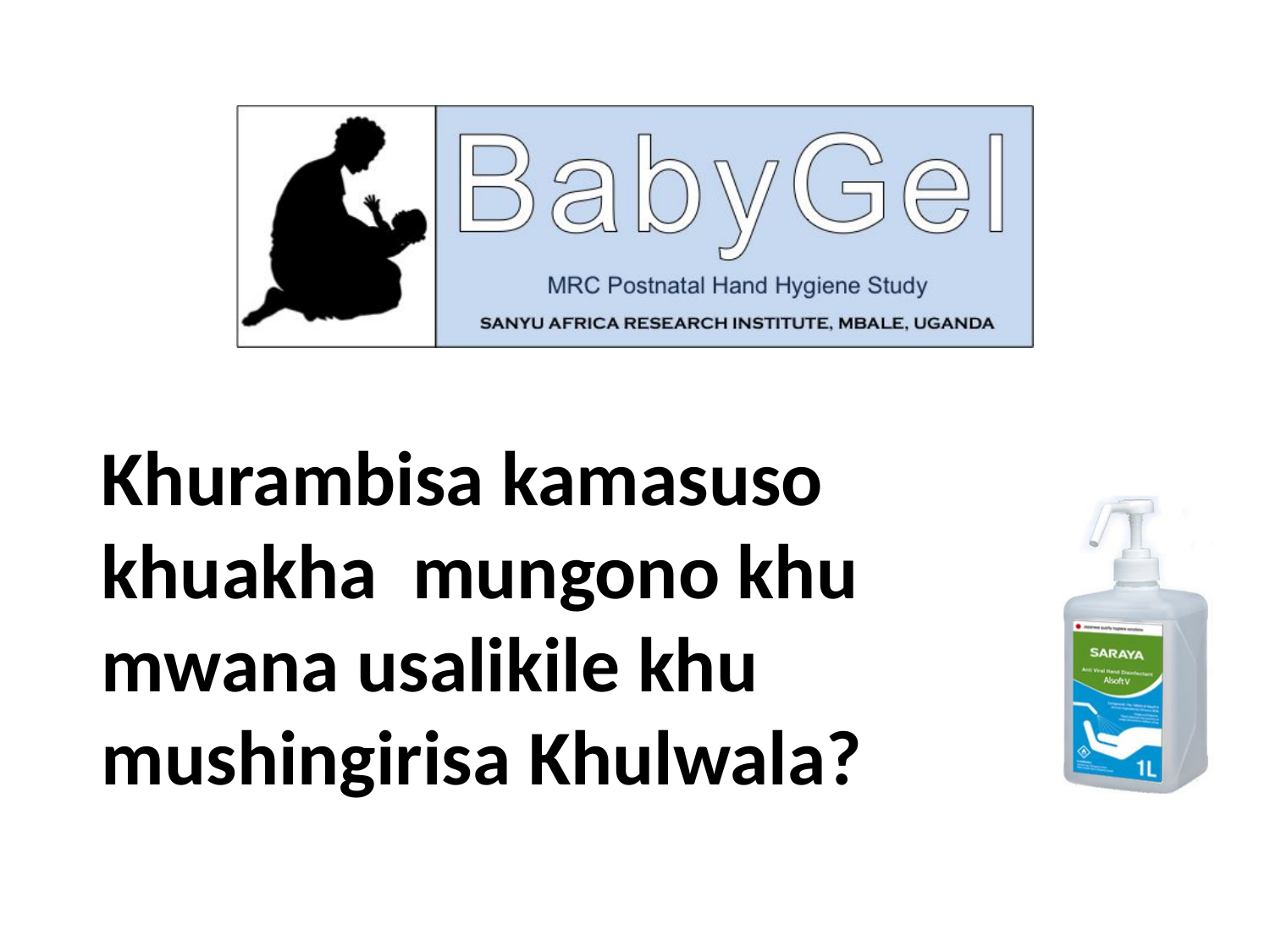

# Khurambisa kamasuso khuakha mungono khu mwana usalikile khu mushingirisa Khulwala?

## Slide 2
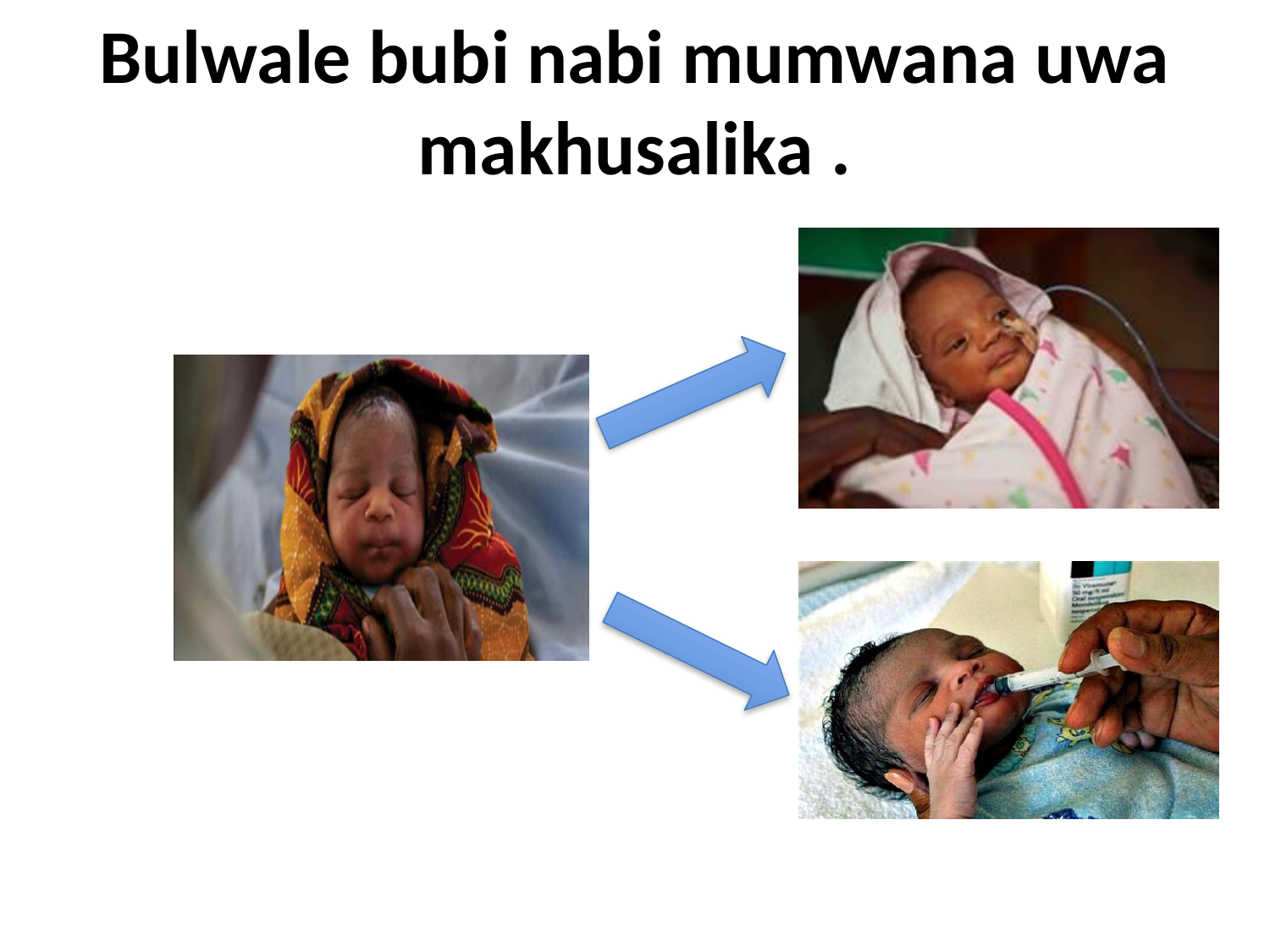

# Bulwale bubi nabi mumwana uwa makhusalika .

## Slide 3
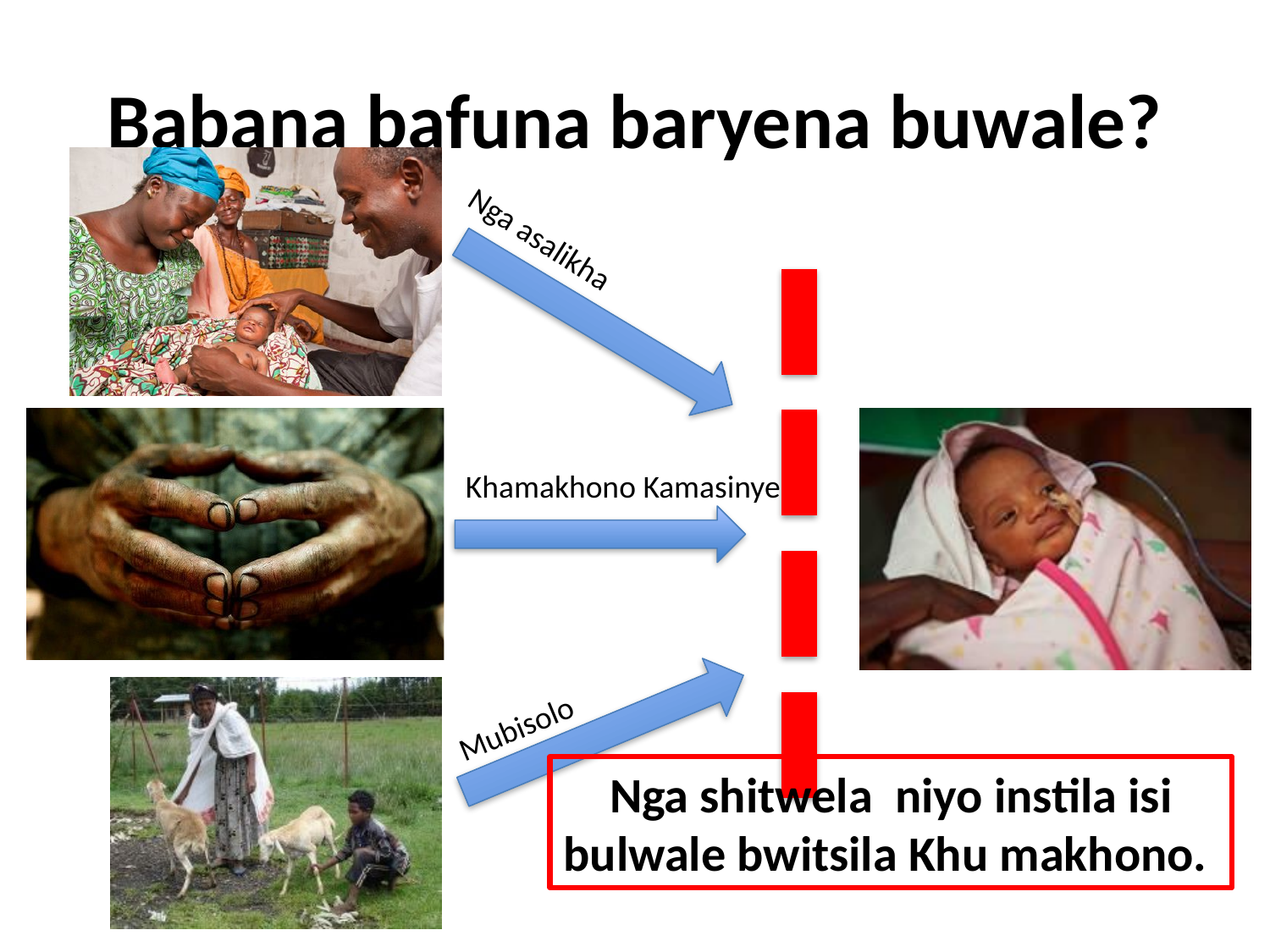

# Babana bafuna baryena buwale?
Nga asalikha
Nga shitwela niyo instila isi bulwale bwitsila Khu makhono.
Khamakhono Kamasinye
Mubisolo

## Slide 4
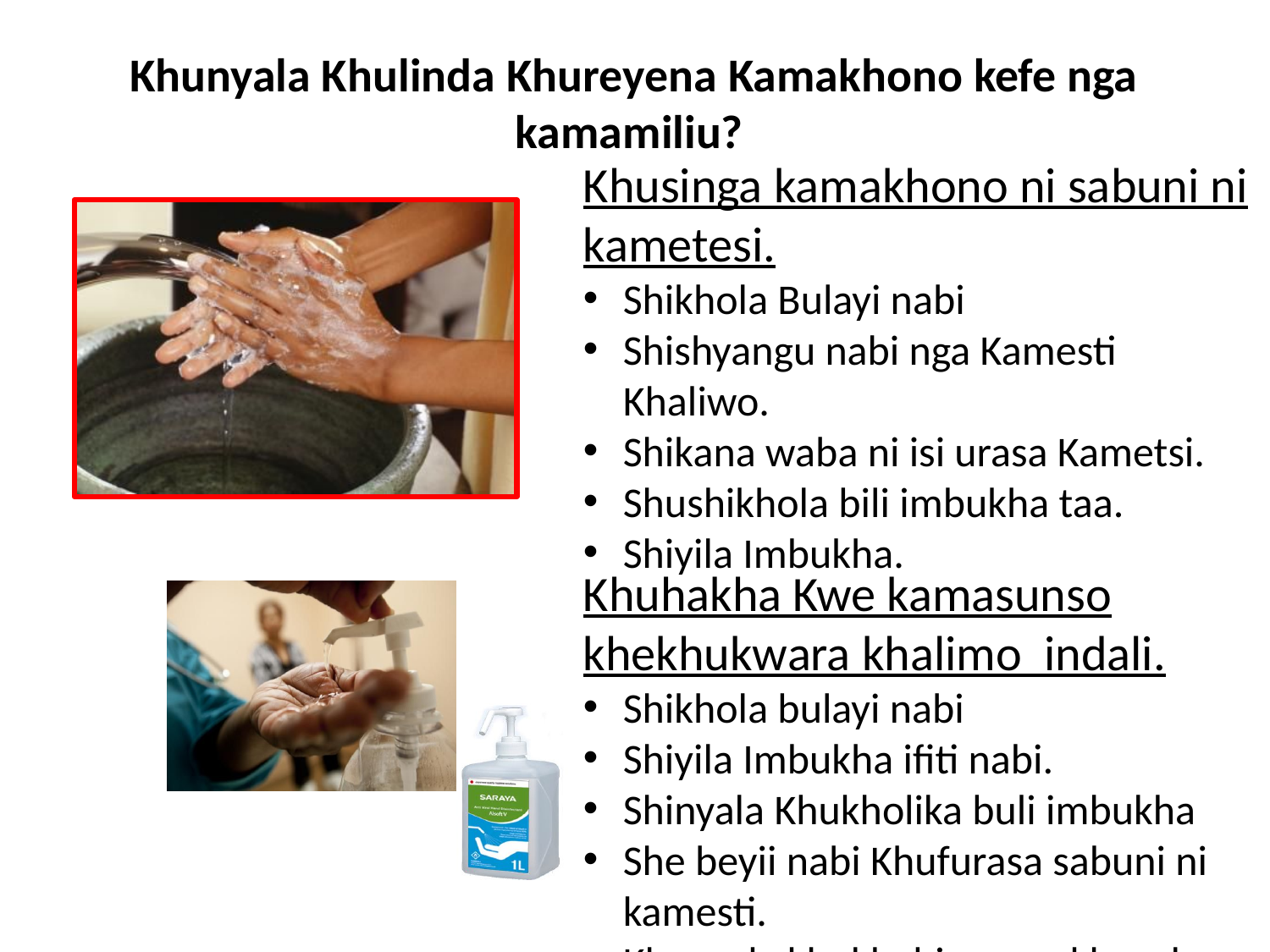

Khunyala Khulinda Khureyena Kamakhono kefe nga kamamiliu?
Khusinga kamakhono ni sabuni ni kametesi.
Shikhola Bulayi nabi
Shishyangu nabi nga Kamesti Khaliwo.
Shikana waba ni isi urasa Kametsi.
Shushikhola bili imbukha taa.
Shiyila Imbukha.
Khuhakha Kwe kamasunso khekhukwara khalimo indali.
Shikhola bulayi nabi
Shiyila Imbukha ifiti nabi.
Shinyala Khukholika buli imbukha
She beyii nabi Khufurasa sabuni ni kamesti.
Khanyala khukhabiwa mu khusala.

## Slide 5
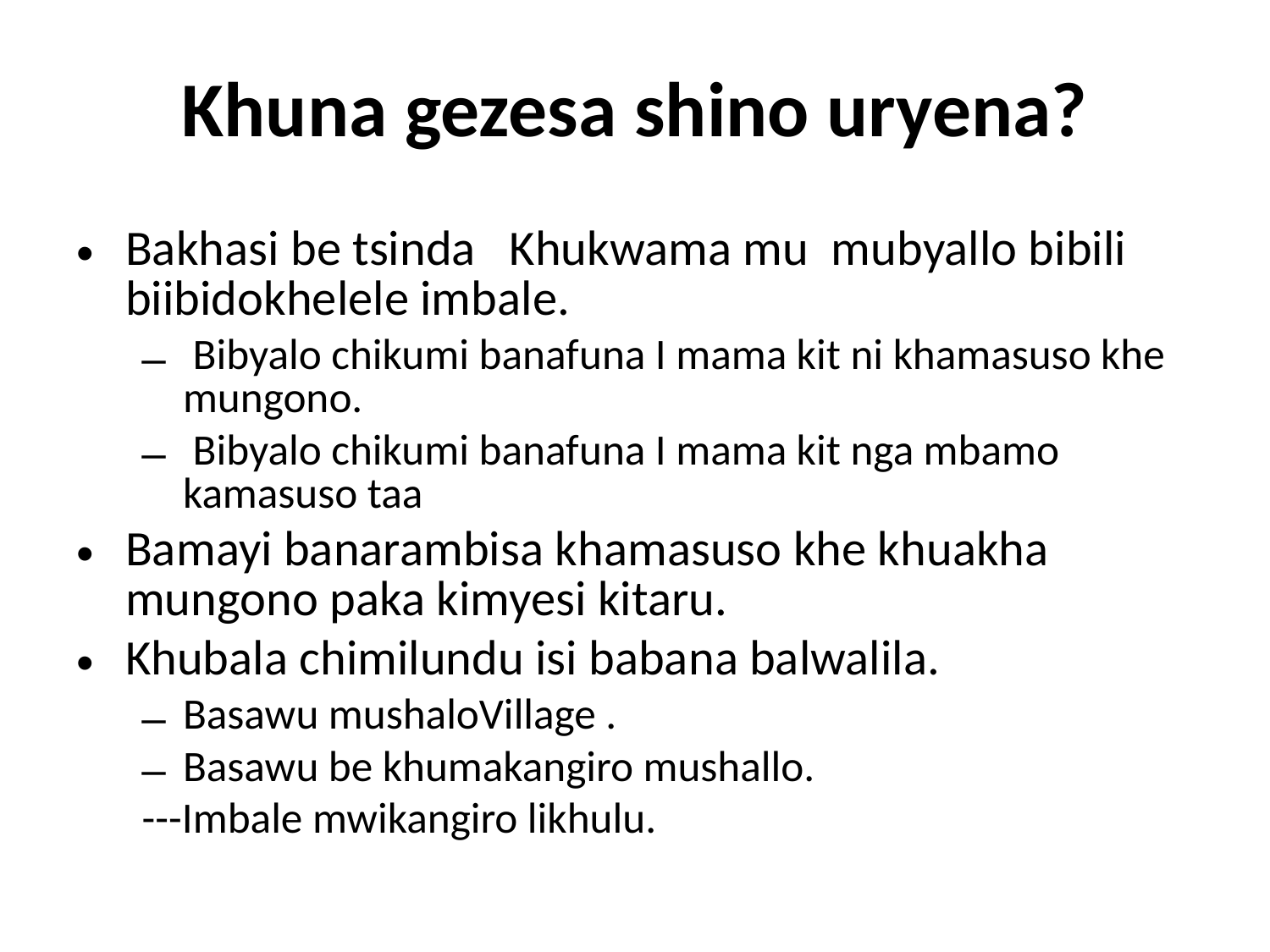

# Khuna gezesa shino uryena?
Bakhasi be tsinda Khukwama mu mubyallo bibili biibidokhelele imbale.
 Bibyalo chikumi banafuna I mama kit ni khamasuso khe mungono.
 Bibyalo chikumi banafuna I mama kit nga mbamo kamasuso taa
Bamayi banarambisa khamasuso khe khuakha mungono paka kimyesi kitaru.
Khubala chimilundu isi babana balwalila.
Basawu mushaloVillage .
Basawu be khumakangiro mushallo.
---Imbale mwikangiro likhulu.

## Slide 6
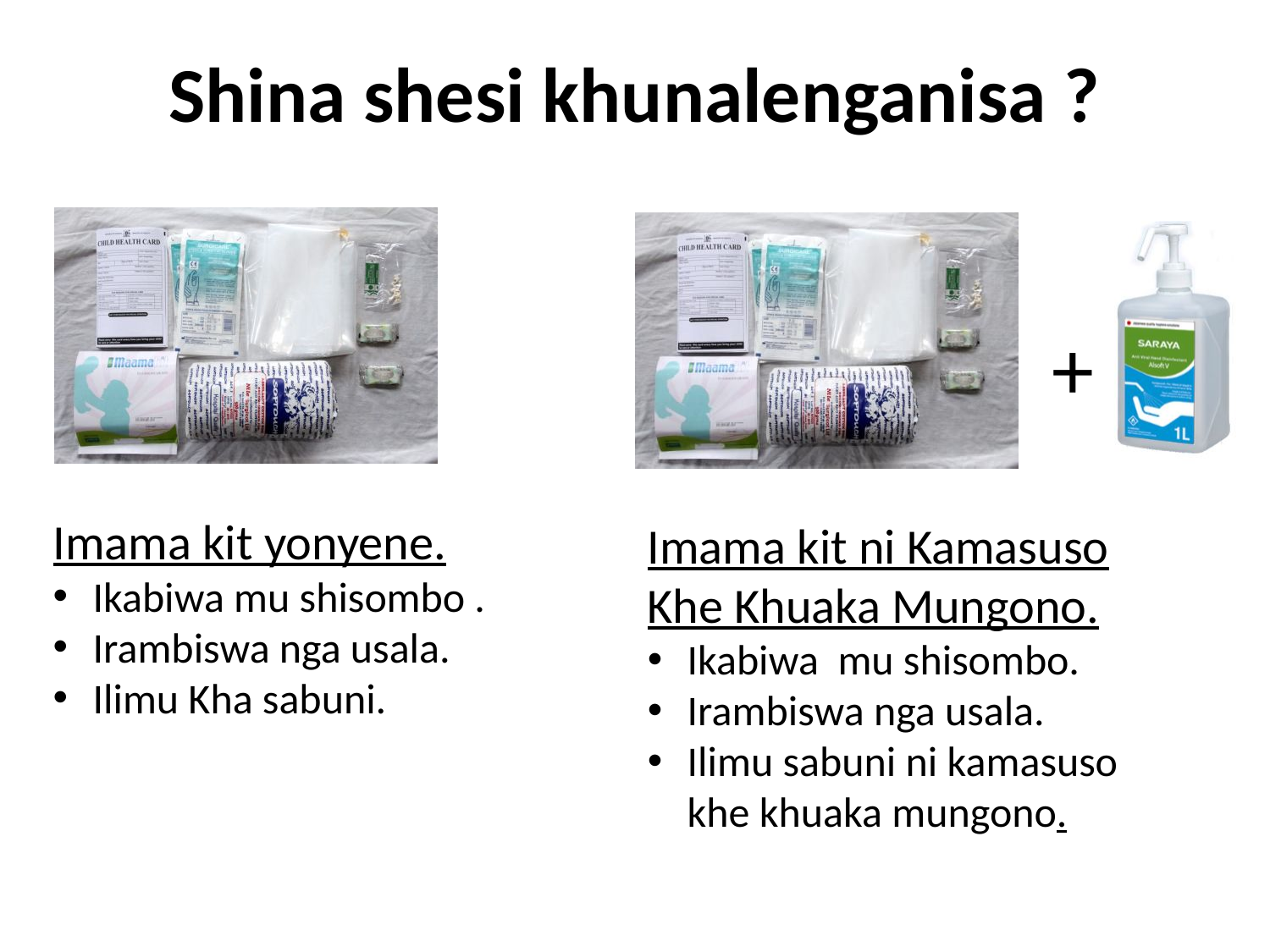

Shina shesi khunalenganisa ?
+
Imama kit yonyene.
Ikabiwa mu shisombo .
Irambiswa nga usala.
Ilimu Kha sabuni.
Imama kit ni Kamasuso Khe Khuaka Mungono.
Ikabiwa mu shisombo.
Irambiswa nga usala.
Ilimu sabuni ni kamasuso khe khuaka mungono.
Nga babandu be tsingo barambisa kamasuso khe mungono kymesi kitaru kiyoa mubulamu bwo umwana, shina khukhendesa buwale mumwana?

## Slide 7
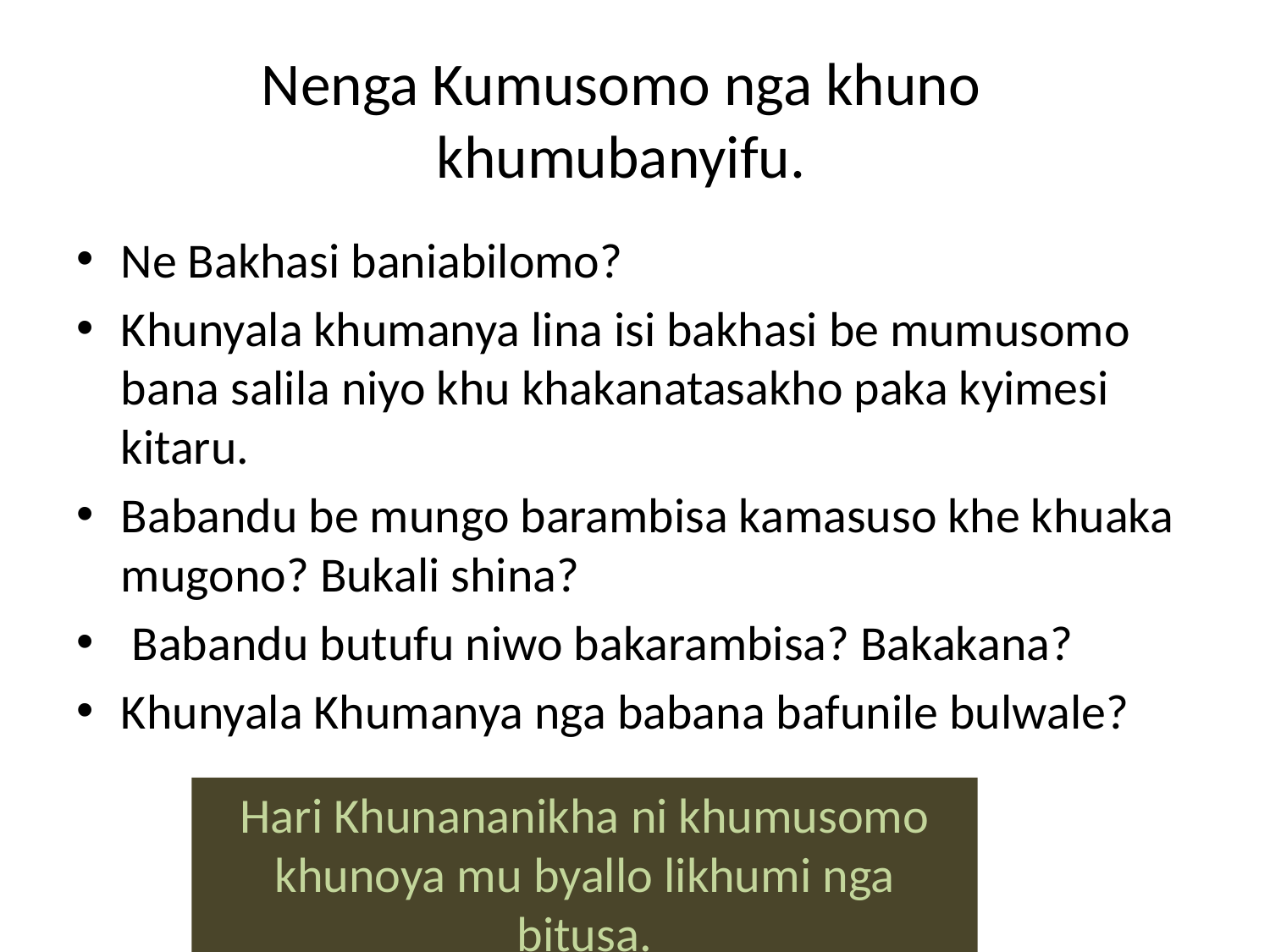

# Nenga Kumusomo nga khuno khumubanyifu.
Ne Bakhasi baniabilomo?
Khunyala khumanya lina isi bakhasi be mumusomo bana salila niyo khu khakanatasakho paka kyimesi kitaru.
Babandu be mungo barambisa kamasuso khe khuaka mugono? Bukali shina?
 Babandu butufu niwo bakarambisa? Bakakana?
Khunyala Khumanya nga babana bafunile bulwale?
Hari Khunananikha ni khumusomo khunoya mu byallo likhumi nga bitusa.

## Slide 8
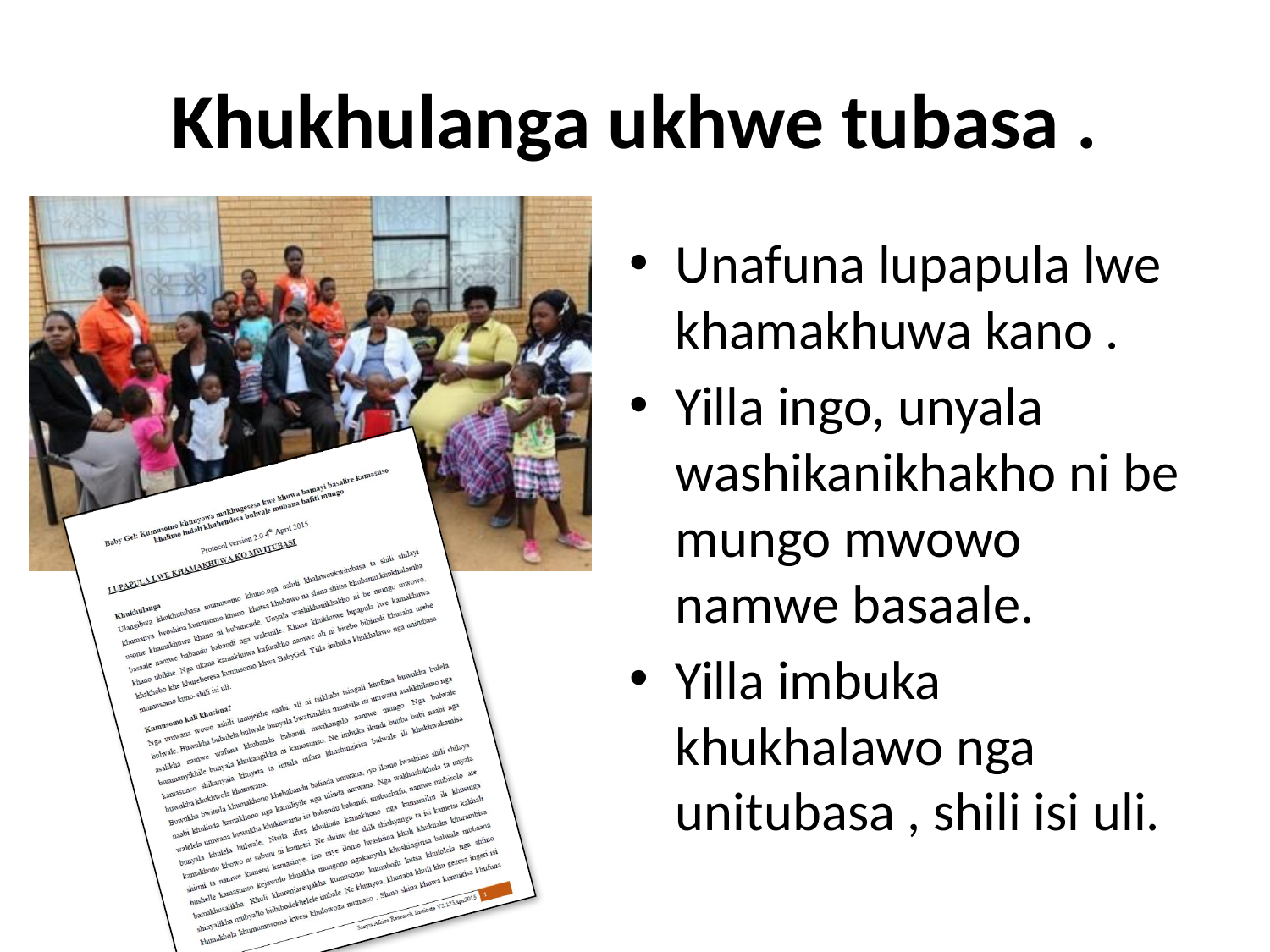

# Khukhulanga ukhwe tubasa .
Unafuna lupapula lwe khamakhuwa kano .
Yilla ingo, unyala washikanikhakho ni be mungo mwowo namwe basaale.
Yilla imbuka khukhalawo nga unitubasa , shili isi uli.

## Slide 9
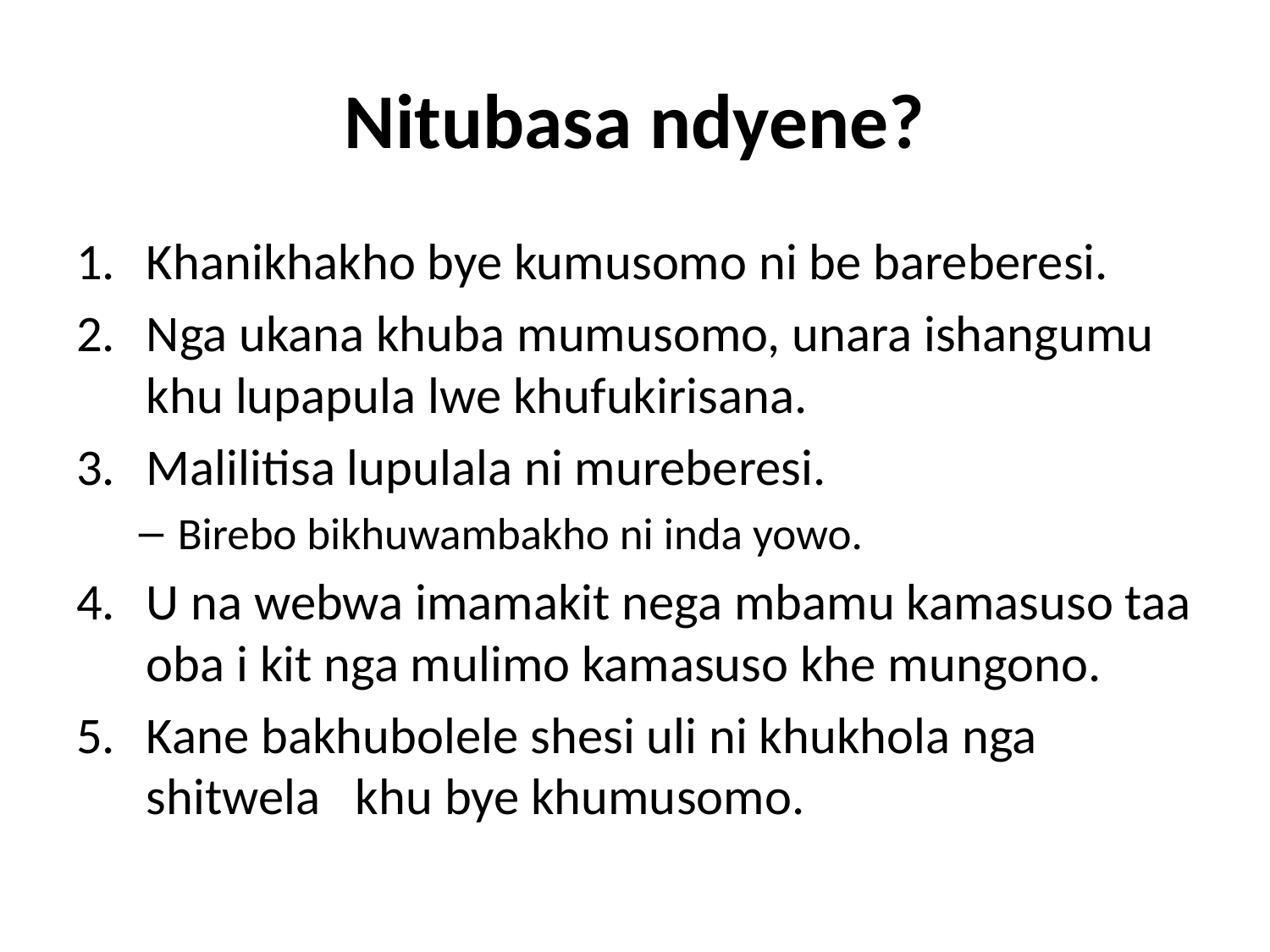

# Nitubasa ndyene?
Khanikhakho bye kumusomo ni be bareberesi.
Nga ukana khuba mumusomo, unara ishangumu khu lupapula lwe khufukirisana.
Malilitisa lupulala ni mureberesi.
Birebo bikhuwambakho ni inda yowo.
U na webwa imamakit nega mbamu kamasuso taa oba i kit nga mulimo kamasuso khe mungono.
Kane bakhubolele shesi uli ni khukhola nga shitwela khu bye khumusomo.

## Slide 10
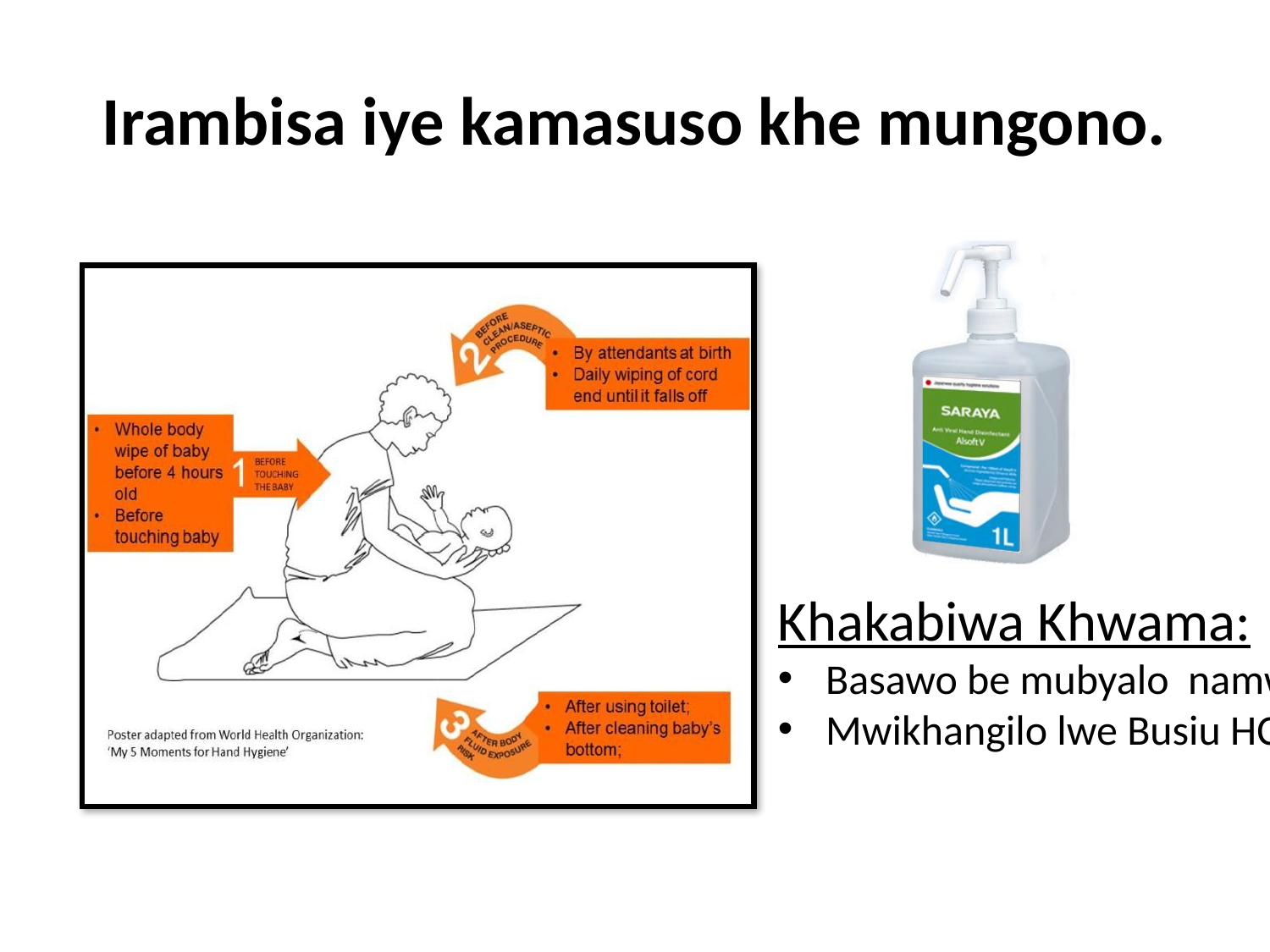

# Irambisa iye kamasuso khe mungono.
Khakabiwa Khwama:
Basawo be mubyalo namwe
Mwikhangilo lwe Busiu HC.

## Slide 11
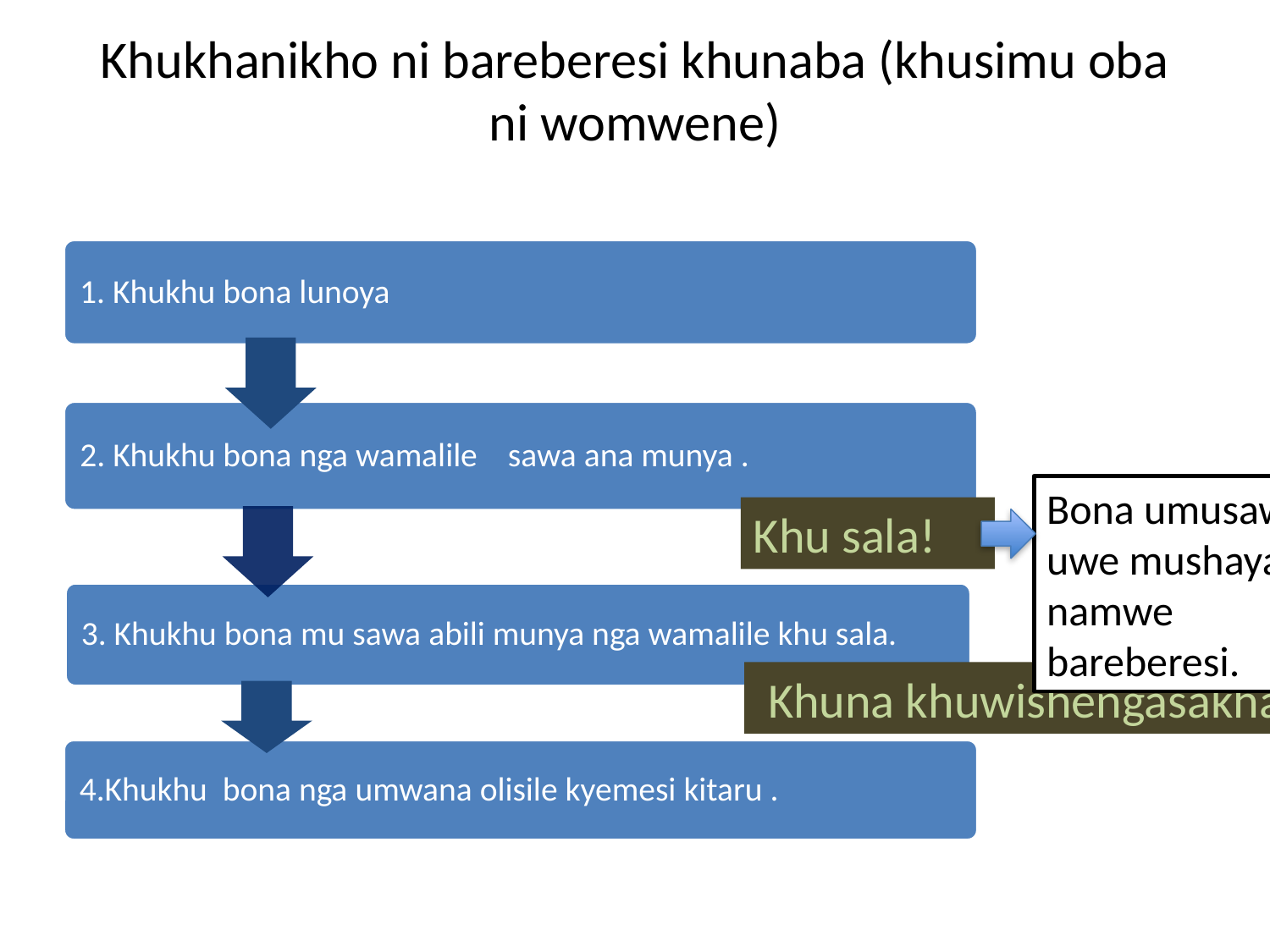

# Khukhanikho ni bareberesi khunaba (khusimu oba ni womwene)
Bona umusawo uwe mushayalo namwe bareberesi.
Khu sala!
 Khuna khuwishengasakha

## Slide 12
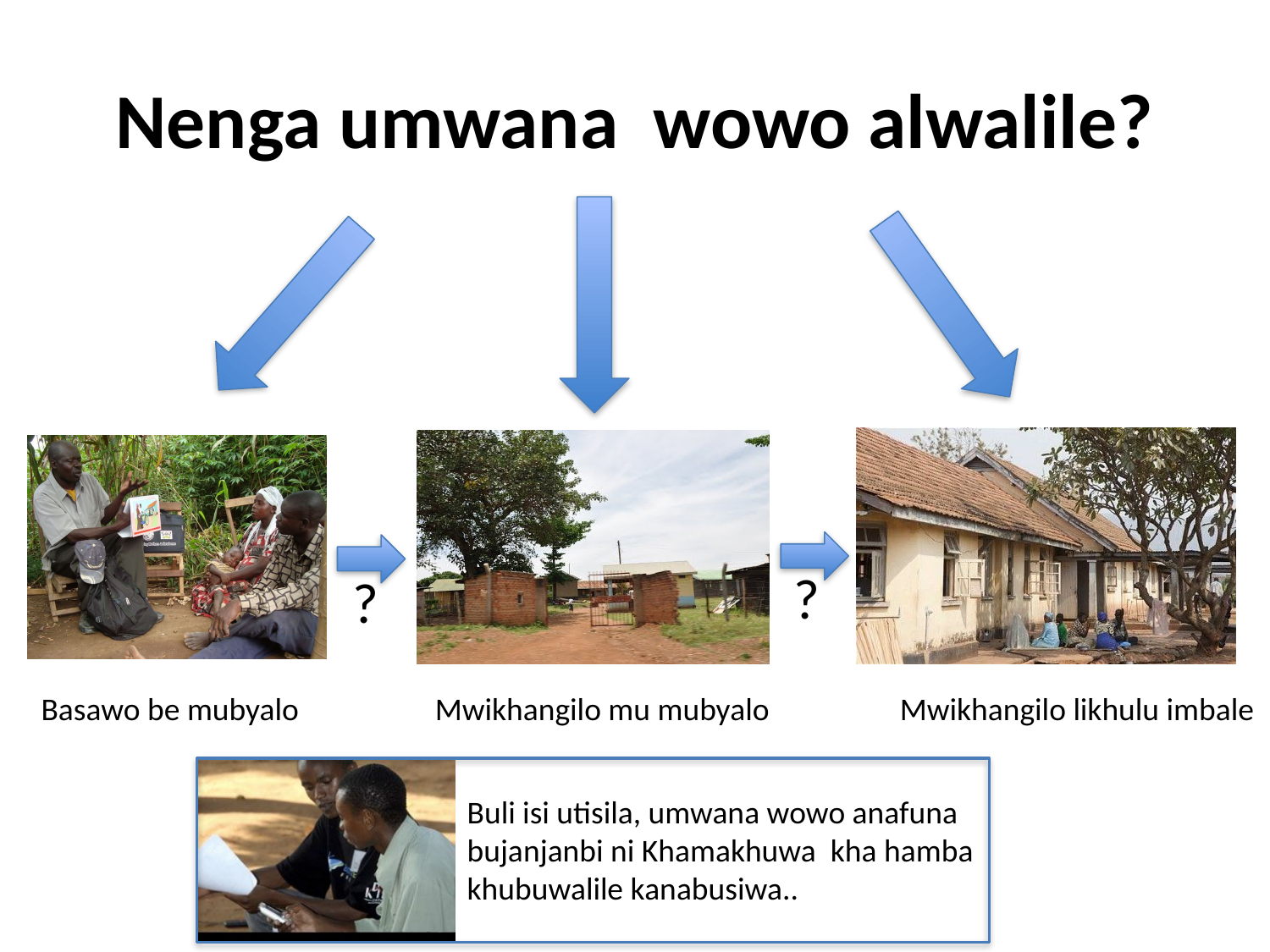

# Nenga umwana wowo alwalile?
Mwikhangilo likhulu imbale
Basawo be mubyalo Mwikhangilo mu mubyalo
?
?
Buli isi utisila, umwana wowo anafuna bujanjanbi ni Khamakhuwa kha hamba khubuwalile kanabusiwa..

## Slide 13
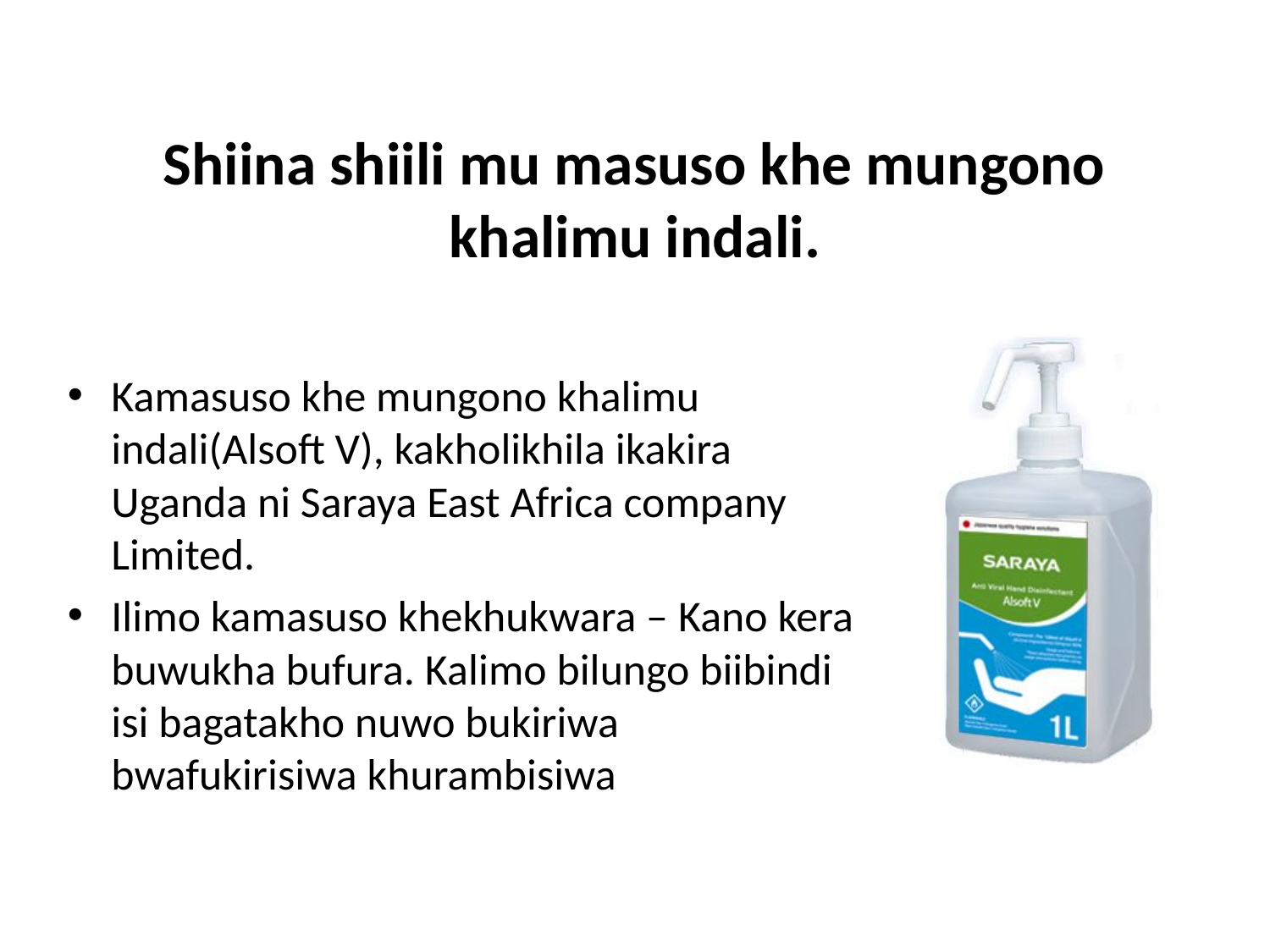

# Shiina shiili mu masuso khe mungono khalimu indali.
Kamasuso khe mungono khalimu indali(Alsoft V), kakholikhila ikakira Uganda ni Saraya East Africa company Limited.
Ilimo kamasuso khekhukwara – Kano kera buwukha bufura. Kalimo bilungo biibindi isi bagatakho nuwo bukiriwa bwafukirisiwa khurambisiwa

## Slide 14
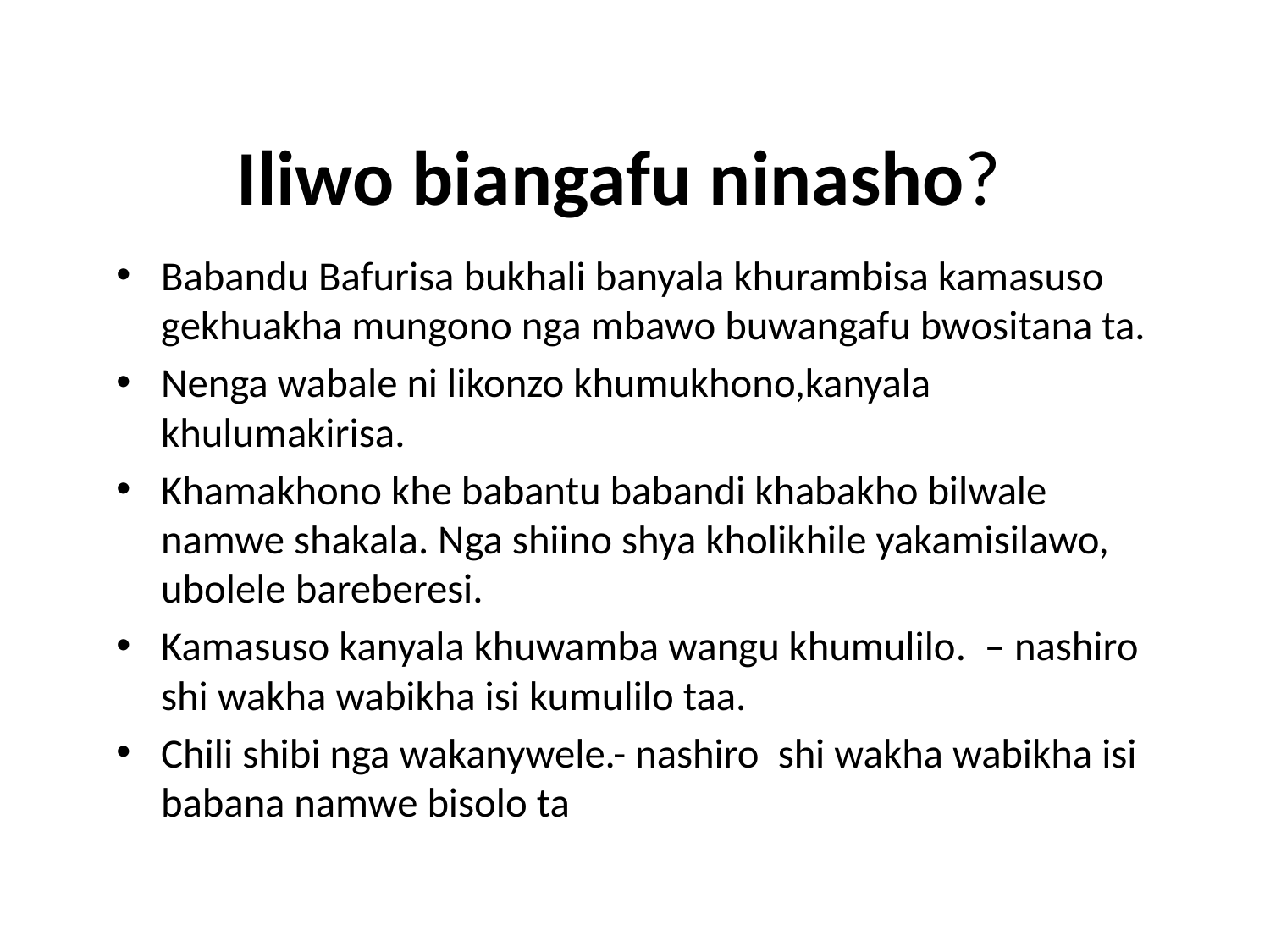

# Iliwo biangafu ninasho?
Babandu Bafurisa bukhali banyala khurambisa kamasuso gekhuakha mungono nga mbawo buwangafu bwositana ta.
Nenga wabale ni likonzo khumukhono,kanyala khulumakirisa.
Khamakhono khe babantu babandi khabakho bilwale namwe shakala. Nga shiino shya kholikhile yakamisilawo, ubolele bareberesi.
Kamasuso kanyala khuwamba wangu khumulilo. – nashiro shi wakha wabikha isi kumulilo taa.
Chili shibi nga wakanywele.- nashiro shi wakha wabikha isi babana namwe bisolo ta

## Slide 15
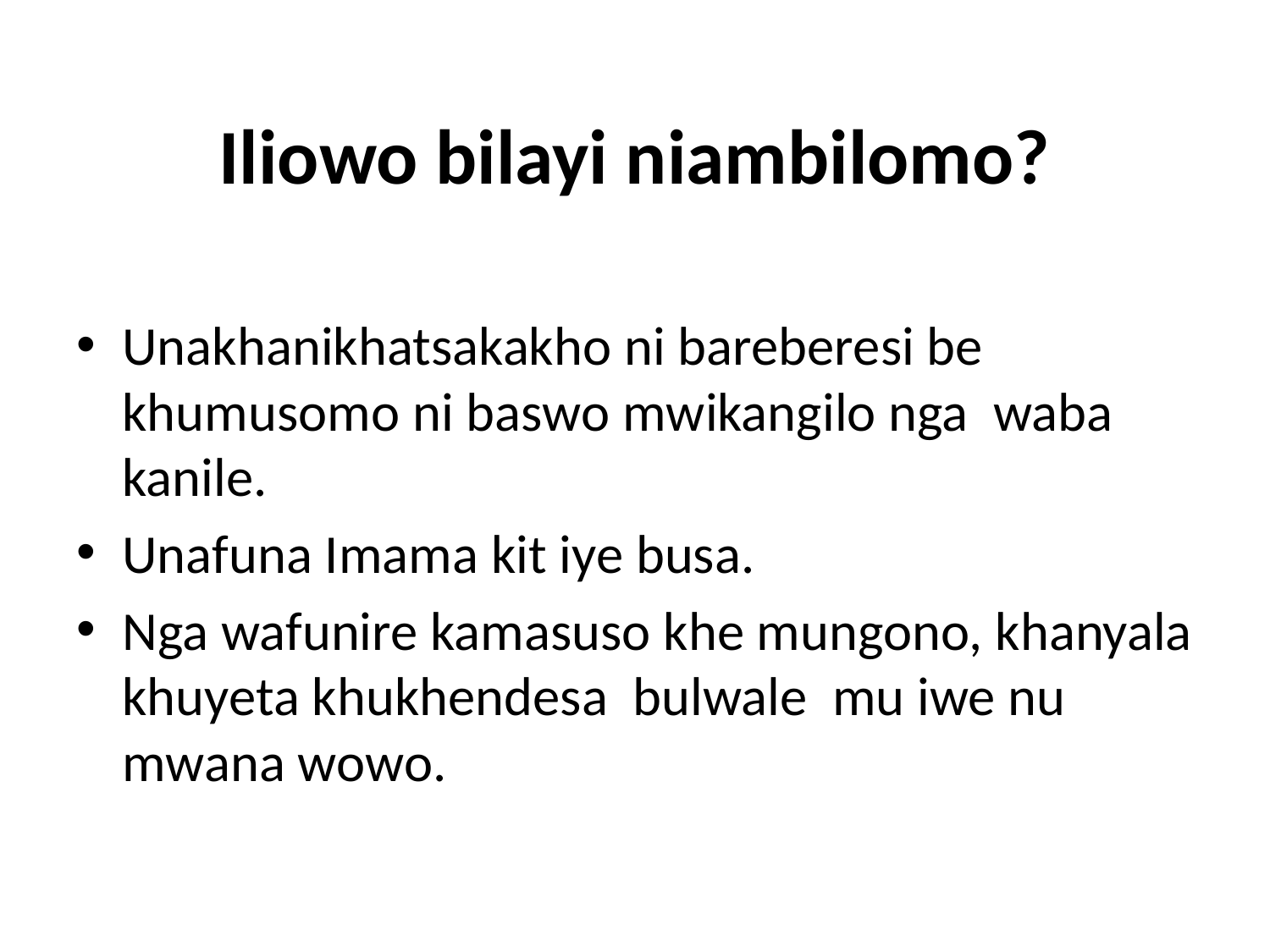

# Iliowo bilayi niambilomo?
Unakhanikhatsakakho ni bareberesi be khumusomo ni baswo mwikangilo nga waba kanile.
Unafuna Imama kit iye busa.
Nga wafunire kamasuso khe mungono, khanyala khuyeta khukhendesa bulwale mu iwe nu mwana wowo.

## Slide 16
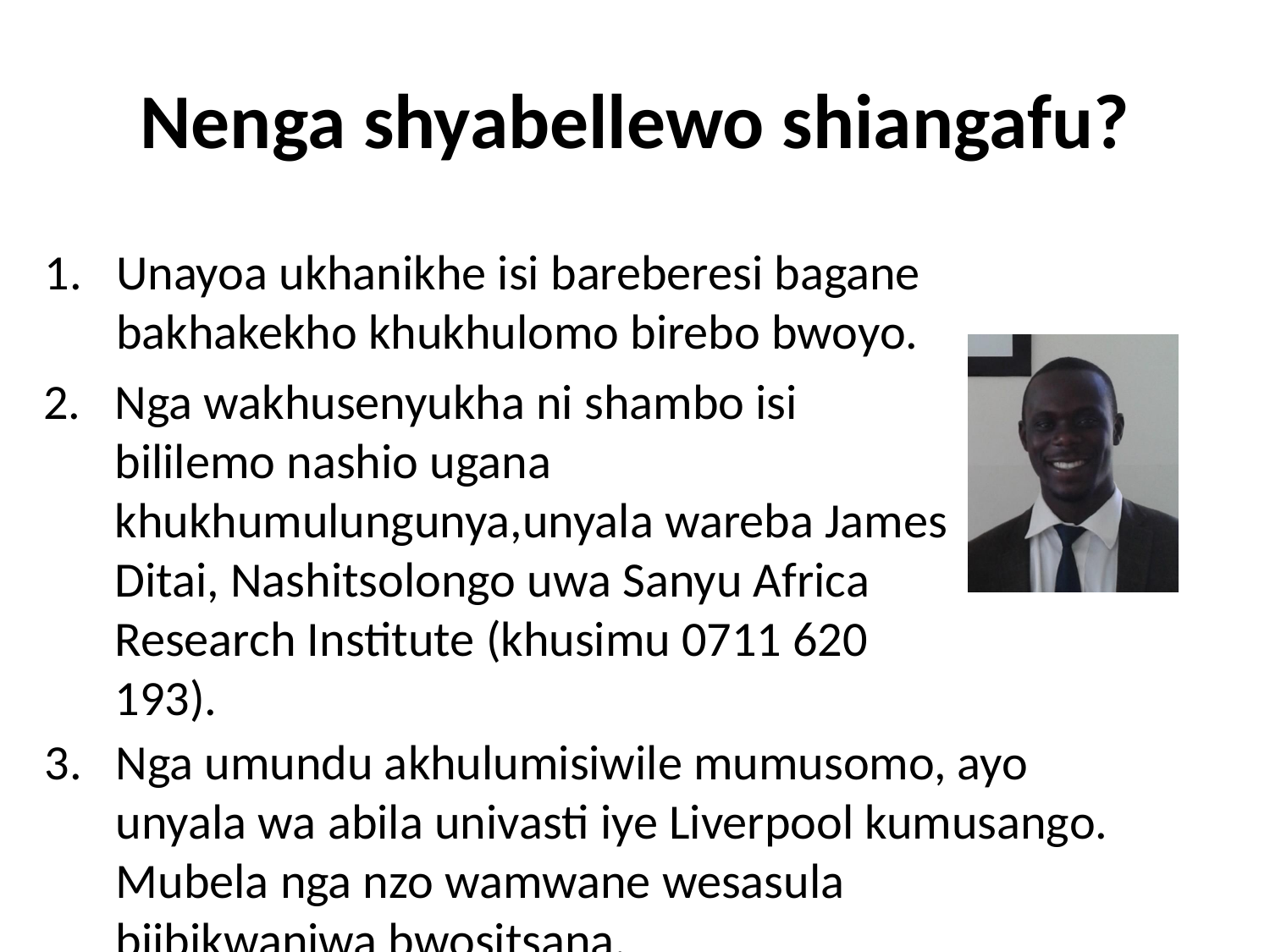

# Nenga shyabellewo shiangafu?
Unayoa ukhanikhe isi bareberesi bagane bakhakekho khukhulomo birebo bwoyo.
Nga wakhusenyukha ni shambo isi bililemo nashio ugana khukhumulungunya,unyala wareba James Ditai, Nashitsolongo uwa Sanyu Africa Research Institute (khusimu 0711 620 193).
Nga umundu akhulumisiwile mumusomo, ayo unyala wa abila univasti iye Liverpool kumusango. Mubela nga nzo wamwane wesasula biibikwaniwa bwositsana.

## Slide 17
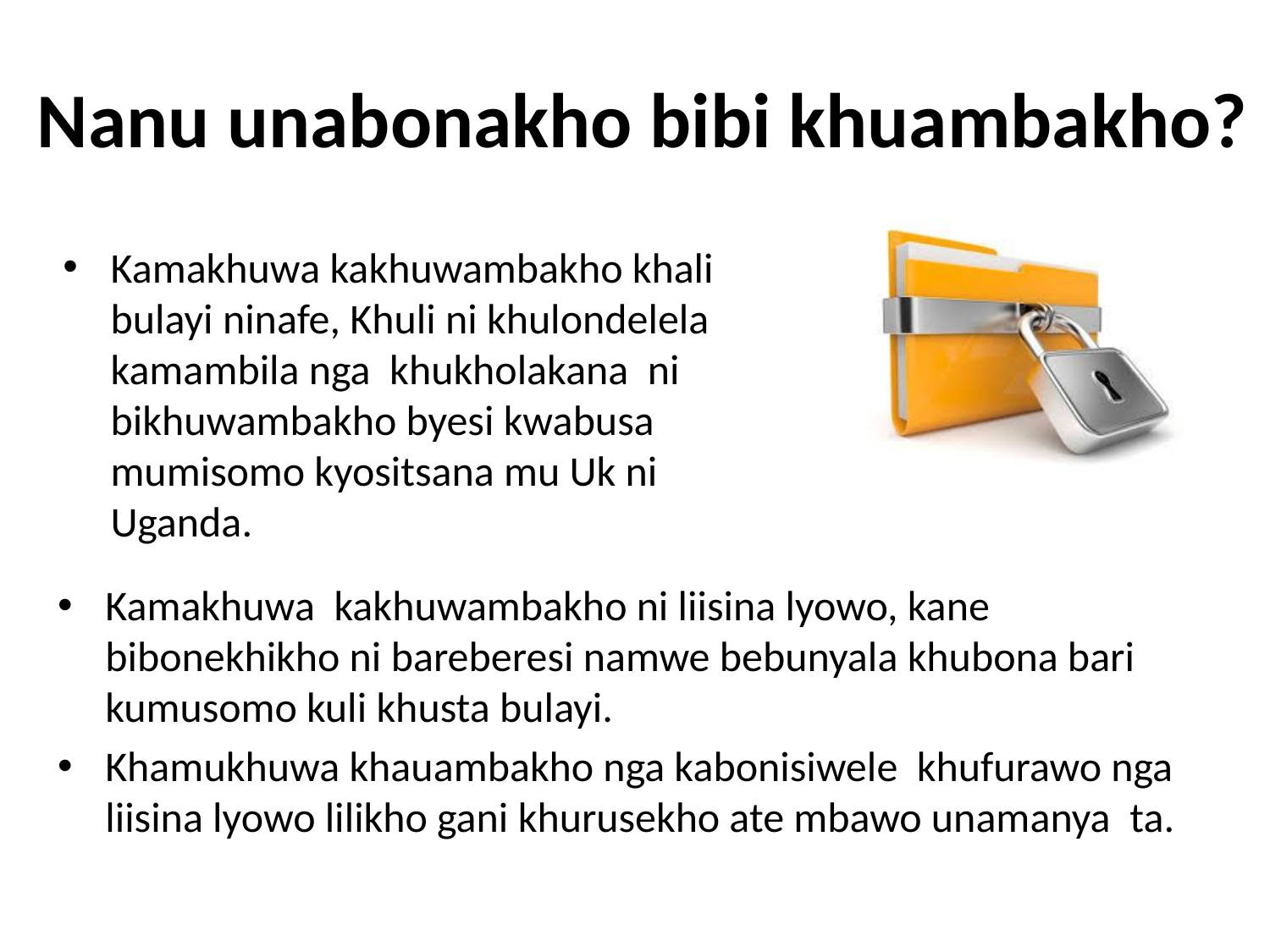

# Nanu unabonakho bibi khuambakho?
Kamakhuwa kakhuwambakho khali bulayi ninafe, Khuli ni khulondelela kamambila nga khukholakana ni bikhuwambakho byesi kwabusa mumisomo kyositsana mu Uk ni Uganda.
Kamakhuwa kakhuwambakho ni liisina lyowo, kane bibonekhikho ni bareberesi namwe bebunyala khubona bari kumusomo kuli khusta bulayi.
Khamukhuwa khauambakho nga kabonisiwele khufurawo nga liisina lyowo lilikho gani khurusekho ate mbawo unamanya ta.

## Slide 18
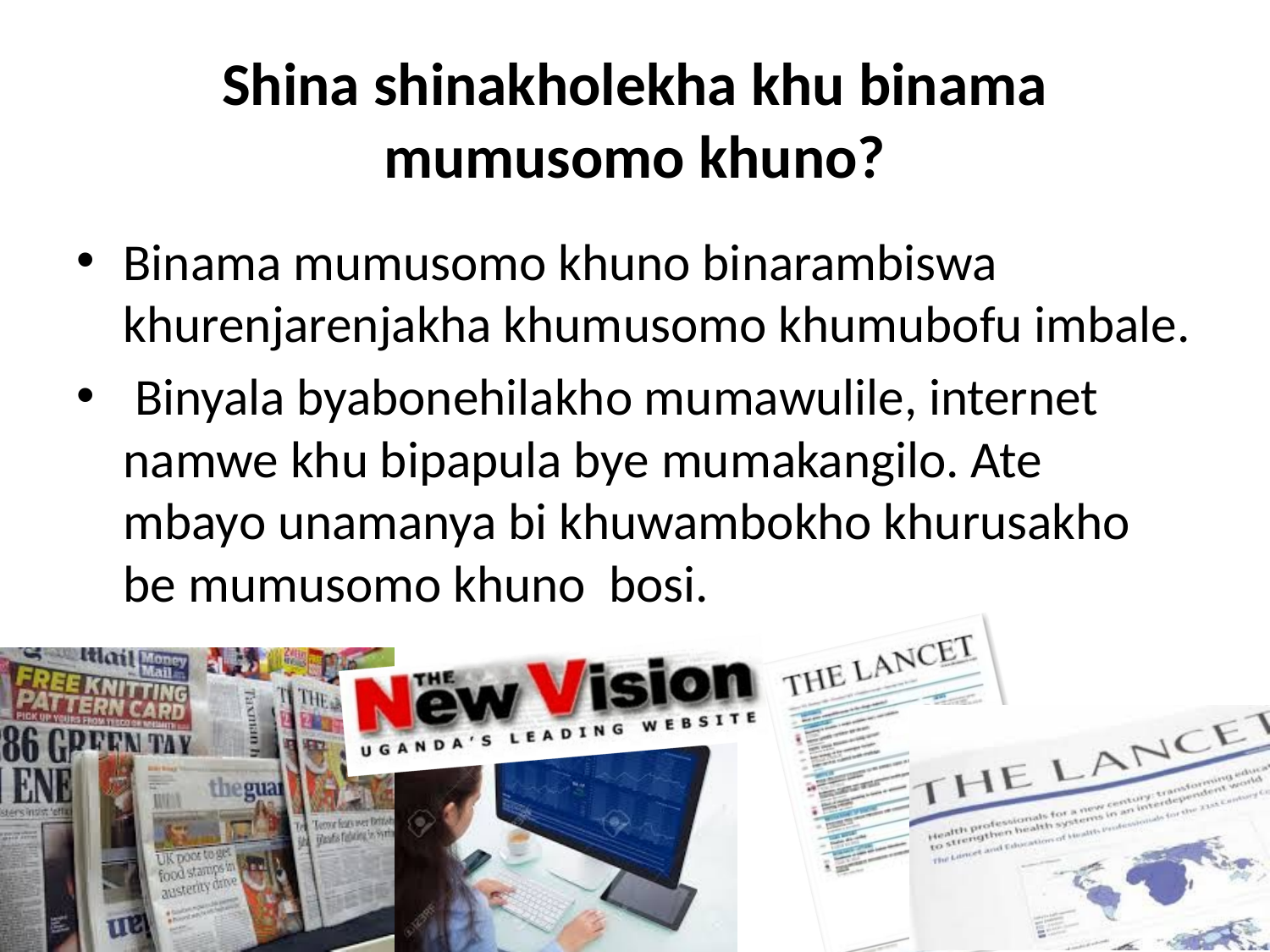

# Shina shinakholekha khu binama mumusomo khuno?
Binama mumusomo khuno binarambiswa khurenjarenjakha khumusomo khumubofu imbale.
 Binyala byabonehilakho mumawulile, internet namwe khu bipapula bye mumakangilo. Ate mbayo unamanya bi khuwambokho khurusakho be mumusomo khuno bosi.

## Slide 19
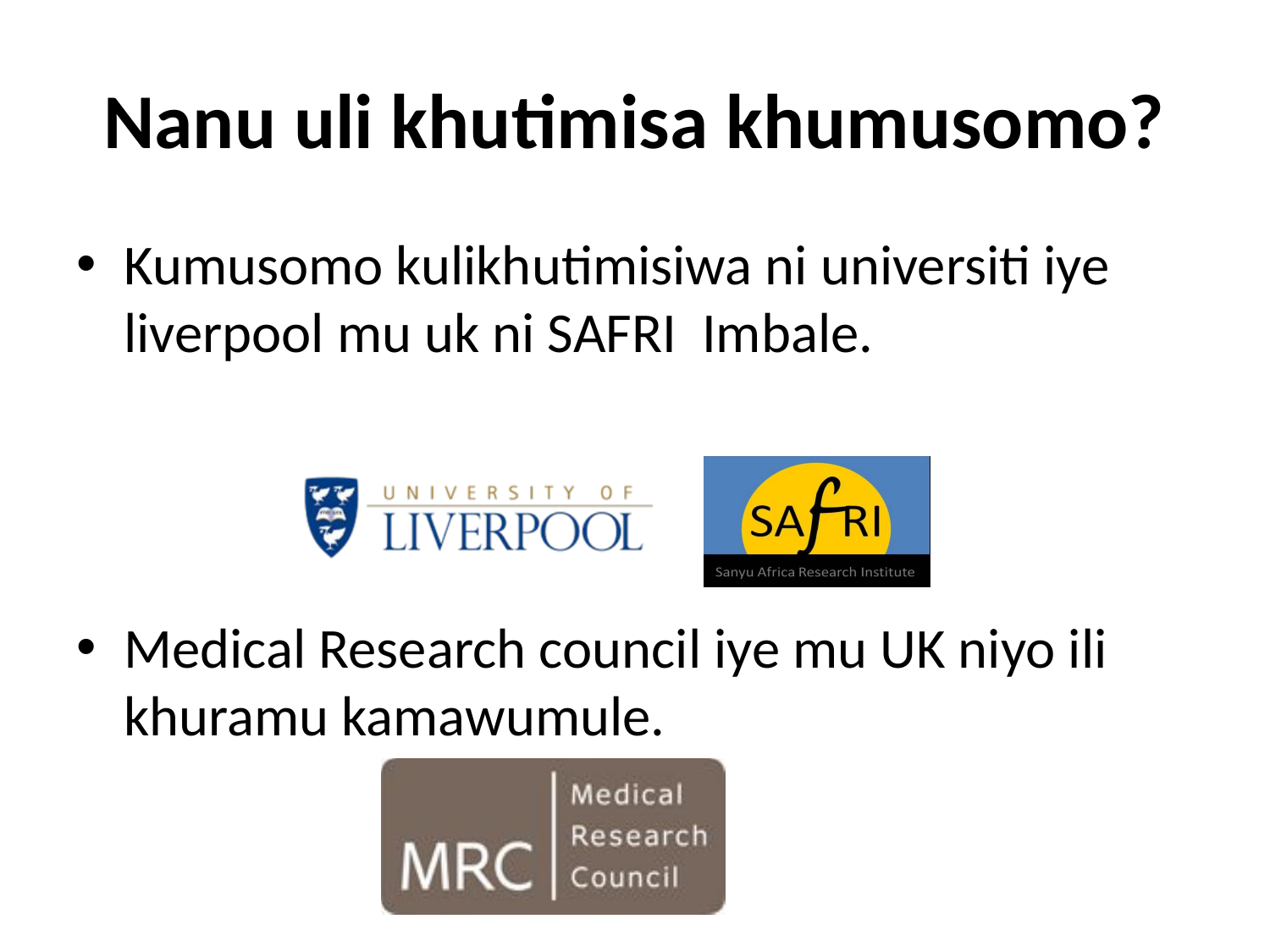

# Nanu uli khutimisa khumusomo?
Kumusomo kulikhutimisiwa ni universiti iye liverpool mu uk ni SAFRI Imbale.
Medical Research council iye mu UK niyo ili khuramu kamawumule.

## Slide 20
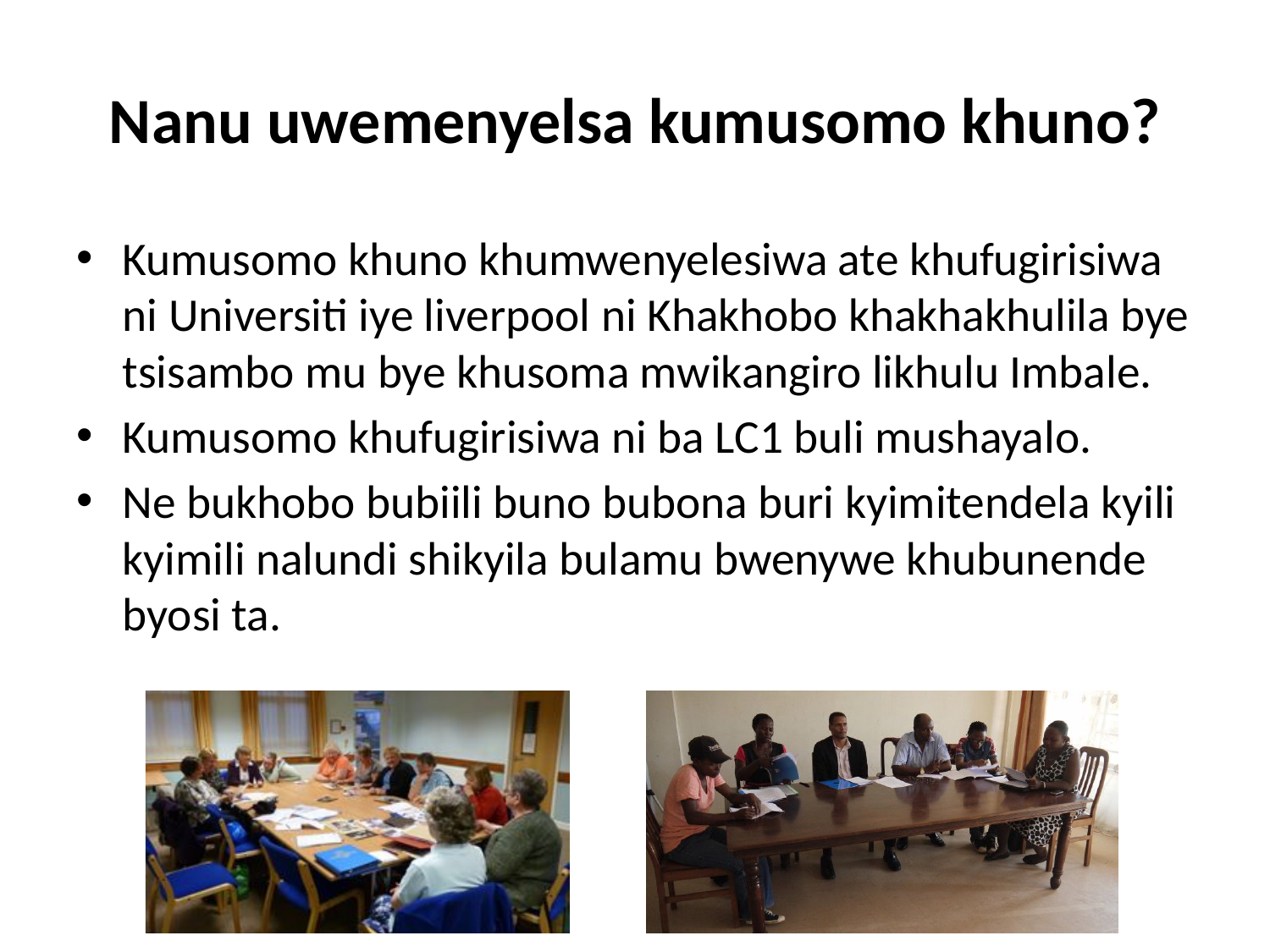

# Nanu uwemenyelsa kumusomo khuno?
Kumusomo khuno khumwenyelesiwa ate khufugirisiwa ni Universiti iye liverpool ni Khakhobo khakhakhulila bye tsisambo mu bye khusoma mwikangiro likhulu Imbale.
Kumusomo khufugirisiwa ni ba LC1 buli mushayalo.
Ne bukhobo bubiili buno bubona buri kyimitendela kyili kyimili nalundi shikyila bulamu bwenywe khubunende byosi ta.

## Slide 21
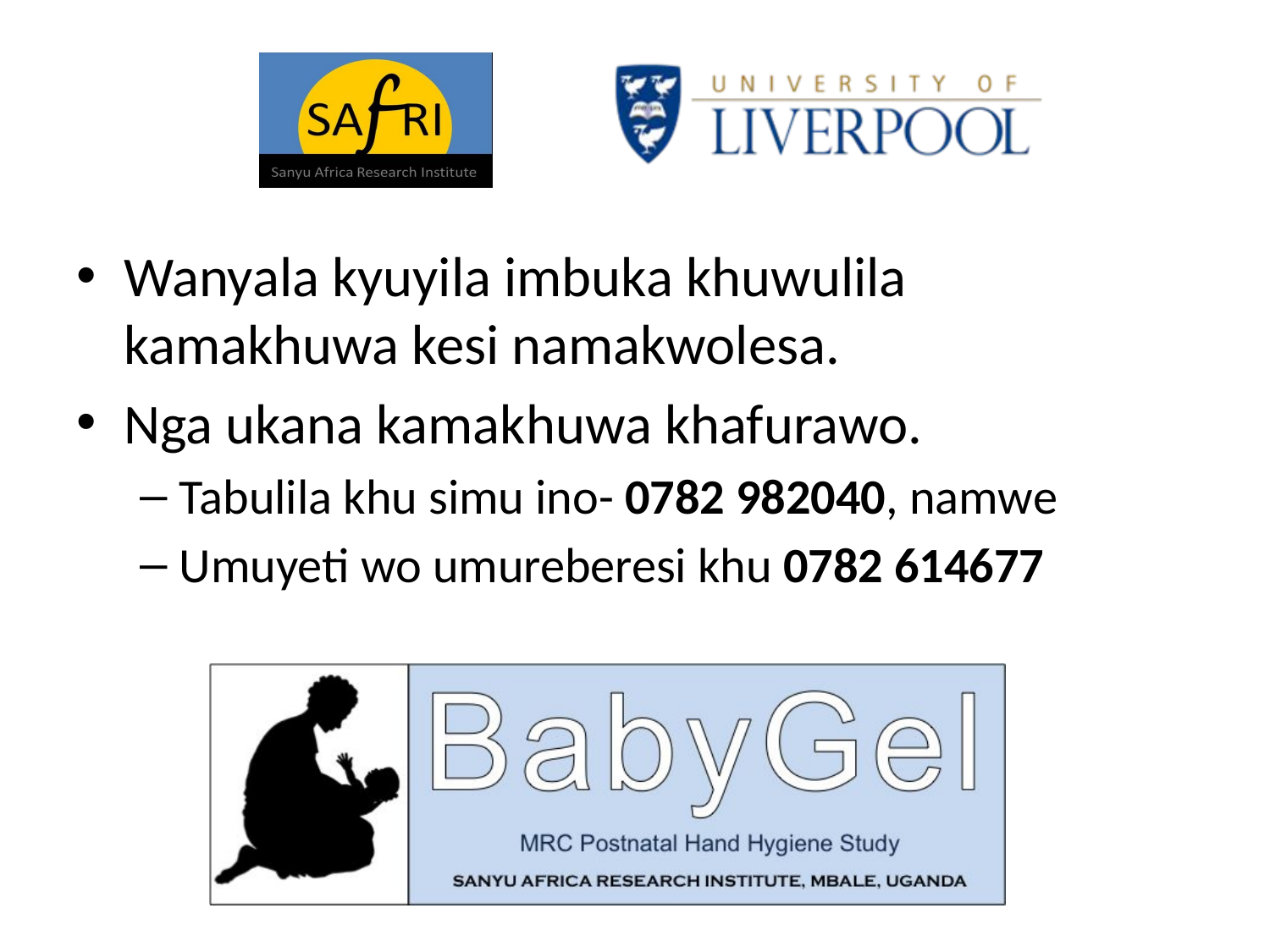

Wanyala kyuyila imbuka khuwulila kamakhuwa kesi namakwolesa.
Nga ukana kamakhuwa khafurawo.
Tabulila khu simu ino- 0782 982040, namwe
Umuyeti wo umureberesi khu 0782 614677
