# Supplementary material for: Optimising informed consent for participants in a randomised controlled trial in rural Uganda: a comparative prospective cohort mixed-methods study
Source: Trials. 2018 Dec 22;19:699. doi: 10.1186/s13063-018-3030-8 (PMC6304001; doi:10.1186/s13063-018-3030-8)
Supplement: Supplementary file 4 — The stepwise development process of informed consent models. (DOCX 19 kb) [file 13063_2018_3030_MOESM4_ESM.docx]

***Additional file 4***

**Development of the trial Information-Consent Models.**

The participant trial information, the slides used to make the flip charts and the videos were developed in a rigorous systematic stepwise process *(see supplementary 1)*

**Participant Information**

The participant information sheet (PIS) *(Additional 1)* was developed as part of the study protocol in simple English language with a Flesch readability score of 5.6. This PIS together with the protocol for BabyGel pilot trial was approved by Mbale Regional Hospital Ethics Committee and the University of Liverpool Ethics Committee and was registered with the Uganda National Council for Science in Technology (UNCST) in June 2015. The English PIS was later translated into Lumasaba. It is this PIS that was used in the standard or researcher –read consent model.

**Slideshow message consent**

The slide-show message in this study was developed from the study information on the PIS in a rigorous systematic stepwise process and presented on the flip charts, as described below namely:

*Step 1: The Illustrations*

AW and JD conceived the idea of putting the study information onto the slides. Draft sketches and illustrations were first drawn based on the study information, choosing an illustration that would best describe the text message in the PIS.

*Step 2: The Initial slides*

The whole text on the English PIS was collapsed or converted into bullet points on 15 initial slides in Microsoft Office PowerPoint, without pictures or illustrations. These initial slides were first shared amongst the research team in SAfRI and University of Liverpool to check for text integrity and accuracy.

*Step 3: Pictorial slides*

The text on each slide was then rearranged into flowcharts or diagrams. Pictures, obtained from the research teams own collections or from online databases were next obtained and incorporated where appropriate. These slides were then shared with the multidisciplinary research team which included two obstetricians, three paediatricians, 2-public health experts, 1-local ethics committee representative, 1- district health officer, 2-community advisory board members, 3-midwives, 2-nurses, 4-mothers and 4-research assistants who reviewed, commented, for the most appropriate texts and pictures to be included. The initial pictures in the slides were revised and changed according to local sensitivities, specific reviewer’s comments and interpretation. This was the most rigorous step of developing the slides with numerous changes, comments and additions into the slides. After 7 days of online discussion, the team had an agreed set of 21 slides which they believed were accurate, sensitive and appropriate to the local population.

The final version of the English slides was then translated into Lumasaba, the local language which was then shared with the SAfRI team and proofread by one Lumasaba-speaking member of the research staff and another independent member. The approved Lumasaba version was then submitted to another independent translator for backward translation into English. This final Lumasaba version was finally agreed by the research team after having the correlation of the backward English version with the forward English version. The final versions of the slides in both English and Lumasaba were approved by the research ethics committee in Uganda and UK.

*Step 4: Calendar-like flip charts*

The approved 21 pictorial slides in either English or Lumasaba *(Additional file 2)* were locally printed onto a ring-bound calendar-like ‘flip chart’. The slides were double printed onto the flip chart to enable the participant to look at the same information from one side as the research assistant reads the same information on the other side of the chart.

**Videos**

Videos were filmed and produced locally within the research offices in Mbale, Uganda.

The presenter (one of the research assistants) read the text from a scrolling laptop screen as the video was being filmed on the mobile smartphone. The final videos lasting 15minutes 38 seconds (English version) and 19 minutes 44 seconds (Lumasaba version) were released, reviewed by the research team and approved by the Research ethics committees. The approved videos *(Additional file 3)* were uploaded onto the Mobile smartphone and showed or played to the woman at the time of recruitment.
