# Supplementary material for: Optimising informed consent for participants in a randomised controlled trial in rural Uganda: a comparative prospective cohort mixed-methods study
Source: Trials. 2018 Dec 22;19:699. doi: 10.1186/s13063-018-3030-8 (PMC6304001; doi:10.1186/s13063-018-3030-8)
Supplement: Supplementary file 5 — Data collection tool: Optimal Informed Consent in the BabyGel pilot Cluster Randomised Trial. (DOCX 123 kb) [file 13063_2018_3030_MOESM5_ESM.docx]

***Additional file 5*** Data collection tool

|  | **Participant's Trial ID** | |  |  |  |  |
| --- | --- | --- | --- | --- | --- | --- |
|  | **Number of times the information was read/presented/shown to the participant** | |  |  |  |  |
|  |  | | | |  |  |
|  | **Which family members were present during the information-giving process? Please list all those present, along with their relationship to the participant.** | 1. | | | | |
|  |  | 2. | | | | |
|  |  | 3. | | | | |
|  |  | 4. | | | | |
|  |  | 5. | | | | |

| **A** | **OBJECTIVE ASSESSMENT: PARTICIPANT INFORMATION COMPOSITION** | | | |  |  |  |  |  |
| --- | --- | --- | --- | --- | --- | --- | --- | --- | --- |
|  | INSTRUCTIONS: Below there are several statements about the Informed Consent process undertaken in the BabyGel pilot study. Thinking about your participation in this clinical trial, please listen to each statement carefully. Then tell us whether you agree, OR disagree OR unsure of the statement. Please respond to each statement as best you can **without consulting your participant information sheet**.  We are interested in your opinions. | | | | | | | | |
|  | **Statement** | |  |  | |  |  |  |  |
| A1 | How long will you be followed up after giving birth as part of the study? | | 1  week | 2  weeks | | 1  month | 3 months | 6 months | Do not know |
| A2 | How many villages around Mbale are taking part in this study? | | 5 | 10 | | 20 | 50 | 100 | Do not know |
| A3 | If you have been given some hand gel and it runs out before the end of the study, what do you do? | Obtain more from Mbale Hospital pharmacy | Buy some more from a private pharmacy | Stop using the hand gel and use soap instead | | Phone the research team | Get more supplies from Busiu health centre or VHW | | Do not know |
| A4 | What should you do once you have given birth? | Notify the BabyGel research office | Notify the village health worker | Notify Mbale Hospital labour ward | | Notify the district health officer | Notify Mbale Hospital paediatric ward | | Do not know |
| A5 | What does the handgel contain? | Surgical alcohol | Distilled water | Soap | | Plain water | Saltwater | | Do not know |

| **B** | **UNDERSTANDING OF ASPECTS OF BabyGel Cluster Randomised Trial** | | | | | |
| --- | --- | --- | --- | --- | --- | --- |
|  | When you agreed to participate in your clinical trial, how well did you understand the following aspects of your clinical trial?  If you didn't understand the item at all, please circle 1. If you understood it very well, please circle 5. If you understood it somewhat, please circle a number between 1 and 5 | | | | | |
|  |  | I didn't understand this at all |  |  |  | I understood this very well |
| B1 | What the researchers are trying to find out in the study | 1 | 2 | 3 | 4 | 5 |
| B2 | How long you will be in the study | 1 | 2 | 3 | 4 | 5 |
| B3 | The treatments and procedures you will undergo | 1 | 2 | 3 | 4 | 5 |
| B4 | The alternatives to participation in the study | 1 | 2 | 3 | 4 | 5 |
| B5 | Whom you should contact if you have questions or concerns about the study | 1 | 2 | 3 | 4 | 5 |
| B6 | The fact that participation in the study is voluntary | 1 | 2 | 3 | 4 | 5 |
| B7 | Overall, how well did you understand the study when you signed the form? | 1 | 2 | 3 | 4 | 5 |

*Show the participant the alternative ways of providing information as part of the consent process (video or slideshow as necessary)*

| **C** | **PREFERENCE OF CONSENT MODEL** | | | | | |
| --- | --- | --- | --- | --- | --- | --- |
|  | Please rank the preference to the consent models in the range of 1 to 5, 1 being you don't prefer it at all, 5 being you preferred it most | I don't like this at all |  |  |  | I like this most |
| C1 | Researcher-read consent model | 1 | 2 | 3 | 4 | 5 |
| C2 | Slideshow consent model | 1 | 2 | 3 | 4 | 5 |
| C3 | Video consent model | 1 | 2 | 3 | 4 | 5 |
| C4 | Overall, which consent model do you prefer most and why? | | | | | |
